# 2022 Annual World Congress of the Pulmonary Vascular Research Institute

**DOI:** 10.1002/pul2.12153

**Published:** 2022-12-14

ABSTRACTS

## A001 PROGNOSTIC SIGNIFICANCE OF CARDIO‐ANKLE VASCULAR INDEX IN NEWLY DIAGNOSED IDIOPATHIC PULMONARY HYPERTENSION

GD Radchenko, IO Zhyvilo, EY Titov, YM Sirenko

*State Institution ‘National Scientific Center “Institute of Cardiology named after M. D. Strazhesko”’ of National Academy of Medical Science, Kyiv, Ukraine*

In previous studies, the cardio‐ankle vascular index (CAVI) was increased significantly in idiopathic pulmonary arterial hypertension (IPAH) patients compared with the healthy group and did not differ much from that in systemic hypertensives. In this study, the relationship between survival and CAVI was evaluated in patients with IPAH. We included 89 patients with newly diagnosed IPAH without concomitant diseases. Standard examinations, including right heart catheterization (RHC) and systemic arterial stiffness evaluation, were performed. All patients were divided according to CAVI value: the group with CAVI ≥ 8 (*n* = 18) and the group with CAVI < 8 (*n* = 71). The mean follow‐up was 33.8 ± 23.7 months. Kaplan–Meier and Cox regression analyses were performed for the evaluation of our cohort survival and the predictors of death. The group with CAVI ≥ 8 was older and more severe compared with the group having CAVI < 8. Patients with CAVI ≥ 8 had significantly reduced end‐diastolic (73.79 ± 18.94 vs. 87.35 ± 16.69 mL, *p* < 0.009) and end‐systolic (25.71 ± 9.56 vs. 33.55 ± 10.33 mL, *p* < 0.01) volumes of the left ventricle, higher right ventricle thickness (0.77 ± 0.12 vs. 0.62 ± 0.20 mm, *p* < 0.006) and lower tricuspid annular plane excursion (13.38 ± 2.15 vs. 15.98 ± 4.4 mm, *p* < 0.018). RHC data did not differ significantly between groups, except for the higher level of right atrial pressure in patients with CAVI ≥ 8 (11.38 ± 7.1 vs. 8.76 ± 4.7 mmHg, *p* < 0.08). The estimated overall survival rate was 61.2%. CAVI ≥ 8 increased the risk of mortality by 2.34 times (confidence interval 1.04–5.28, *p* = 0.041). The estimated Kaplan–Meier survival in patients with CAVI ≥ 8 was only 46.7 ± 7.18% compared with patients having CAVI < 8 (65.6 ± 4.2%, *p* = 0.035). At multifactorial regression analysis the CAVI reduced but retained its relevance as a predictor of death (odds ratio = 1.13, confidence interval 1.001–1.871). We suggest that CAVI could be a new independent predictor of death in the IPAH population and could be used to risk stratify this patient population better if CAVI is validated as a marker in a larger multicentre trial.

## A002 PORTOPULMONARY HYPERTENSION IN NONTRANSPLANTED PATIENTS: RESULTS OF THE LARGEST US SINGLE‐INSTITUTION REGISTRY

Hector R Cajigas, Charles D Burger, Rodrigo Cartin‐Ceba, Hilary M DuBrock, Karen Swanson, Hugo E Vargas, Andrew P Keaveny, Kimberly D Watt, Jie Na, Michael Krowka

*Pulmonary and Critical Care, Mayo Clinic, Rochester, MN, USA*

Portopulmonary Hypertension (PoPH) is a rare disease with poor prognosis affecting patients with liver disease. Medical therapy and, when feasible, liver transplantation (LT), improve outcomes. Clinical characteristics, risk profile and outcomes in the current era of pulmonary artery targeted therapies without LT are not well known. From the largest US, single‐institution registry of patients with PoPH, we analysed 160 patients between 1988 and 2019 who did not receive LT. Pulmonary arterial hypertension (PAH)‐pertinent characteristics, hemodynamics, treatments and risk stratification were compared at baseline, first follow‐up visit and at censor/death time. Median survival for the entire cohort was 27.5 months from the diagnosis of PoPH. Overall survival was 92, 89, 77, 62% and 38% at 3 and 6 months, 1, 2 and 5 years, respectively. Survival was significantly affected by the severity of liver disease (*p* < 0.001). Uncontrolled PAH was the most common reason for LT denial (108, 67%). The majority of patients received PAH‐specific therapies (136, 85%), mostly with monotherapy (123, 77%). With treatment, significant improvements were noted in World Health Organization (WHO) functional class (*p* = 0.04), 6‐min walk distance (*p* < 0.001), right ventricular function (*p* < 0.001), pulmonary vascular resistance (*p* < 0.001) and REVEAL lite‐2 score (*p* = 0.02) univariately. Per ESC risk stratification, no patient met full criteria for low risk at baseline or at follow‐up. In the multivariate regression risk model, 6‐min walk distance, right atrial pressure, pulmonary capillary wedge pressure, bilirubin level and model for end‐stage liver disease (MELD) ≥ 15 were associated with an increased risk of death. Patients with PoPH who did not undergo LT had a poor prognosis. This persisted despite utilization of PAH‐specific therapies, significant improvements in hemodynamics, echocardiography parameters of right ventricular function, 6‐min walk distance and WHO functional class.

## A003 THE INTERPLAY BETWEEN AQUAPORIN‐1 AND THE HYPOXIA‐INDUCIBLE FACTOR 1α In A LIPOPOLYSACCHARIDE‐INDUCED MODEL IN HUMAN PULMONARY MICROVASCULAR ENDOTHELIAL CELLS

Chrysi Keskinidou, Alice G Vassiliou, Ioanna Dimopoulou, Anastasia Kotanidou, Stylianos E Orfanos

*First Department of Critical Care Medicine & Pulmonary Services, Medical School of National and Kapodistrian University of Athens, Evangelismos Hospital, Athens, Greece*

Aquaporin‐1 (Aqp1), which is expressed in the pulmonary microvascular endothelium, can be upregulated by hypoxia, a common cause of various pathologic conditions, such as multiorgan failure and pulmonary arterial hypertension. Upregulation leads to increased cell water permeability in the lungs. Its role in osmotic water movement and edema formation makes Aqp1 an important mediator of both physiological and pathological cell mechanisms. On the contrary, the hypoxia‐inducible factor 1α (HIF‐1α) has been shown to be implicated in acute lung injury responses, modulating pulmonary vascular leakage. We hypothesized that the Aqp1 and HIF‐1α systems interact, affecting the expression and function of Aqp1 in human pulmonary microvascular endothelial cells (HPMECs) exposed to lipopolysaccharide (LPS). To this end, HPMECs were exposed to LPS for 24 h, and the protein expression and function of Aqp1 were examined. In a different set of experiments, HPMECs were silenced for *HIF‐1α* and then exposed to LPS for 24 h, and Aqp1 protein levels and function were explored by western blot and permeability assays, respectively. Aqp1 expression was higher in HPMECs exposed to LPS for 24 h compared with control cells (3.22 ± 1.4 vs. 0.90 ± 0.68, respectively; *p* = 0.005). In the *HIF‐1α*‐silenced cells exposed to LPS, the expression of Aqp1 was not increased (*p* > 0.05). In the permeability experiments, different osmotic swellings were observed. A statistically significant volume increase was observed at the 6 min time point in the control HPMECs, the LPS‐exposed HPMECs and the siRNA negative control cells. Of note, the LPS‐exposed cells exhibited a statistically greater volume increase compared with the control cells. HPMECs exposed to the Aqp1 blocker HgCl_2_ exhibited no cell volume increase, as expected. Most importantly, the HIF‐1α‐silenced cells exposed to LPS did not exhibit swelling, implying a dysfunctional Aqp1. Based on our results, it seems that *HIF‐1α* silencing negatively affects Aqp1 protein expression and function.

## A004 PHYSICAL ACTIVITY MEASURED BY ACCELEROMETRY QUALIFIES AS CLINICAL TRIAL ENDPOINT IN PEDIATRIC PULMONARY HYPERTENSION

Mark‐Jan Ploegstra, Marlies G Haarman, Suzanne SJ Schwartz, Theresia R Vissia‐Kazemier, Marcus TR Roofthooft, Johannes M Douwes, Rolf MF Berger

*Dutch National Network for Pediatric Pulmonary Hypertension, Center for Congenital Heart Diseases, Beatrix Children's Hospital, University Medical Center Groningen, Groningen, The Netherlands*

Pediatric pulmonary hypertension (PH) is a severe incurable disease with a poor prognosis. In pediatric PH, trial design is hampered by the absence of age‐appropriate trial endpoints. This study aimed to evaluate physical activity measured by accelerometry (PA‐accelerometry) as a trial endpoint in pediatric PH, by assessing its association with disease severity and long‐term outcome and by demonstrating how PA‐accelerometry changes correspond to changes in disease severity and outcome. A total of 48 children with hemodynamically confirmed PH from the prospective Dutch National Network for Pediatric PH wore a tri‐axial accelerometer for ≥3 days. Z‐scores of counts per minute (CPM‐Z) and the percentage of time per day spent in moderate or vigorous PA‐accelerometry intensity levels (%MVPA‐Z) were defined as accelerometer outputs. Disease severity was defined according to 6‐min walk distance Z‐scores (6MWD‐Z), World Health Organization functional class (WHO‐FC) and N‐terminal pro‐B‐type natriuretic peptide (NT‐proBNP). Adverse outcome was defined as death or lung transplantation. At baseline, lower CPM‐Z was associated with higher WHO‐FC (*p* < 0.001), lower 6MWD‐Z (*p* < 0.001) and higher NT‐proBNP (*p* = 0.012). Likewise, %MVPA‐Z was associated with WHO‐FC, 6MWD‐Z and NT‐proBNP (*p* < 0.001, *p* < 0.001 and *p* = 0.046, respectively). These results remained significant after adjusting for age/sex and could be replicated throughout the long‐term disease course. Changes in %MVPA‐Z over time tended to be correlated with changes in 6MWD‐Z (*p* = 0.064). During a median follow‐up of 4.4 years, eight children died and four underwent lung transplantation. Lower CPM‐Z (hazard ratio 0.51, *p* = 0.024) and %MVPA‐Z (hazard ratio 0.44, *p* = 0.017) predicted adverse outcome independent of age/sex. Time‐dependent changes of CPM‐Z and %MVPA‐Z were also correlated with outcome (*p* = 0.04). PA‐accelerometry predicts disease severity and adverse outcome, both at baseline and throughout the disease course of PH. These findings support the usefulness of PA‐accelerometry as a safe and feasible clinical trial endpoint in pediatric PH.

## A005 IMPACT OF COMORBIDITIES IN PATIENTS WITH PULMONARY ARTERIAL HYPERTENSION

Athiththan Yogeswaran, Khodr Tello, Natascha Sommer, Zvonimir Rako, Hossein Ardeschir Ghofrani, Werner Seeger, Manuel J Richter, Henning Gall

*Department of Internal Medicine, Justus‐Liebig‐University Giessen, Universities of Giessen and Marburg Lung Center (UGMLC), Member of the German Center for Lung Research (DZL), Giessen, Germany*

Recent studies suggest that there might be a continuum between group 1 (pulmonary arterial hypertension, PAH) and group 2 pulmonary hypertension (PH attributable to left heart disease). Patients in between show a specific pattern of comorbidity and are classified as PAH with cardiac phenotype. However, it is unclear whether comorbidities have an impact on mortality and risk parameters in idiopathic PAH. We analysed all patients with idiopathic PAH (IPAH) who were entered in the Giessen PH registry. Patients were divided into two groups based on their comorbidity profile. We then examined the influence of comorbidities on survival. Four hundred and thirty‐eight patients were included in this study, of whom 76 patients (17%) were classified as IPAH with cardiac phenotype (cIPAH). The cIPAH patients were significantly older and showed higher pulmonary arterial wedge pressure, while mPAP and PVR were lower. Notably, patients with cIPAH exhibited more often a post‐capillary component during follow‐up right heart catheterization (3% of IPAH with pulmonary phenotype patients versus 11% of cIPAH patients, *χ* ^2^ = 0.035). Low body mass index (BMI), chronic coronary syndrome (CCS) and glycated haemoglobin (HbA1c) were associated with mortality in IPAH, whereas arterial hypertension and diabetes mellitus (per se) did not predict survival. Patients diagnosed with cIPAH had significantly impaired survival rates. Age, BMI, CCS, SvO_2_, HbA1c and creatinine were associated with mortality in cIPAH (*p* < 0.05, respectively). Interestingly, backward Cox regression revealed that solely age, HbA1c, creatinine and SvO_2_ were independent predictors of mortality in cIPAH. A survival model based on these parameters predicted mortality in cIPAH (5‐year survival of low‐, intermediate‐ and high‐risk group: 78%, 30% and 30%, log‐rank *p* = 0.008). Spline plots revealed optimal HbA1c ~6%. Survival of IPAH patients is affected by comorbidities. Risk stratification in cIPAH based on age, HbA1c, creatinine and SvO_2_ is highly predictive. Clinicians therefore need to focus on treatment of comorbidities and rethink risk stratification in patients with cIPAH.

## A006 EVALUATION AND CLINICAL VALUE OF LIPID METABOLITES IN THE PROGNOSIS OF ACUTE PULMONARY THROMBOEMBOLISM

Wenhao Chen, Yunxia Li, Dan Guo, Shuyue Xia

*Department of Respiratory and Critical Care Medicine, Central Hospital Affiliated to Shenyang Medical College, Shenyang 10024, China*

With the improvement of diagnostic methods and treatment levels, the mortality rate of patients with pulmonary thromboembolism (PTE) has decreased. However, at home and abroad, its mortality rate is second only to ischemic heart disease and stroke, ranking third. The clinical transition of patients with pulmonary embolism is intricate. Even if the initial hemodynamic state is stable, the inpatient mortality rate can still reach 8–30%. Therefore, it is of great significance to find suitable biological indicators for PTE evaluation and prognosis. The level of serum lipid metabolism is easy to detect and economical in clinic, and it has been confirmed to have a clear relationship with arterial thrombosis, but there is no certainty about whether it is related to venous thrombosis. The purpose of this study was to explore the effectiveness and clinical value of serum triglycerides, cholesterol levels and the neutrophil/lymphocyte ratio in predicting the prognosis of patients with pulmonary embolism. One hundred and forty‐two patients with pulmonary thromboembolism were selected from 2016 to 2021 in the Central Hospital Affiliated to Shenyang Medical College. Clinical data of the patients were collected. The patients were divided into death and survival groups according to whether patients died 30 days after admission, and the differences in serum triglycerides, cholesterol levels and neutrophil/lymphocyte ratios were compared between the two groups at the time of admission. Single‐factor and multi‐factor non‐conditional logistic regression analysis was used to explore the risk factors affecting death in PTE patients. The neutrophil/lymphocyte ratio in the survival group was significantly lower than that in the death group (*p* < 0.05) in terms of serum total cholesterol, low‐density lipoprotein cholesterol and triglycerides; the high‐density lipoprotein cholesterol was significantly higher than that in the death group (*p* < 0.05). The serum triglycerides, cholesterol levels and neutrophil/lymphocyte ratio of patients at admission are closely related to the prognosis of patients and can be used to guide clinicians to evaluate patients with pulmonary embolism to carry out early intervention and improve the prognosis of patients.

## A007 POST‐COVID‐19 PATIENTS SHOW AN INCREASED ENDOTHELIAL PROGENITOR CELL PRODUCTION

P Poyatos, N Luque, L Sebastián, M Bonnin, S Eizaguirre, G Sabater, M Peracaula, R Orriols, O Tura‐Ceide

*Girona Biomedical Research Institute (IDIBGI), Girona, Spain and Department of Pulmonary Medicine, Dr Josep Trueta University Hospital of Girona and Santa Caterina Hospital of Salt, Girona, Spain*

Severe acute respiratory syndrome coronavirus 2 (SARS‐CoV‐2), the cause of coronavirus disease 2019 (COVID‐19), has generated a global emergency situation. The endothelium is a target of SARS‐CoV‐2, generating endothelial dysfunction and an unbalanced vascular homeostasis. It is considered that the number of endothelial progenitor cells (EPCs) acts as an indicator of vascular damage. However, its role in SARSCoV‐2 is unknown. Increased production of EPCs 3 months after SARS‐CoV‐2 infection would indicate the presence of vascular sequelae in these patients. The aim of this study was to quantify EPCs and assess, for the first time, whether there is a significant increase after SARS‐CoV‐2 infection. We wanted to evaluate whether there are differences in the number of EPCs in post‐COVID‐19 patients who have suffered an acute pulmonary embolism (PE) and to determined whether this increase is correlated with any of the clinical parameters studied. A total of 33 patients were recruited into the study 3 months after overcoming COVID‐19 and 19 healthy controls. Of the 33 COVID‐19 patients, 14 had suffered PE. EPCs were obtained from mononuclear cells isolated from peripheral blood, cultured with specific medium and conditions. The results show a significant increase (*p* = 0.04) in the appearance of EPCs in patients 3 months post‐COVID‐19 compared with healthy controls. However, there were no differences in the number of EPCs in COVID‐19 patients depending on whether or not PE was detected. Patients with a high number of EPCs showed high levels of hemoglobin and troponin, but they did not show a relationship with any other parameter studied. The results obtained confirm the presence of vascular sequelae in post‐COVID‐19 patients.

## A008 THE ALTERATIONS OF MOLECULAR MARKERS AND SIGNALING PATHWAYS IN CHRONIC THROMBOEMBOLIC PULMONARY HYPERTENSION, A STUDY WITH TRANSCRIPTOME SEQUENCING AND CERNA (lncRNA–miRNA–mRNA) NETWORK CONSTRUCTION

Wenqing Xu, Min Liu

*Peking University School of Clinical Medicine, China; China‐Japan Friendship Hospital, China*

At present, the alterations of molecular markers and signaling pathways in patients with chronic thromboembolic pulmonary hypertension (CTEPH) remain unclear. We attempted to compare the differences in molecular markers and signaling pathways in CTEPH patients and healthy people with transcriptome sequencing and ceRNA (lncRNA–miRNA–mRNA) network construction. We prospectively included 12 patients with CTEPH and 10 sex‐ and age‐matched healthy people as controls. We extracted RNA from whole blood samples to construct the library. Then, qualified libraries were sequenced using the PE100 strategy on the BGIseq platform. Subsequently, the DESeq. 2 package in R was used to screen differentially expressed mRNAs (DEmRNAs) and differentially expressed lncRNAs (DElncRNAs) of seven CTEPH patients and five healthy volunteers. Afterwards, we performed functional enrichment and protein–protein interaction analysis of DEmRNAs. We also performed lncRNA–mRNA co‐expression analysis and lncRNA–miRNA–mRNA network construction. In addition, we performed diagnostic analysis on the GSE130391 data set. Finally, we performed in vitro validation of genes in five CTEPH patients and five healthy volunteers. A total of 437 DEmRNAs and 192 DElncRNAs were obtained. Subsequently, 205 pairs of interacting DEmRNAs and 232 pairs of lncRNA–mRNA relationships were obtained. DEmRNAs were significantly enriched in the chemokine signaling pathway, metabolic pathways, arachidonic acid metabolism and the MAPK signaling pathway. Only one regulation pathway of SOBP‐has‐miR‐320b‐LINC00472 was found through ceRNA network construction. In diagnostic analysis, the area under the curve of LINC00472, PIK3R6, SCN3A and TCL6 was 0.964, 0.893, 0.750 and 0.732, respectively. The identification of alterations of molecules and pathways may provide further research directions on pathogenesis of CTEPH.

## A009 MATRICELLULAR PROTEIN SPARC: A NOVEL KEY PLAYER IN PULMONARY HYPERTENSION

Christine Veith, Ipek Vartürk‐Özcan, Magdalena Wujak, Stefan Hadzic, Cheng‐Yu Wu, Fenja Knoepp, Simone Kraut, Aleksandar Petrovic, Marija Gredic, Oleg Pak, Monika Brosien, Marie Heimbrodt, Jochen Wilhelm, Friederike C Weisel, Kathrin Malkmus, Katharina Schäfer, Henning Gall, Khodr Tello, Djuro Kosanovic, Akylbek Sydykov, Akpay Sarybaev, Andreas Günther, Ralf P Brandes, Werner Seeger, Friedrich Grimminger, Hossein A Ghofrani, Ralph T Schermuly, Grazyna Kwapiszewska, Natascha Sommer, Norbert Weissmann

*Excellence Cluster Cardio‐Pulmonary Institute, University of Giessen and Marburg Lung Center, Member of the German Center for Lung Research and Institute for Lung Health Justus‐Liebig‐University, Giessen, Germany; Department of Medicinal Chemistry, Collegium Medicum in Bydgoszcz, Nicolaus Copernicus University in Toruń, Poland, Department of Pulmonology, I. M. Sechenov First Moscow State Medical University (Sechenov University), Moscow, Russia; Kyrgyz National Center for Cardiology and Internal Medicine and Kyrgyz Indian Mountain Biomedical Research Center, Bishkek, Kyrgyz Republic; Institute for Cardiovascular Physiology, Goethe University, Frankfurt am Main, Germany; Ludwig Boltzmann Institute for Lung Vascular Research and Otto Loewi Center, Physiology, Medical University of Graz, Graz, Austria*

Pulmonary hypertension (PH) is a life‐threatening disease, characterized by excessive pulmonary vascular remodeling, leading to elevated pulmonary arterial pressure and right heart hypertrophy. Pulmonary hypertension can be caused by chronic hypoxia, leading to hyperproliferation of pulmonary arterial smooth muscle cells (PASMCs) and apoptosis‐resistant pulmonary microvascular endothelial cells (PMVECs). Upon re‐exposure to normoxia, chronic hypoxia‐induced PH in mice is reversible. In this study, we aim to identify novel candidate genes involved in pulmonary vascular remodeling specifically in the pulmonary vasculature. C57BL/6 J mice were exposed to normoxia, chronic hypoxia or chronic hypoxia with subsequent re‐exposure to normoxia for different time points. Microarray analysis of the pulmonary vascular compartment after laser microdissection identified secreted protein acidic and rich in cysteine (*Sparc*) as one of the genes downregulated at all reoxygenation time points investigated. Intriguingly, SPARC was, vice versa, upregulated in lungs during development of hypoxia‐induced PH in mice and in idiopathic pulmonary arterial hypertension (IPAH), although SPARC plasma levels were not elevated in PH. Transforming growth factor (TGF)‐β1 or hypoxia‐inducible factor (HIF)‐2A signaling pathways induced SPARC expression in human PASMCs. In loss‐of‐function studies, *SPARC* silencing enhanced apoptosis and reduced proliferation. In gain‐of‐function studies, elevated SPARC levels induced PASMC but not PMVEC proliferation. Coculture and conditioned medium experiments revealed that PMVEC‐secreted SPARC acts as a paracrine factor triggering PASMC proliferation. *In vivo*, adeno‐associated virus‐mediated *Sparc* knockdown in adult mice significantly improved hemodynamic and cardiac function in PH mice. Our study provided evidence for the involvement of SPARC in the pathogenesis of human PH and chronic hypoxia‐induced PH in mice, most probably by affecting vascular cell function.

## A010 ROLE OF CNPY2 IN MEDIATING SKELETAL MUSCLE–PULMONARY VASCULATURE CROSSTALK IN PULMONARY HYPERTENSION ASSOCIATED WITH HEART FAILURE WITH PRESERVED EJECTION FRACTION

*Jia‐Rong Jheng, Andrea L Frump, Gunner Halliday, Todd Cook, Amanda Fisher, Marc Simon, Yen‐Chun Lai*

*Division of Pulmonary, Critical Care, Sleep and Occupational Medicine, Indiana University School of Medicine, Indianapolis, IN, USA; Division of Cardiology, University of California, San Francisco, San Francisco, CA, USA; Department of Anatomy, Cell Biology and Physiology, Indiana University School of Medicine Indianapolis, IN, USA*

The unfolded protein response (UPR) in the endoplasmic reticulum (ER) plays a critical role in controlling cell fate decisions under ER stress. However, whether ER stress and UPR sensors are involved in the regulation of pulmonary hypertension associated with heart failure with preserved ejection fraction (PH‐HFpEF) remains unknown. Recently, we reported on a role of skeletal muscle sirtuin‐3 (SIRT3) in modulating PH‐HFpEF. Here, using skeletal muscle‐specific *Sirt3* knockout mice (*Sirt3* ^skm−/−^), we found that expression levels of canopy fibroblast growth factor signaling regulator 2 (CNPY2), a UPR initiator known to induce excessive angiogenesis and proliferation, were increased in pulmonary artery endothelial cells (PAECs) isolated from *Sirt3* ^skm−/−^ mice. Enhanced CNPY2 expression levels were also found in PAECs isolated from the SU5416/Obese ZSF1 (Ob‐Su) rat model of PH‐HFpEF. Overexpression of CNPY2 markedly increased cell migration and proliferation in PAECs. Using global mass spectrometry‐based comparative secretome analysis, we found increased protein abundance levels of lysyl oxidase homolog 2 (LOXL2) and beta‐2‐microglobulin (B2M) in SIRT3‐deficient skeletal muscle cells. Elevated circulating levels and protein expression levels of LOXL2 and B2M were also observed in plasma and skeletal muscle of *Sirt3* ^skm−/−^ mice, Ob‐Su rats and patients with PH‐HFpEF. Treatment with recombinant proteins of LOXL2 and/or B2M resulted in increased CNPY2 expression, concomitant with increased cell migration and proliferation in PAECs. Together, these data reveal a potential role of the UPR initiator CNPY2 in mediating skeletal muscle–pulmonary vasculature crosstalk in PH‐HFpEF through the regulatory axis of skeletal muscle SIRT3, LOXL2 and B2M.

## A011 A *TM4SF1*‐MARKED SUBPOPULATION OF ENDOTHELIAL STEM/PROGENITOR CELLS IDENTIFIED BY LUNG SINGLE‐CELL OMICS OF PULMONARY ARTERIAL HYPERTENSION

Jason Hong, Brenda Wong, Caroline Huynh, Brian Tang, Soban Umar, Xia Yang, Mansoureh Eghbali

*Division of Pulmonary and Critical Care Medicine, Departmento of Medicine; Department of Integrative Biology and Physiology; Department of Anesthesiology & Perioperative Medicine; Department of Integrative Biology and Physiology, University of California, Los Angeles, Los Angeles, CA, USA*

The identification and role of endothelial progenitor cells (EPCs) in pulmonary arterial hypertension (PAH) remains controversial. Single‐cell omics analysis can shed light on EPCs and their potential contribution to PAH pathobiology. We aimed to identify EPCs in rat lungs and assess their relevance to preclinical and human PAH. Differential expression, gene set enrichment, cell‐to‐cell communication and trajectory reconstruction analyses were performed on lung endothelial cells from single‐cell RNA‐seq of Sugen‐hypoxia, monocrotaline and control rats. Relevance to human PAH was assessed in multiple independent blood and lung transcriptomic datasets. A subpopulation of endothelial cells (EA2) marked by *Tm4sf1*, a gene strongly implicated in cancer, harbored a distinct transcriptomic signature, including *Bmpr2* downregulation, that was enriched for pathways such as inflammation and angiogenesis. Cell‐to‐cell communication networks specific to EA2, such as CXCL12 signaling, were activated in PAH. Trajectory analysis demonstrated that EA2 has a stem/progenitor cell phenotype. Analysis of independent datasets revealed that *Tm4sf1* is a marker for hematopoietic stem cells and is upregulated in peripheral blood in PAH, particularly in patients with worse World Health Organization functional class. EA2 signature genes, including *Procr* and *Sulf1*, were found to be differentially regulated in the lungs of PAH patients and in PAH models in vitro, such as BMPR2 knockdown. Our study uncovered a novel *Tm4sf1*‐marked stem/progenitor subpopulation of rat lung endothelial cells and demonstrated its relevance to preclinical and human PAH. Future experimental studies are warranted to elucidate further the pathogenic role and therapeutic potential of targeting EA2 and *Tm4sf1* in PAH.

## A012 BONE MORPHOGENIC PROTEIN RECEPTOR 2 MUTATIONS IN SOUTH INDIAN CHILDREN WITH PULMONARY ARTERIAL HYPERTENSION

Shine Kumar, Lalitha Biswas, Chethampadi Gopi Mohan, Raman Krishna Kumar

*Department of Pediatric Cardiology and Pulmonary Hypertension Services; Centre for Nanosciences and Molecular Medicine, Amrita Institute of Medical Sciences; Department of Pediatric Cardiology, Amrita Institute of Medical Sciences, Amrita Vishwa Vidyapeetham, Kochi, Kerala, India*

Bone morphogenic protein receptor 2 (*BMPR2*) mutations are described in children with pulmonary arterial hypertension (PAH) from select populations. The aim was to describe the characteristics of *BMPR2* mutations in children (<18 years of age) from South India. All children with a diagnosis of idiopathic PAH (IPAH), hereditary PAH (HPAH) and PAH disproportionate to congenital heart disease (CHD) were included. *BMPR2* mutation was analysed in a peripheral vein blood sample by the Sanger sequencing method. The study period was from January 2018 to October 2019. We identified *BMPR2* mutations in seven children among 16 (43.7%) analysed, the majority being females (five, 71.4%). The median age at diagnosis was 9.5 years (range 0.7–17 years). The diagnosis was IPAH (four of seven, 57.1%), HPAH (two of seven, 28.5%) and PAH with associated CHD (one of seven, 14.2%). All were heterozygous, with missense (four, 57.1%), nonsense (two, 28.5%) and 3′ UTR (one, 14.2%) mutations. Mutations were found in exon 3, exon 7, exon 9, exon 11, exon 12 and the 3′ UTR regions of the *BMPR2* gene. The HPAH patients had mutation with autosomal dominant inheritance and variable penetrance. All patients had severe PAH (mean PA pressure 71 ± 22.4 mmHg, pulmonary vascular resistance index 25.5 ± 10.1 Wood units.m^2^) at diagnosis. After a mean follow‐up period of 43.7 ± 21.3 months, there was one mortality (14.2%) from progressive right ventricular failure. The rest were on dual therapy, with suboptimal hemodynamic response. In a South Indian population, children with *BMPR2* mutations often present late in childhood with female preponderance and severe PAH. The response to initial targeted dual therapy appears limited.

## A013 ECHOCARDIOGRAPHIC EVALUATION OF RIGHT VENTRICULAR DIASTOLIC FUNCTION IN PULMONARY HYPERTENSION

Athiththan Yogeswaran, Zvonimir A Rako, Selin Yildiz, Hossein Ardeschir Ghofrani, Werner Seeger, Henning Gall, Nils C Kremer, Manuel J Richter, Khodr Tello

*Department of Internal Medicine, Justus‐Liebig‐University Giessen, Universities of Giessen and Marburg Lung Center (UGMLC), Member of the German Center for Lung Research (DZL), Giessen, Germany*

Right ventricular (RV) diastolic function is important for both symptoms and survival in patients with pulmonary hypertension (PH). However, assessing RV diastolic function is complex, requiring conductance catheterization for pressure–volume (PV) loop analysis. We therefore investigated whether non‐invasively derived echocardiographic parameters can be used to assess RV diastolic function in patients with PH. Prospectively, we recruited consecutive patients with (clinically suspected) PH who underwent right heart catheterization including PV loop assessment. Correlation was assessed using Spearman's rho. Sensitivity, specificity, positive and negative predictive values were calculated using thresholds derived via Youden index in receiver operating characteristic (ROC) curve analyses. For survival analyses, patients included in the Giessen PH registry were analysed retrospectively. All participating patients gave written informed consent, and the investigation was approved by the ethics committee of the Faculty of Medicine at the University of Giessen (NCT04663217). Sixty patients were included in the prospective part of the study, 26% of whom were diagnosed with pulmonary arterial hypertension, 8% with pulmonary venous hypertension and 28% with chronic thromboembolic PH. Thirty‐six per cent of the included patients had mPAP < 25 mmHg. Commonly used parameters, such as tricuspid E/A and E/E′, were not correlated with gold‐standard end‐diastolic elastance (Eed) derived from PV loops. We introduced the ratio between lateral tricuspid annulus peak systolic velocity and right atrial end‐systolic area (indexed for body surface area) as a new surrogate parameter for diastolic function (S′/RAAi). The S′/RAAi was correlated with Eed (rho = −0.50, *p* < 0.001) and end‐diastolic pressure (EDP; rho = −0.49, *p* < 0.001). Concordantly, ROC analyses showed high area under the curve for detection of increased Eed [AUROC: 0.913 (0.839, 0.986)] and EDP [AUROC: 0.848 (0.699, 0.998)]. Accordingly, S′/RAAi precisely excluded high Eed and high EDP (negative predictive values: 98% and 95%, respectively). Patients with low S′/RAAi (indicating enhanced diastolic stiffness) showed significantly reduced survival rates in Cox regression and Kaplan–Meier analysis in 225 patients with precapillary PH enrolled in the Giessen PH registry. In conclusion, the S′/RAAi is a novel and accurate indicator of RV diastolic function and predicts mortality in patients with PH.

## A014 INCREASE IN PROGENITOR ENDOTHELIAL CELLS ASSOCIATED WITH RIGHT HEART FAILURE IN PEDIATRIC PATIENTS WITH SECONDARY PULMONARY ARTERIAL HYPERTENSION

Humberto García Aguilar, Juan Antonio Suárez Cuenca, Jose Luis Aceves Chimal

*Centro Médico Nacional 20 de Noviembre ISSSTE, México City, Mexico*

Pulmonary arterial hypertension (PAH) is a serious condition that causes right heart failure. A persistent inflammatory process is present in the vascular endothelial tissue, which releases circulating CD34^+^,133^+^ endothelial progenitor cells (EPCs), possibly as a mechanism of response to vascular damage. Using flow cytometry with MACS Quant Analyzer 10 equipment, the number and percentage of CD34^+^,133^+^ EPCs were determined in a cohort of 30 pediatric patients with PAH associated with congenital heart disease with right heart failure (*n* = 16) and without (*n* = 14). The population were male (56.5%), aged 8.4 ± 5.9 years old, and 4% carried Down syndrome. The EPCs showed a significant correlation with pulmonary mean arterial pressure (rho = 0.49, *p* = 0.04) and a negative correlation with oxygen saturation (rho = −0.75, *p* < 0.01) in the subgroup with right ventricle failure. Finally, the percentage and total number of CD34^+^,133^+^ EPCs showed significant diagnostic ability to discriminate right ventricle maladaptation (area under the curve = 0.85, *p* = 0.006 and 0.86, *p* = 0.004, respectively). The differences between the increase in EPCs CD34^+^,133^+^ and ProBNP levels also had a significant correlation (rho = −0.6, *p* < 0.001). CD34^+^,133^+^ EPCs showed a strong and positive correlation with parameters reflecting the severity of right heart failure and could be useful as a clinical tool for early identification of right ventricle maladaptation in pediatric patients with secondary PAH.

## A015 CLINICAL AND IMAGING RISK FACTORS FOR THE PERSISTENCE OF THROMBOEMBOLISM FOLLOWING ACUTE PULMONARY EMBOLISM

Weifang Liu, Sheng Xie, Tian Liang, Feiyan Chang, Min Liu, Zhenguo Zhai

*Civil Aviation General Hospital, Beijing 100123, China; Department of Radiology, Department of Pulmonary and Critical Care Medicine, China‐Japan Friendship Hospital, Beijing 100029, China*

The aim was to explore the risk factors of chronic persistence of thromboembolism after acute pulmonary embolism. Cases with new‐onset acute pulmonary embolism from November 2016 to November 2019 were analyzed retrospectively. The clinical characteristics and the serological examination results and treatment strategies of acute pulmonary embolism patients were obtained through the medical record system. Imaging parameters on computed tomography pulmonary angiography images at the onset of acute pulmonary embolism were measured and counted. According to the presence of residual embolus after 3 months of regular treatment for acute pulmonary embolism, patients were classified into the chronic pulmonary thromboembolism (CPTE) group or the non‐CPTE group. All data were compared between the CPTE group and the non‐CPTE group. Furthermore, logistic regression analysis was used to investigate the risk factors for the progression of acute pulmonary embolism to CPTE. A total of 77 cases were included in the study. There were 43 cases (55.84%) in the CPTE group and 34 cases in the non‐CPTE group (44.16%). The results of univariate analysis showed that there were statistically significant differences between the two groups in risk stratification (*p* = 0.005), protein S activity (*p* = 0.018), the ratio of the sum of residual segmental pulmonary artery diameter to the main pulmonary artery diameter (Sd/MPAd; *p* = 0.039), Mastora score (*p* < 0.001) and embolus location (*p* < 0.001). However, there were no statistically significant differences between the two groups in treatment options (*p* = 0.381). According to multivariate logistic regression analysis, protein S activity < 55%, Sd/MPAd ≥ 1.97 and the embolus being located in the central pulmonary artery were independent risk factors for chronic persistence of thromboembolism following acute pulmonary embolism. In concludion, the protein S activity and Sd/MPAd on computed tomography pulmonary angiography at the onset of acute pulmonary embolism may suggest the progression of acute pulmonary embolism to CPTE.

## A016 A LUNG GRAPH‐BASED MACHINE LEARNING IN ASSESSMENT OF ACUTE PULMONARY EMBOLISM ON THE NONCONTRAST COMPUTED TOMOGRAPHY

Mei Deng, Min Liu

*China–Japan Friendship Hospital, Chinese Academy of Medical Sciences & Peking Union Medical College, Beijing, China*

The aim was to build and evaluate a holistic lung graph‐based machine learning model (HLG‐ML) on non‐contrast computed tomography (NC‐CT) in diagnosis of acute pulmonary embolism (APE). This study included retrospectively 178 cases who underwent NC‐CT and computed tomography pulmonary angiogram. One hundred an thirty‐three cases (75% cases) and 45 cases (25% cases) were assigned randomly into the training group and testing group, respectively. A holistic lung graph was developed with a pulmonary radiomics descriptor derived from NC‐CT images. The approach extracted local radiomics features and encoded these local features into a radiomics descriptor, as a characterization of global radiomics feature distribution. Then eight machine learning models were trained and compared based on the descriptor. The diagnostic performance of the HLG‐ML model was compared with three radiologists. Gradient boosting decision trees gained the best classification performance on the training group. In the testing group, the proposed HLG‐ML showed better diagnostic ability, with area under the curve of 0.810 [95% confidence interval (CI): 0.682, 0.939] than three radiologists [0.573 (95% CI: 0.439, 0.707), 0.551 (95% CI: 0.414, 0.689) and 0.533 (95% CI: 0.384, 0.681)]. The feasibility of the proposed model might provide potential in the diagnosis and assessment of APE using NC‐CT.

## A017 DELETION OF INDUCIBLE NITRIC OXIDE SYNTHASE IN *ACTA2* ^+^ CELLS REVERSES CIGARETTE SMOKE‐INDUCED EMPHYSEMA

CHENG‐Yu Wu, Marija Gredic, Stefan Hadzic, Siddartha Doswada, Arun Lingampally, Werner Seeger, Friedrich Grimminger, Hossein A Ghofrani, Ralph T Schermuly, Saverio Bellusci, Simone Kraut, Norbert Weissmann

*Cardio‐Pulmonary Institute (CPI), Universities of Giessen and Marburg Lung Center (UGMLC), Member of the German Center for Lung Research (DZL), Justus‐Liebig‐University, Giessen, Germany*

Chronic obstructive pulmonary disease (COPD) is a global health problem and high socioeconomic burden. Besides airway disease and pulmonary emphysema, COPD patients may suffer from pulmonary hypertension (PH). A major cause of COPD development is cigarette smoking and air pollution. Previous data obtained from a mouse model of chronic exposure to cigarette smoke (CS) revealed that alterations in the pulmonary vasculature precede development of emphysema. Such data indicate a possible interaction between the pulmonary vasculature and the alveolar compartment during CS‐induced emphysema and PH development. The inducible nitric oxide synthase (iNOS) was identified as a key enzyme responsible for PH and emphysema development upon CS exposure in mice. Deletion of iNOS in non‐bone marrow‐derived cells protected mice from CS‐induced emphysema. Inhibition of iNOS in mice after chronic CS exposure led to alveolar regeneration and reversed remodeling of the pulmonary vasculature. Furthermore, we demonstrated significant iNOS upregulation in pulmonary vessels of CS‐exposed mice and human COPD lungs. However, the contribution of increased iNOS expression in lung vessels to CS‐induced emphysema and PH development remains unresolved. Against this background, we here investigated the role of iNOS in smooth muscle cells (SMCs) during CS‐induced emphysema and PH development. We used transgenic animals in which *iNos* was deleted in *Acta2* ^+^ cells. The animals were then exposed to CS for 3 or 8 months. Furthermore, we analyzed the therapeutic potential of *iNos* deletion in *Acta2* ^+^ cells, induced after establishment of emphysema and PH (after 8 months of CS exposure). Our data revealed that *iNos* deletion in *Acta2* ^+^ cells did not interfere with CS‐induced PH development. However, *Acta2* ^+^ cell‐specific *iNos* knockout delayed, but did not completely prevent CS‐induced emphysema. Most interestingly, fully established emphysema was reversed 3 months after CS cessation when *iNos* was deleted in *Acta2* ^+^ cells. Further investigations are needed to delineate which *Acta2* ^+^ cell population might contribute to lung regeneration and decipher the underlying molecular mechanisms.

## A018 MOLECULAR BASIS OF BMPRII: BMP10 INTERACTIONS AND ITS IMPLICATION IN PULMONARY ARTERIAL HYPERTENSION

Jingxu Guo, Bin Liu, Midory Thorikay, Richard M Salmon, Randy J Read, Peter ten Dijke, Nicholas W Morrell, Wei Li

*Department of Medicine, University of Cambridge School of Clinical Medicine, Cambridge, CB2 0QQ, UK; Cambridge Institute for Medical Research, Cambridge CB2 0XY, UK; Department of Cell and Chemical Biology and Oncode Institute, Leiden University Medical Centre, The Netherlands*

Heterozygous mutations in *BMPR2* [bone morphogenetic protein (BMP) receptor type II] are the major genetic cause for pulmonary arterial hypertension (PAH). BMPRII is a receptor for >15 BMP ligands, but it is not well understood why *BMPR2* mutations cause lung‐specific pathology. Around 668 *BMPR2* mutations have been identified to date, most of which are nonsense mutations resulting in haploinsufficiency. Although some missense mutations involving cysteine substitutions cause protein misfolding and a defect in trafficking, the pathogenic effects of non‐cysteine mutations are not fully understood. *BMPR2* is highly expressed in lung vascular endothelial cells. In complex with activin receptorlike kinase 1 (ALK1), it mediates the signalling from the circulating BMP9 and BMP10 ligands, maintaining the vascular quiescence. Interestingly, mutations in *ALK1*, *GDF2* (encoding BMP9) and *BMP10* have also been identified in PAH patients, suggesting a crucial role of this pathway in the pathogenesis of PAH. To understand the molecular interactions underpinning the BMPRII signalling complexes, we solved crystal structures of ALK1:BMP10 and BMP10:BMPRII binary receptor complexes and ALK1:BMP10:BMPRII ternary signalling complexes. We provide BMPRII‐specific interactions and those interactions that are important for BMPRII‐mediated signalling activity. The interactions between BMPRII and BMP10 are highly dynamic, explaining why BMPRII is a low‐affinity receptor. Our data suggest that forming and stabilizing BMPRII signalling complex require high concentrations of BMPRII at the cell surface; therefore, most of its activity will be in tissues with very high *BMPR2* expression, such as the lung vascular endothelial cells. Hence, *BMPR2* mutations that cause haploinsufficiency will have the most detrimental impact on the lung vasculature.

## A019 INOS–MAP KINASE AXIS IS RESPONSIBLE FOR INTERPLAY BETWEEN MACROPHAGES AND SMOOTH MUSCLE CELLS IN DEVELOPMENT OF TOBACCO SMOKE‐INDUCED PULMONARY VASCULAR DISEASE

Marija Gredic, Cheng‐Yu Wu, Stefan Hadzic, Oleg Pak, Rajkumar Savai, Andreas Weigert, Andreas Guenther, Ralf P Brandes, Ralph T Schermuly, Friedrich Grimminger, Werner Seeger, Natascha Sommer, Simone Kraut and Norbert Weissmann

*Cardio‐Pulmonary Institute (CPI), Universities of Giessen and Marburg Lung Center (UGMLC), Member of the German Center for Lung Research (DZL), Justus‐Liebig‐University (JLU), Giessen, Germany, Max Planck Institute for Heart and Lung Research, Member of the DZL, Bad Nauheim, Germany, Institute of Biochemistry I, Faculty of Medicine, Goethe‐University Frankfurt, Frankfurt, Germany, European IPF Registry & Biobank (eurIPFreg), Giessen, Germany; Agaplesion Evangelisches Krankenhaus Mittelhessen, Giessen, Germany, Institut für Kardiovaskuläre Physiologie, Fachbereich Medizin der Goethe‐Universität, Theodor‐Stern Kai 7, 60590 Frankfurt am Main, Germany*

Patients suffering from chronic obstructive pulmonary disease (COPD) often develop pulmonary hypertension (PH). Importantly, occurrence of even mild PH increases the risk of exacerbations and affects the patient's survival. We previously identified inducible nitric oxide synthase (iNOS) as an essential enzyme for pathogenesis and reversal of tobacco smoke‐induced PH and emphysema in mice. Moreover, our data revealed a causative role between deregulated iNOS activity in bone marrow‐derived cells and smoke‐induced pulmonary vascular alterations. However, which bone marrow‐derived cell type is responsible and the respective mechanism remained unresolved. To address this issue, we exposed myeloid cell type‐specific iNOS knockout mice to smoke and assessed the development of PH and emphysema in vivo. In addition, we performed in vitro investigations of the underlying signaling mechanisms in co‐cultures of bone marrow‐derived macrophages and pulmonary artery smooth muscle cells (PASMCs). Deletion of iNOS in myeloid cells completely prevented development of smoke‐induced PH in mice and ameliorated accumulation of ‘M2‐like’ lung interstitial macrophages. *In vitro* investigations revealed that smoke exposure of M2 macrophages resulted in augmented proliferation and migration of adjacent PASMCs. Importantly, observed effects were dependent on the presence of iNOS in macrophages and activation of extracellular signal‐regulated kinase (ERK) in PASMCs. Interestingly, iNOS activity in macrophages but not the smoke exposure of those cells affected the phosphorylation of p38 kinase in cocultured PASMCs. However, we found that activation of both ERK and p38 was increased in the lungs of smoke‐exposed wild‐type mice and in the medial layer of remodeled pulmonary vessels in COPD patients. Overall, our findings suggested that iNOS activity in myeloid cells guides the development of tobacco smoke‐induced PH. Furthermore, we demonstrated that the interplay with macrophages involves the activation of ERK and p38 in PASMCs. This may finally cause dysregulated PASMC proliferation and the occurrence of pulmonary vascular remodeling.

## A020 THE IMPORTANCE OF EARLY TREATMENT WITH INHALED TREPROSTINIL IN PATIENTS WITH PULMONARY HYPERTENSION ASSOCIATED WITH INTERSTITIAL LUNG DISEASE: A POST HOC ANALYSIS OF THE INCREASE OPEN‐LABEL EXTENSION

SD Nathan, VF Tapson, D Levine, F Rischard, S Cassady, HM DuBrock, K El‐Kersh, J Tarver III, P Smith, CQ Deng, E Shen, AB Waxman

*Inova Fairfax Hospital; Cedars‐Sinai Medical Center; Inari Medical; University of Texas Health Science Center at San Antonio, TX; University of Arizona College of Medicine, Tucson, AZ; University of Maryland School of Medicine, Baltimore, MD; Mayo Clinic, Rochester, MN; University of Nebraska Medical Center, Omaha, NE; AdventHealth Orlando; United Therapeutics; Brigham and Women's Hospital, Boston, MA, USA*

Inhaled treprostinil improved 6‐min walk distance (6MWD) in the INCREASE study, a 16‐week randomized, placebo‐controlled trial in patients with pulmonary hypertension attributable to interstitial lung disease (PH‐ILD), leading to the US Food and Drug Administration approving inhaled treprostinil as the first and only treatment for PH‐ILD. Patients completing INCREASE were eligible for the open‐label extension (OLE), in which all patients received inhaled treprostinil. This post‐hoc analysis of the OLE assesses the long‐term impact of delaying treatment with inhaled treprostinil in patients with less severe hemodynamic impairment.

Patients assigned placebo in the randomized portion of INCREASE were considered to have a treatment delay for this analysis. Change in 6MWD over 52 weeks was evaluated in OLE patients with baseline pulmonary vascular resistance (PVR) and mean pulmonary artery pressure (mPAP) below median values (<5.275 WU and <35 mmHg, respectively). Observed values were used, with no imputation. Week 0 represented the start of inhaled treprostinil therapy. The analysis was repeated using median N‐terminal pro B‐type natriuretic peptide (NT‐proBNP; 528 pg/mL) at initiation of inhaled treprostinil as a surrogate for hemodynamic status. Patients with PVR < 5.275 WU with a 16‐week treatment delay had a 6MWD change at week 48 of −19.3 m compared with +6.4 m at week 52 in those without a treatment delay. There were similar findings when evaluating long‐term 6MWD changes in patients with mPAP < 35 mmHg and NT‐proBNP < 528 pg/mL. In the INCREASE OLE, a 16‐week treatment delay in patients with less severe pulmonary vascular disease, based on PVR and mPAP, resulted in a prolonged decline and lack of improvement in 6MWD. Over ~1 year, patients previously assigned placebo with milder hemodynamics never ‘catch up’ to those originally assigned to treatment. When stratifying patients by NT‐proBNP, 6MWD results confirm PVR and mPAP findings. This analysis emphasizes the importance of early screening, diagnosis and treatment of PH in patients with ILD.

## A021 3D‐PRINTED ARTERIES FROM CHRONIC THROMBOEMBOLIC PULMONARY HYPERTENSION PATIENTS: A POTENTIAL TOOL FOR DISEASE MODELING?

Salina Nicoleau, Deepa Gopalan, Beata Wojciak‐Stothard

*National Heart and Lung Institute, Imperial College London, London; Hammersmith Hospital, Imperial College Healthcare NHS Trust, London, UK*

Chronic thromboembolic pulmonary hypertension (CTEPH) is a severe lung condition resulting from non‐resolving pulmonary emboli, which cause vascular remodelling and elevated pulmonary arterial pressure. Vascular homeostasis is largely orchestrated by the endothelium, which responds to mechanical stimuli exerted by blood flow. CTEPH patients show abnormal pulmonary vascular architecture and disturbed blood flow patterns, but the role of these changes in the pathobiology of the disease is not fully understood. The aim was to produce patient‐specific three‐dimensional (3D) models of pulmonary arterial stenosis to facilitate studies on the effect of blood flow on pulmonary endothelial dysfunction in CTEPH. Computed tomography (CT) scans of CTEPH lungs were used to recreate geometries of healthy and diseased 3D pulmonary arteries *in silico*. Fully circular and semi‐circular models displaying varying degrees of stenosis seen in CTEPH lungs were designed and produced using soft lithography and 3D printing. Blood flow within these structures was analysed using computational fluid dynamics (CFD). The models were seeded with human pulmonary artery endothelial cells (HPAECs) and perfused with xanthan gum‐based blood‐mimicking fluid. Gas‐permeable and optically transparent 3D models of CTEPH stenotic vascular channels were bioprinted using hydrogel and lined with HPAECs. The CFD analyses revealed that the cells were subjected to physiologically relevant ranges of pressures, flow patterns and wall shear stress, with 60–80% stenosis producing non‐laminar, disturbed flow. Polydimethylsiloxane (PDMS) semi‐circular channels replicating flow parameters and patterns seen in fully circular vessels were produced to facilitate live confocal imaging. Polyethylene (glycol) diacrylate (PEGDA) hydrogel was chosen as a preferred material for 3D printing of vascular channels. We have optimized the production of perfusable, patient‐specific 3D models of vascular networks, which might facilitate disease modelling and personalized medicine applications.

## A022 CONCURRENT ORAL ADMINISTRATION OF SILDENAFIL AND HOMING PEPTIDE IMPROVES PULMONARY HEMODYNAMICS IN RATS WITH EMPHYSEMA INDUCED BY SU5416/HYPOXIA

Yosuke Wada, Norihiko Goto, Yusuke Suzuki, Yoshiaki Kitaguchi, Masanori Yasuo, Masayuki Hanaoka

*First Department of Internal Medicine; Departments of Clinical Laboratory Sciences, Shinshu University School of Health Sciences, Matsumoto, Nagano; First Department of Internal Medicine, Shinshu University School of Medicine, Matsumoto, Japan*

Vascular endothelial growth factor (VEGF) is regulated by hypoxia stimulation in a variety of tissues. Chronic treatment of rats with the VEGF receptor blocker SU5416 leads to enlargement of air spaces in lung tissues, indicative of the formation of emphysema. In addition, rats exposed to SU5416/hypoxia develop pulmonary hypertension. Based on this evidence, the present study investigated whether the chronic hemodynamic deterioration occurring in rats with emphysema was induced by SU5416/hypoxia and aimed to verify the effects of combined administration of sildenafil and homing peptide (CAR) on the hemodynamic deterioration in the SU5416/hypoxia‐treated rats. The animal protocol was approved by the Animal Care and Use Committee of the Shinshu University. Male Sprague–Dawley rats were injected SU5416 on days 1, 8 and 15, and simultaneously exposed to hypoxia for 6 weeks. Sildenafil and CAR were administered orally to rats every day for 5 days immediately before dissection. Pulmonary hemodynamics were measured on day 42 of the experiment. The lung tissues were sampled after experimental scarifice for histological examination and further biomolecular study. The air space size was quantified by the mean linear intercept (MLI). The MLI was significantly higher in the SU5416/hypoxia‐treated rats than the control rats, suggesting enlargement of air spaces in the SU5416/hypoxia rats. In addition, the mean pulmonary arterial pressure (mPAP) was significantly higher in the rats treated with SU5416/hypoxia than the control rats. However, this increase of mPAP in the SU5416/hypoxia‐treated rats was significantly inhibited by the concurrent treatment with sildenafil and CAR, with an effective improvement of the pulmonary hemodynamics. In western blot analysis, the concurrent treatment with sildenafil and CAR significantly inhibited the increase of cleaved caspase‐3 in the SU5416/hypoxia‐treated rats. The concurrent oral administration of sildenafil and CAR improves pulmonary hemodynamics in rats exposed simultaneously to a VEGF receptor inhibitor and hypoxia.

## A023 ANALYSIS OF THE COMPERA 2.0 RISK ASSESSMENT TOOL IN THE REPLACE STUDY

RL Benza, G Simonneau, HA Ghofrani, PA Corris, S Rosenkranz, D Langleben, C Meier, C Rahner, MM Hoeper

*Division of Cardiovascular Diseases, Ohio State University, Columbus, OH, USA; Assistance Publique–Hôpitaux de Paris, Service de Pneumologie, Hôpital Bicêtre, Université Paris‐Saclay, Laboratoire d'Excellence en Recherche sur le Médicament et Innovation Thérapeutique, and Inserm U999, Le Kremlin‐Bicêtre, France; University of Giessen and Marburg Lung Center, member of the German Center for Lung Research (DZL), Giessen, Germany; Department of Pneumology, Kerckhoff Clinic, Bad Nauheim, Germany; Department of Medicine, Imperial College London, London, UK; Translational and Clinical Research Institute, Newcastle University, Newcastle upon Tyne, UK; Clinic III for Internal Medicine (Cardiology), Cologne Cardiovascular Research Center (CCRC), and Center for Molecular Medicine Cologne (CMMC), University of Cologne, Cologne, Germany; Center for Pulmonary Vascular Disease and Lady Davis Institute, Jewish General Hospital, McGill University, Montreal, QC, Canada; Global Medical Affairs, Bayer AG, Berlin, Germany; Chrestos Concept GmbH & Co. KG, Essen, Germany; Clinic for Respiratory Medicine, Hannover Medical School, member of the German Center for Lung Research (DZL), Hannover, Germany*

In REPLACE, patients with pulmonary arterial hypertension (PAH) at intermediate risk despite phosphodiesterase‐5 inhibitor (PDE5i) treatment achieved clinical improvement when switched to riociguat compared with those who continued PDE5i. Significant improvements in risk status with riociguat versus PDE5i maintenance were seen using the three‐strata COMPERA risk assessment tool. COMPERA 2.0 is a new four‐strata tool designed to be more sensitive to prognostically relevant changes in risk. We applied COMPERA 2.0 to REPLACE post hoc to investigate potential differences in outcomes between intermediate–low‐risk and intermediate–high‐risk patients. COMPERA 2.0 scores at baseline and week 24, and change from baseline, were calculated. Response (achievement of the REPLACE primary endpoint at week 24) and clinical worsening were assessed in patients categorized as intermediate–low and intermediate–high risk by COMPERA 2.0 at baseline. At baseline in the riociguat (*n* = 111) and PDE5i (*n* = 113) groups, 60% and 55% of patients were intermediate–low risk and 40% and 45% were intermediate–high risk, respectively. Improvement in COMPERA 2.0 from baseline to week 24 was significantly higher with riociguat versus PDE5i [45% versus 25%, respectively; mean difference (95% confidence interval) 0.23 (0.05 to 0.41); *p* = 0.0059]. At week 24, 28% of riociguat‐treated patients and 12% of PDE5i‐treated patients improved to low‐risk status. A similar percentage of intermediate–low‐risk patients (riociguat, 39%; PDE5i, 19%) and intermediate–high‐risk patients (riociguat, 43%; PDE5i, 22%) achieved the primary endpoint at week 24. All intermediate–low‐risk and intermediate–high‐risk responders showed significant improvements in COMPERA 2.0 at week 24 versus baseline and versus non‐responders. All patients who died or experienced clinical worsening were intermediate–high risk at baseline. Patients with PAH at intermediate–low risk and intermediate–high risk by COMPERA 2.0 at baseline achieved similar clinical improvement when switching to riociguat from PDE5i therapy in REPLACE. COMPERA 2.0 provides a risk assessment approach for better differentiating the large group of patients at intermediate risk. The REPLACE study was co‐funded by Bayer AG (Berlin, Germany) and Merck Sharp & Dohme Corp., a subsidiary of Merck & Co., Inc. (Kenilworth, NJ, USA). R.L.B. reports grants from Actelion, Bellerophon, Bayer AG and EIGER.

## A024 ANALYSIS OF RISK ASSESSMENT TOOLS IN THE REPLACE STUDY

RL Benza, G Simonneau, HA Ghofrani, PA Corris, S Rosenkranz, D Langleben, C Meier, C Rahner, MM Hoeper

*Division of Cardiovascular Diseases, Ohio State University, Columbus, Ohio, USA; Assistance Publique–Hôpitaux de Paris, Service de Pneumologie, Hôpital Bicêtre, Université Paris‐Saclay, Laboratoire d'Excellence en Recherche sur le Médicament et Innovation Thérapeutique, and Inserm U999, Le Kremlin‐Bicêtre, France; University of Giessen and Marburg Lung Center, member of the German Center for Lung Research (DZL), Giessen, Germany; Department of Pneumology, Kerckhoff Clinic, Bad Nauheim, Germany; Department of Medicine, Imperial College London, London, UK; Translational and Clinical Research Institute, Newcastle University, Newcastle upon Tyne, UK; Clinic III for Internal Medicine (Cardiology), Cologne Cardiovascular Research Center (CCRC) and Center for Molecular Medicine Cologne (CMMC), University of Cologne, Cologne, Germany; Center for Pulmonary Vascular Disease and Lady Davis Institute, Jewish General Hospital, McGill University, Montreal, Quebec, Canada; Global Medical Affairs, Bayer AG, Berlin, Germany; Chrestos Concept GmbH & Co. KG, Essen, Germany; Clinic for Respiratory Medicine, Hannover Medical School, member of the German Center for Lung Research (DZL), Hannover, Germany*

The REPLACE study showed that PAH patients treated with phosphodiesterase‐5 inhibitors (PDE5i) still at intermediate risk could achieve clinical improvement when switched to riociguat. Significant improvements in risk status with riociguat versus PDE5i were seen using the COMPERA and Noninvasive French Pulmonary Hypertension Network (FPHN) risk scores, but not the REVEAL risk score (RRS). We investigated possible reasons for this difference. Achievement of the REPLACE primary endpoint of clinical improvement at week 24 was assessed in patients stratified into low‐ and intermediate‐/high‐risk status by RRS at baseline. Change in RRS from baseline, clinical worsening and deaths were also assessed in responders (patients achieving the endpoint) versus non‐responders. Individual components of RRS, COMPERA and FPHN were calculated for both groups at baseline and week 24. At baseline, more than half of the patients in each group (riociguat, 53%; PDE5i, 57%) were low risk by their RRS. Nonetheless, 41% of low‐risk riociguat patients and 17% of low‐risk PDE5i patients were responders, similar to intermediate‐/high‐risk patients (40% vs. 24%). Low‐risk responders also experienced significant improvements in RRS versus baseline and versus low‐risk non‐responders. Of 11 clinical worsening events, two were in low‐risk PDE5i patients. No deaths occurred in low‐risk patients. All risk‐assessment tools showed similar trends in shared components; however, at week 24, more patients on riociguat (46%) versus PDE5i (31%) had 1–2 points added to their RRS owing to the systolic blood pressure aspect of the vital signs component. When RRS was recalculated excluding vital signs, the difference between the riociguat and PDE5i groups was significant, as with the COMPERA and FPHN risk scores. The inclusion of vital signs in the RRS can explain the different risk‐assessment results in REPLACE. Patients considered low risk by RRS are still capable of achieving clinical improvement if switched to riociguat from PDE5i. The REPLACE study was co‐funded by Bayer AG (Berlin, Germany) and Merck Sharp & Dohme Corp., a subsidiary of Merck & Co., Inc. (Kenilworth, NJ, USA). R.L.B. reports grants from Actelion, Bellerophon, Bayer AG and EIGER.

## A025 LONG‐TERM EFFECTS OF PULMONARY ENDARTERECTOMY ON RIGHT VENTRICULAR REMODELING IN CHRONIC THROMBOEMBOLIC PULMONARY HYPERTENSION

Azar Kianzad, Natalia J Braams, Jessie van Wezenbeek, Jeroen N Wessels, Samara MA Jansen, Anco Boonstra, Esther J Nossent, J Tim Marcus, Ahmed Bayoumi, Clarissa Becher, Marie‐Jose Goumans, Anton Vonk Noordegraaf, Frances S de Man, Harm Jan Bogaard, & Lilian J Meijboom

*Department of Pulmonary Medicine; Department of Radiology and Nuclear Medicine; Amsterdam Cardiovascular Sciences, Amsterdam UMC, Vrije Universiteit Amsterdam, Amsterdam, The Netherlands; Department of Molecular Cell Biology, Leiden University Medical Centre, Leiden, The Netherlands*

Surgical removal of thromboembolic material by pulmonary endarterectomy (PEA) leads to improvement of right ventricular (RV) function in the majority of chronic thromboembolic pulmonary hypertension (CTEPH) patients. However, RV mass does not always normalize post‐PEA. Whether this is the result of extracellular matrix expansion (diffuse interstitial fibrosis) or cellular hypertrophy is unknown. We prospectively included 25 CTEPH patients treated with PEA (mean pulmonary artery pressure post‐PEA 23 ± 9 mmHg). Structured follow‐up measurements were performed before and 6‐ and 18‐months post‐PEA. To study changes in RV remodeling, extracellular volume fraction (ECV) of the RV free wall (RVFW) by cardiac MRI was measured and was used to divide the myocardium into cellular and matrix volume. With single beat pressure–volume loop analysis, load‐independent RV diastolic stiffness (Eed) was assessed. Circulating collagen biomarkers were analyzed with an enzyme‐linked immunosorbent assay to determine the contribution of collagen metabolism. Right ventricular mass decreased significantly but did not normalize 6 months post‐PEA (from 43 ± 15 to 27 ± 11 g/m^2^, *p* < 0.001). The ECV in the RVFW increased post‐PEA (from 31 ± 4 to 34 ± 4%, *p* = 0.013) and was the result of a larger reduction in cellular volume relative to matrix volume (−38% vs. −30%, *P*‐interaction = 0.0013). The Eed significantly improved and normalized after PEA, but Eed corrected for relative wall thickness did not reduce post‐PEA. Levels of matrix metalloproteinase 1 [5 (1–11) to 4 (1–9) ng/mL, *p* = 0.3], tissue inhibitor of metalloproteinases 1 [247 (196–313) to 221 (145–283) ng/mL, *p* = 0.036] and transforming growth factor‐β [9 (5–16) to 6 (3–11) ng/mL, *p* = 0.07] were elevated at baseline and remained elevated post‐PEA. No further improvements were observed between 6 and 18 months after PEA. Although cellular hypertrophy regresses post‐PEA, there is a relative increase in extracellular volume, indicating that extracellular matrix regression is not complete in the RV. This was accompanied by elevated levels of collagen biomarkers suggestive of an active collagen turnover.

## A026 ACUTE VASOREACTIVITY IN GROUP 3 PULMONARY HYPERTENSION AND IMPLICATIONS FOR INHALED TREPROSTINIL RESPONSE

Eileen M Harder, David M Systrom, Aaron B Waxman

*Division of Pulmonary and Critical Care, Brigham and Women's Hospital, Boston, MA, USA*

Although originally intended to identify calcium channel blocker responders, vasoreactivity is now understood to provide insight into disease pathophysiology and outcomes. We routinely assess vasodilator response in all precapillary PH. Given that both remodeling and inhaled vasodilator effects occur in the distal vasculature, we sought to examine acute vasoreactivity and its relationship with long‐term inhaled treprostinil (iTre) response. Treatment‐naïve croup 1 and 3 patients with acute vasoreactivity were identified. Testing entailed sequential hemodynamics breathing room air, oxygen and nitric oxide (iNO). Distensibility (diameter change per millmeter of mercury; vessel wall adaptation to increased flow) was calculated. The iTre response was assessed at 6 months. Clinical worsening was a composite of suboptimal response requiring treatment escalation/cessation; objective testing deterioration; cardiopulmonary hospitalization; lung transplantation ≥ 3 months; or death. Non‐responders experienced clinical worsening. One hundred and sixty‐two subjects were divided into group 1 (58) and group 3 PH cohorts (104; 37 chronic obstructive pulmonary disease, 67 fibrotic disease). Group 3 PH was associated with better baseline hemodynamic and pulmonary vascular stiffness (e.g. distensibility, vs. pulmonary arterial hypertension, 0.37 vs. 0.26%/mmHg, *p* < 0.001). In group 3 PH, hemodynamic improvements with iNO were larger [mean pulmonary artery pressure (mPAP) versus group 1, −11.1 versus −7.1%, *p* = 0.014] and more frequent [versus group 1; decrease ≥20% in mPAP and pulmonary vascular resistance (PVR), 25.0 versus 12.1%, *p* = 0.050]. The PVR response was similar. In 31 group 3 PH patients with PVR ≥ 4 WU, acute change in distensibility with sequential inhaled vasodilators differed with iTre response. Responders had larger relative distensibility increases with iNO (vs. non‐responders, 67.7 vs. 17.2%, *p* = 0.014), which persisted in multivariate analysis. Conversely, non‐responders had larger distensibility improvements with O_2_ alone (vs. responders, 28.0 vs. −5.6%, *p* = 0.010). Vasoreactivity is common and may guide therapy in group 3 PH. The response of the distal vasculature to specific inhaled vasodilators accurately predicted long‐term iTre response, underscoring the variable contributions of hypoxic and pathophysiologic vasculopathy to group 3 PH and their disparate implications for treatment.

## A027 THE EFFECTS OF CIGARETTE SMOKE ARE REVERSED BY SOLUBLE GUANYLATE CYCLASE STIMULATION IN VITRO THROUGH NORMALIZATION OF THE C‐JUN N‐TERMINAL KINASE (JNK) PATHWAY IN PULMONARY ARTERY SMOOTH MUSCLE CELLS

Adelaida Bosacoma, Isabel Blanco, Olga Tura‐Ceide, Joan A Barberà, Victor I Peinado

*Department of Pulmonary Medicine, Hospital Clínic‐Institut d'Investigacions Biomèdiques August Pi i Sunyer (IDIBAPS); University of Barcelona; 08036, Barcelona, Spain; Biomedical Research Networking Centre on Respiratory Diseases (CIBERES), 28029, Madrid, Spain; Department of Pulmonary Medicine, Dr. Josep Trueta University Hospital de Girona, Santa Caterina Hospital de Salt and the Girona Biomedical Research Institut (IDIBGI), 17190, Girona, Spain; Department of Experimental Pathology, Institut d'Investigacions Biomèdiques de Barcelona, Consejo Superior de Investigaciones Cientificas (IIBB‐CSIC) Barcelona, Spain*

In a guinea‐pig model of chronic obstructive pulmonary disease, we identified that emphysema and/or pulmonary hypertension (PH) induced by cigarette smoke (CS) was related to the alteration of genes of pathways such as inflammation, apoptosis and cell proliferation. Treatment with a soluble guanylate cyclase (sGC) stimulator induced the normalization of ~50% of altered genes, especially those related to the mitogen‐activated protein kinase pathway, while improving PH values and reversing emphysema. The aim of the present study was to evaluate in vitro the effects of CS on the JNK pathway in endothelial cells (HPAECs) and smooth muscle cells (PASMCs) of human pulmonary arteries, in addition to the effects of the sGC stimulator BAY 63‐2521. Commercially available human lung cells (HPAECs and PASMCs) were cultured with CS extract (CSE; diluted range 0–1/5) and BAY 63‐2521 (100 μM) to analyse cell viability and JNK‐related gene expression by qRT‐PCR. The results showed that growth rate diminished more in HPAECs than in PASMCs after addition of CSE. Besides, CSE (1/5) significantly increased the expression of *JUN* and *FOS* in both HPAECs and in PASMCs, in addition to the genes related to apoptosis *CASP3* and *P21*. Remarkably, in PASMCs cultured with CSE and treated with BAY 63‐2521, but not in HPAECs, the expression level of these genes was normalized to controls. In conclusion, these results highlight the importance of PASMCs in the normalization of JNK pathways by sGC stimulators and suggest a prominent role of these cells in the control of PH and CS‐induced emphysema.

## A028 INHALED ILOPROST IMPROVES RIGHT VENTRICULAR LOAD‐INDEPENDENT CONTRACTILITY IN PULMONARY HYPERTENSION

Nils Kremer, Manuel Richter, Henning Gall, Hossein Ardeschir Ghofrani, Robert Naeije, Werner Seeger, Khodr Tello

*Department of Internal Medicine, Justus‐Liebig‐University Giessen, Universities of Giessen and Marburg Lung Center (UGMLC), Member of the German Center for Lung Research (DZL), Giessen, Germany; Department of Pathophysiology, Free University of Brussels Campus de la Plaine, Brussels, Belgium; Department of Internal Medicine, Justus‐Liebig‐University Giessen, Universities of Giessen and Marburg Lung Center (UGMLC), Member of the German Center for Lung Research (DZL), Giessen, Germany*

The adaptation of the right ventricular contractility to its afterload (RV–PA coupling), best measured by the ratio of end‐systolic elastance (Ees) and arterial elastance (Ea), is a key determinant for prognosis and symptomatology in pulmonary hypertension (PH). It is not completely understood whether approved PH therapies acutely affect RV–PA coupling. We compared the acute effects of inhaled iloprost (2.5 μg) and drugs targeting the NO–cGMP pathway (inhaled NO, 10 ppm or riociguat, 1 mg) on RV contractility defined by load‐independent pressure–volume relationships in patients with pulmonary arterial hypertension (*n* = 19, 8 and 5, respectively). Right ventricular pressures and volumes were measured using high‐fidelity conductance catheters. Load‐independent contractility (Ees) and afterload (Ea) were determined using either the multibeat method with preload reduction via balloon occlusion of the inferior vena cava or, in patients who not consented to this procedure, with the Valsalva maneuver and supplementary single‐beat analysis. Values are reported as the mean ± SD or median and interquartile range. We observed a significant decrease of Ea in both iloprost [0.66 (0.41–0.86) versus 0.54 (0.34–0.75) mmHg/mL] and NO/riociguat [0.56 (0.48–1.33) versus 0.48 (0.36–1.11) mmHg/mL]. The Ees decreased with NO and riociguat [0.70 (0.51–1.04) versus 0.59 (0.45–0.74) mmHg/mL] closely to the same extend as Ea, whereas iloprost increased Ees significantly [0.56 (0.40–0.79) versus 0.63 (0.53–0.90) mmHg/mL]. Consequently, Ees/Ea was not affected by NO/riociguat but improved with iloprost (0.93 ± 0.44 vs. 1.46 ± 0.70). Both decreased RV volumes and increased RV ejection fraction. However, only iloprost increased stroke volume [79 (67–97) versus 85 (73–98) mL] and cardiac output [5.7 (4.4–6.9) versus 6.5 (5.0–7.6) L/min]. Of note, we observed no changes in the heart rate. Our results strongly suggest that inhaled iloprost acutely increases ejection fraction and cardiac output by a combination of decreased afterload and increased right ventricular contractility. In patients receiving NO or riociguat, the drug‐induced decrease in afterload was accompanied by a decrease in contractility, preserving RV–PA coupling.

## A029 NUTRITIONAL STATUS IN PULMONARY ARTERIAL HYPERTENSION

C T Kwant, F A L van der Horst, F S de Man, H J Bogaart, A Vonk Noordegraaf

*Department of Pulmonary Medicine, Amsterdam UMC, Vrije Universiteit Amsterdam, Amsterdam Cardiovascular Sciences, Amsterdam, The Netherlands; Department of Clinical Chemistry, Reinier Haga Medica Diagnostic Center, Delft, The Netherlands*

Nutritional deficiencies have been described in patients with pulmonary arterial hypertension (PAH), such as iron and vitamin D. However, an extensive description of vitamin and mineral status is lacking, and until now, there are no data on dietary intake in PAH‐ patients. In total, 37 patients were included (six males, 31 females; of 47.92 ± 16.25 years old). Blood analyses were carried out in a routine setting of an ISO15189:2012 accredited medical laboratory. Nutritional intake was analyzed using a food frequency questionnaire (HELIUS). Quality of life (QoL) was assessed by the SF‐36 questionnaire. We could confirm previously observed deficiencies of iron and vitamin D in our study population. In addition, we could observe a functional vitamin B_12_ deficiency in some of the patients (29%), which coincided with an increased expression of methylmalonic acid. Sixty per cent of the patients had a low vitamin K_1_ status (<0.8 nmol/L), from which less than half used vitamin K antagonists. Finally, 40% had selenium levels <100 μg/L, and we could observe an association with reduced QoL in these patients. The dietary intake of sugar was >25 g in 87% of the patients, and fluid intake was >1,500 mL in 78% of the patients. Sodium intake was <1,800 mg in the majority (56%) of the patients. Sugar and fluid intake were linearly related. Besides the known deficiencies in iron and vitamin D levels, we observed in a subset of patients signs of vitamin B_12_, vitamin K_1_ and selenium deficiencies. There is room for improving dietary intake. Future research will demonstrate the clinical importance and will reveal the effect of nutritional interventions.

## A030 SPECLE‐TRACKING ECHOCARDIOGRAPHY IN PATIENTS WITH IDIOPATHIC PULMONARY ARTERIAL HYPERTENSION

Yu A Botsiuk, O O Torbas, Yu M Sirenko

*SI National Scientific Center, Institute of Cardiology named after acad.M.D. Strazheska’ National Academy of Medical Science of Ukraine*

The aim was to evaluate the diagnostic possibilities of using speckle‐tracking echocardiography (ST‐Echo) in patients with idiopathic pulmonary arterial hypertension (IPAH) and to compare the results with a healthy population. The study included 27 patients with IPAH and nine people who were in the control group. Both groups were comparable by age and sex. All patients underwent general clinical studies, blood tests to determine the level of N‐terminal polypeptide of brain natriuretic hormone (NT‐proBNP), 6‐min walk test, transthoracic and ST‐Echo, cardio‐ankle vascular index (CAVI) and right heart catheterization (RHC) with a Swan‐Ganz catheter to determine central hemodynamic parameters. According to echocardiography, in patients with IPAH, TAPSE, FAC, RIMP and S′ of the right ventricle, right (RV GLS) and left ventricular global longitudinal strain (LV GLS) and right ventricular global longitudinal strain rate (RV GLSR) were significantly worse than in the control group. Using correlation analysis, it was found that RV GLS was most strongly correlated, among others, with the walked distance (*p* < 0.001) and blood oxygen saturation (*p* < 0.05) by the 6‐min walk test, NT‐proBNP (*p* < 0.001), systolic pulmonary artery pressure according to echocardiography (*p* < 0.001) and CAVI (*p* < 0.001). In contrast, the highest correlation with direct hemodynamic measurements was shown by two indicators: TAPSE, with cardiac index (*p* < 0.05), pulmonary vascular resistance (*p* < 0.05) and diastolic pulmonary artery pressure (*p* < 0.05); and RIMP, with diastolic pulmonary artery pressure (*p* < 0.001) and mean pulmonary artery pressure (*p* < 0.05). According to our results, we can conclude that a comprehensive assessment of right ventricular function using transthoracic and ST‐Echo allows a more individualized assessment of patients with IPAH. ST‐Echo can be used in PH reference centers for initial patient examination and follow‐up.

## A031 STUDY OF A NOVEL HYPOXIA SYSTEM TO EVALUATE ENDOTHELIAL CELL DYSFUNCTION IN CHRONIC THROMBOEMBOLIC PULMONARY HYPERTENSION

Ylenia Roger, Isaac Almendros, Esther Marhuenda, Adelaida Bosacoma, Anna Sardiné, Àngela Vea, Ana Ramírez, Víctor I Peinado, Isabel Blanco, Joan A Barberà, Olga Tura‐Ceide, Manuel Castellà, Josep Trueta

*Pneumology service, Hospital Clínic‐University of Barcelona, Institut d'Investigacions Biomèdiques August Pi i Sunyer (IDIBAPS), Barcelona; Centro de Investigación Biomédica en Red de Enfermedades Respiratorias (CIBERES), Barcelona; Unit of Biophysics and Bioengineering, Department of Physiological Sciences I, School of Medicine, University of Barcelona, Barcelona; Cardiovascular surgery service, Cardiovascular institute, Hospital Clínic‐University of Barcelona, Barcelona; Hospital Santa Caterina de Salt, Institut d'investigació biomèdica de Girona (IDIBGI), Girona, Spain*

Oxygen (O_2_) plays a key role in respiratory diseases, and hypoxia can be essential in the progression of diseases such as chronic thromboembolic pulmonary hypertension (CTEPH). The endurance of hypoxic conditions can contribute to a metabolic shift characterized by an abnormal cell proliferation, tissue hypertrophy and remodelling. The aim of the study was to evaluate the effect of chronic hypoxia on endothelial cells (ECs) derived from patients with CTEPH (EC‐CTEPH) compared with healthy controls and to identify potential differences when ECs are subjected to different O_2_ culture conditions. Both patient and control cells were cultured in different O_2_ conditions (1, 4, 13 and 21 O_2_) for 48 h using a novel cell culture system developed in our laboratory. qRT‐PCR for angiogenic and metabolic genes, supernatant (SN) analysis and migration assays were performed using EC‐CTEPH (*n* = 6). Healthy ECs were used as the control group (*n* = 6). Both cell populations reacted to hypoxia (1% O_2_) by upregulating hypoxic responsive genes such as *VEGF* or *NIP3*. Genes related to oxidative stress (*NOX4*, *SOD2*) were also significantly upregulated under hypoxia compared with normoxia in both groups. Genes related to the glycolytic pathway, such as *HK2* or *LDHA*, were only upregulated in control cells under 1% O_2_. These genes were not significantly upregulated in EC‐CTEPH. This was correlated with a lack in lactate production in EC‐CTEPH. Lactate production was increased in control cells under hypoxia, whereas it was not increased in EC‐CTEPH. Both cell populations responded to hypoxia (1% O_2_) by upregulating genes involved in different pathways. EC‐CTEPH present an altered metabolic reprogramming with a reduction in glycolytic gene expression under hypoxia compared with healthy controls.

## A032 CLINICAL CHARACTERISTICS, MANAGEMENT AND SURVIVAL OF PATIENTS WITH IDIOPATHIC PULMONARY ARTERIAL HYPERTENSION WITH CARDIOVASCULAR COMORBIDITIES: DATA FROM THE HELLENIC PULMONARY HYPERTENSION REGISTRY (HOPE)

Alexandra Arvanitaki, Elena Vrana, Maria Boutsikou, Anastasia Anthi, Aikaterini Avgeropoulou, Eftychia Demerouti, Alexandros Patrianakos, Panagiotis Karyofyllis, Ioanna Mitrouska, Sophia Anastasia Mouratoglou, Katerina K Naka, Stylianos E Orfanos, Evangelia Panagiotidou, Georgia Pitsiou, Spyridon Rammos, Ioannis Stanopoulos, Adina Thomaidi, Alexandra Frogoudaki, Afroditi Boutou, George Anastasiadis, Styliani Brili, Iraklis Tsangaris, Dimitrios Tsiapras, Vassilios Voudris, Athanasios Manginas, George Giannakoulas

*Cardiology Department, AHEPA University Hospital, Aristotle University of Thessaloniki; Cardiology Department, Mediterraneo Hospital, Glyfada, Athens; 1st Department of Critical Care and Pulmonary Hypertension Clinic, National & Kapodistrian University of Athens Medical School, Evaggelismos General Hospital, Athens; Sotiria Apostolopoulou, Cardiology – Pediatric Cardiology Department, Onassis Cardiac Surgery Center, Athens; Cardiology Department, Hippokration General Hospital, Athens; Cardiology – Pediatric Cardiology Department, Onassis Cardiac Surgery Center, Athens; Department of Thoracic Medicine, University Hospital of Heraklion, Heraklion, Crete; 2* ^*nd*^ *Department of Cardiology, University of Ioannina Medical School, University Hospital of Ioannina, Ioannina; Respiratory Failure Unit, ‘G. Papanikolaou’ Hospital, Exohi, Thessaloniki; Cardiology Department, Democritus University of Thrace, Alexandroupolis; Multidisciplinary Pulmonary Hypertension Center, Attikon University General Hospital, Athens; Cardiology Department, Laiko General Hospital, Athens, Greece*

Although younger female patients were diagnosed with idiopathic pulmonary arterial hypertension (IPAH) in 1980s, it is now frequently encountered in elderly patients with cardiovascular comorbidities (CVCs) associated with increased risk for left heart disease (left ventricular diastolic dysfunction). Present data until November 2019 regarding specific features and clinical outcomes of the IPAH population from the Hellenic Pulmonary Hypertension Registry (HOPE). Patients were divided into two groups based on the presence of ≥3 CVCs or <3 CVCs; arterial hypertension, diabetes mellitus, obesity, presence of coronary artery disease or atrial fibrillation. Overall, 77 patients with IPAH {55.1 [interquartile range (IQR) 24.1] years, 62.8% women} were analysed. Fifteen patients (19.2%) had ≥3 CVCs, and 25 (32%) were >65 years old. Patients with ≥3 CVCs were older [69.8 (IQR 17.8) versus 52.1 (IQR 23.3) years, *p* = 0.001], presented an almost equal female‐to‐male ratio (1.1/1), walked less in the 6‐min walk test [322 (IQR 197.8) versus 431 (IQR 128.3) m, *p* = 0.003] and had lower mean arterial pulmonary pressure [39 (IQR 19) versus 49 (IQR 16) mmHg, *p* = 0.011] and pulmonary vascular resistance [6.4 (4.2) versus 9.8 (5.2) Wood units, *p* = 0.008] at baseline than patients with fewer CVCs. Fewer patients with ≥3 CVCs received PAH‐specific treatment compared with patients with fewer comorbidities [*n* = 11 (73.3%) versus *n* = 58 (95.5%), *p* = 0.02]. During a median follow‐up period of 3.8 (IQR 2.7) years, 18 patients died (all‐cause mortality, 24.3%). Male sex, older age and worse risk status for 1‐year mortality were associated with worse survival prospects, and CVCs did not have a significant impact on survival. In this nationwide register‐based study, the epidemiology of IPAH involves older patients with CVCs, who seem to have less haemodynamic compromise but worse functional impairment and are treated less aggressively with PAH pharmacotherapy.

## A033 AUTOMATED CLOT BURDEN DETECTION AND ASSESSMENT FOR CHRONIC THROMBOEMBOLIC PULMONARY HYPERTENSION SEVERITY

Pietro Nardelli, Farbod N Rahaghi, Andetta R Hunsaker, Aaron B Waxman, Raúl San José Estépar

*Department of Radiology; Division of Pulmonary and Critical Care, Brigham and Women's Hospital, Harvard Medical School, Boston, MA, USA*

A loss of distal vasculature and proximal vessel dilation in patients with chronic thromboembolic pulmonary hypertension (CTEPH) has been demonstrated recently. However, the detection and quantification of micro‐clotting in small vessels remains a challenging task. The main purpose of this work was to develop an artificial intelligence (AI)‐based technique to detect and measure pulmonary micro‐blood clots inside the vascular tree automatically and define new markers of CTEPH. The micro‐clots detection and assessment followed two main steps. First, a two‐dimensional convolution neural network was trained on 7,500,000 synthetic vessel patches, resembling real vessels of different sizes and containing varying degrees of central and wall occlusions, to detect and regress clot percentage area. The network was validated by relative error (RE) between the automatic measurement of 200,000 synthetic patches (not used for training) and the ground truth of the geometric model. Then, we defined a marker of CTEPH (i.?e., clot score) as the 50th percentile of the clot area percentage histogram of the intraparenchymal vascular tree. T‐test analysis and logistic regression were used to explore our ability to identify subjects with and without CTEPH and investigate sensitivity and specificity. A total of 15 healthy subjects (no evidence of pulmonary or heart disease) and 18 patients diagnosed with CTEPH (based on hemodynamics and imaging characteristics) from Brigham and Women's Hospital for which we had complete information were considered (16 males and 17 females; mean age 51.8 ± 14.2 years). The mean RE on synthetic patches was 10.2 ± 3.2%. Using our clot score, a statistically significant difference (*p* < 0.001) was found between the two groups, and logistic regression analysis yielded an area under the receiver operating characteristic curve of 0.89. We have proposed an innovative technique for clot burden detection and assessment. Although more testing is required to validate the reliability and robustness of the method, this method paves the way to the definition of specific markers of CTEPH.

## A034 CHRONIC OBSTRUCTIVE PULMONARY DISEASE‐ASSOCIATED PULMONARY HYPERTENSION DETECTED BY NOVEL PULMONARY BLOOD VESSEL QUANTIFICATION SYSTEM: IMPACT ON CLINICAL CHARACTERISTICS AND OUTCOMES

Hector R Cajigas, William S Harmsen, Joana Costa, Patrick Muchmore, Benjamin R Lavon

*Division of Pulmonary and Critical Care Medicine, Mayo Clinic, Rochester, MN; Quantitative Health Research, Mayo Clinic, Rochester, MN, USA, Fluidda Inc., New York, NY, USA*

Pulmonary hypertension (PH) in patients with chronic obstructive pulmonary disease (COPD) is associated with poor outcomes. We sought to compare novel quantitative CT (QCT) measurements of pulmonary vascular volumes of COPD patients with and without associated PH and to explore the associations with clinical characteristics, hemodynamics and outcomes.

We retrospectively included 45 patients with right heart catheterization (RHC)‐confirmed COPD‐PH and 42 patients with COPD seen at Mayo Clinic Sites in Minnesota, Arizona and Florida between 2000 and 2020. Pulmonary vascular volumes were extracted from CT scans obtained within 30 days of confirmatory RHC or echocardiography using an automated algorithm and divided into BV5 and BV10, denoting the volumes of blood vessels with cross‐sectional areas <5 mm^2^ or >10 mm^2^, respectively, as a percentage of total blood volume. The emphysema index was also computed. Clinical characteristics and pertinent studies were collected. Median follow‐up was 9.5 years. No differences were found in baseline and demographic characteristics. Patients had 1‐, 5‐ and 10‐year survival of 88% (80.17, 98.5), 70.4% (57.8, 85.3) and 25.6% (13.5, 47.7) for COPD‐PH and 100%, 88.7% (78.8, 99.8) and 56% (39.5, 79.4) for COPD. Patients with COPD‐PH had a significantly higher BV10 (*p* = 0.003), a significantly lower BV5 (*p* = 0.0016) and a lower emphysema index (*p* = 0.0003) than those without PH. Increased risk of mortality endpoints in univariate analysis were: presence of PH [hazard ratio (HR) = 2.32 (1.26, 4.27), *p* = 0.007], higher BV10 [HR = 1.08 (1.01, 1.16), *p* = 0.021], lower BV5 [HR = 0.64 (0.46, 0.87), *p* = 0.006], lower DLCO %predicted, per 10 points [HR = 0.73 (0.62, 0.87), *p* < 0.001]. The presence of PH increases mortality in COPD patients. Quantification of vascular volumes on CT scan in patients with RHC‐confirmed PH demonstrates a characteristic signature distinct from those without PH. This method could potentially identify comorbid PH non‐invasively. Prospective studies are required to confirm these findings.

## A035 LONG‐TERM FOLLOW‐UP OF HIV‐ASSOCIATED PULMONARY HYPERTENSION: CLINICAL FEATURES AND SURVIVAL OUTCOMES OF THE PAN AFRICA PULMONARY HYPERTENSION COHORT (PAPUCO)

Friedrich Thienemann, Patrick D M C Katoto, Feriel Azibani, Vitaris Kodogo, Sandra L Mukasa, Mahmoud U Sani, Kamilu M Karaye, Irina Mbanze, Ana O Mocumbi, Anastase Dzudie, Karen Sliwa

*Cape Heart Institute, Faculty of Health Science, University of Cape Town, Cape Town; Department of Medicine, Faculty of Health Sciences, University of Cape Town, Cape Town, South Africa; Department of Internal Medicine, University Hospital Zurich, University of Zurich, Zurich, Switzerland; Department of Medicine and Center for Infectious Diseases, Faculty of Medicine and Health Sciences, Stellenbosch University, Cape Town, South Africa; Department of Medicine, Division of Respiratory Medicine & Prof. Lurhuma Biomedical Research Laboratory, Mycobacterium Unit, Catholic University of Bukavu, Bukavu, DR Congo; INSERM U942, Paris, France; Department of Medicine, Bayero University Kano & Aminu Kano Teaching Hospital, Kano, Nigeria; Faculty of Medicine, Eduardo Mondlane University, Maputo, Mozambique; Instituto Nacional de Saúde, Mozambique; Department of Internal Medicine, Douala General Hospital, Cameroon*

Data characterizing risk factors and long‐term outcome studies on human immunodeficiency virus (HIV)‐associated pulmonary hypertension (PH) in Africa are lacking. The Pan African Pulmonary Hypertension Cohort (PAPUCO), a prospective, multinational registry of 254 consecutive patients diagnosed with PH (97% of African descent) from nine specialist centres in four African countries was implemented. We compared baseline characteristics and 3‐year survival of a newly diagnosed cohort of patients with PH and HIV (PH/HIV + ) with an HIV‐uninfected control cohort with PH (PH/HIV − ). There were 134 cases of PH who completed the follow‐up (47 PH/HIV + , 87 PH/HIV − ; median age 36 vs. 44 years; *p* = 0.0004). Cardiovascular risk factors and comorbidities were similar except for previous tuberculosis (TB; 62 vs. 18%, *p* < 0.0001). Six‐minute walk distance (6MWD) < 300 m was common in PH/HIV − (*p* = 0.0030), but PH/HIV + had higher heart (*p* = 0.0160) and respiratory (*p* = 0.0374) rates. Thirty‐six per cent of PH/HIV + and 15% of PH/HIV − presented with pulmonary arterial hypertension (PAH; *p* = 0.0084), whereas 36% of PH/HIV + and 72% of PH/HIV − exhibited PH attributable to left heart disease (PHLHD; *p* = 0.0009). PH attributable to lung diseases and hypoxia (PHLD) was frequent in PH/HIV + (36 vs. 15%) but did not reach statistical significance. HIV‐associated PAH tended to have a poorer survival rate compared with PHLHD/PHLD in HIV‐infected patients.

PH/HIV + patients were younger and commonly had previous TB compared with PH/HIV − patients. Despite a better 6MWD at presentation, they had more signs and symptoms of early onset heart failure and a worse survival rate. Careful clinical evaluation is warranted, and early echocardiography assessment should be performed, especially in HIV‐infected patients with a history of TB.

## A036 EPIGENETIC REACTIVATION OF TRANSCRIPTION PROGRAMS ASSOCIATED WITH FETAL LUNG DEVELOPMENT COORDINATES ABERRANT CELLULAR PHENOTYPES IN HUMAN PULMONARY HYPERTENSION

Prakash Chelladurai, Carsten Kuenne, Alice Bourgeois, Stefan Günther, Chanil Valasarajan, Anoop V Cherian, Robbert J Rottier, Charlotte Romanet, Andreas Weigert, Olivier Boucherat, Christina A Eichstaedt, Clemens Ruppert, Andreas Guenther, Thomas Braun, Mario Looso, Rajkumar Savai, Werner Seeger, Uta‐Maria Bauer, Sébastien Bonnet, Soni Savai Pullamsetti

*Max Planck Institute for Heart and Lung Research, Member of the German Center for Lung Research (DZL), Member of the Cardio‐Pulmonary Institute (CPI), Bad Nauheim, Germany; Pulmonary Hypertension and Vascular Biology Research Group of Quebec Heart and Lung Institute, Department of Medicine Laval University, Quebec, Canada; Department of Pediatric Surgery, Erasmus Medical Center – Sophia Children's Hospital, Wytemaweg 80, 3015 CN Rotterdam, The Netherlands; Department of Cell Biology, Erasmus Medical Center, Rotterdam, The Netherlands; Institute of Biochemistry I, Goethe‐University Frankfurt, Frankfurt, Germany; Centre for Pulmonary Hypertension, Thoraxklinik Heidelberg GmbH at Heidelberg University Hospital, Translational Lung Research Center Heidelberg (TLRC), Member of the German Center for Lung Research (DZL), Laboratory for Molecular Diagnostics, Institute of Human Genetics, Heidelberg University, Heidelberg, Germany; Department of Internal Medicine, Member of the DZL, Member of CPI, Justus Liebig University, Giessen, Germany; Institute for Lung Health (ILH), Member of the DZL, Justus Liebig University, Giessen, Germany; Institute of Molecular Biology and Tumor Research, Marburg, Germany*

Phenotypic alterations in resident vascular cells predominantly contribute to the aggravation of the vascular remodeling process in pulmonary arterial hypertension (PAH), which culminates in right ventricular failure. It remains poorly explored how the regulatory interplay between transcription factors, transcriptional coactivators and alterations in chromatin state (at gene promoters and distal enhancers) coordinates the maintenance of ‘persistently activated cellular phenotypes’ via aberrant transcriptional responses. Chromatin immunoprecipitation‐sequencing (ChIP‐seq) revealed genome‐wide differential distribution of active chromatin signatures (histone H3 and H4), including alterations at 4,152 active enhancers (H3K27ac) in PAH‐FBs. Integrative analysis of RNA‐seq and ChIP‐seq data revealed the association of PAH‐specific active enhancers with mesenchyme development, mesenchymal cell differentiation and migration. Remarkably, transcriptional signatures for lung morphogenesis were epigenetically derepressed in PAH‐FBs, including coexpression of *TBX4*, *TBX5* and *SOX9*, which are typically expressed during early stages of lung development. These transcription factors (TFs) were strongly expressed initially in mouse fetuses and repressed postnatally but were maintained in the tissues from persistent PH of the newborn and re‐expressed in adult PAH. Besides alterations in histone acetylation levels at promoters and enhancers of *TBX4*, *TBX5* and *SOX9*, we observed massive epigenome‐wide alterations in the levels of H3K27ac in PAH‐FBs, which eventually correspond to dysregulated activity of the P300/CBP histone acetyltransferase complex. Notably, silencing of *TBX4*, *TBX5*, *SOX9* or *EP300* by RNA interference or small‐molecule compounds regressed PAH phenotypes, mesenchymal signatures in arterial FBs and smooth muscle cells and reduced vascular remodeling in precision‐cut tissue slices from human PAH lungs *ex vivo*. Pharmacological inhibition of P300/CBP complex activity significantly attenuated the expression of molecular markers of PAH, mesenchymal transition markers, remodeling of distal pulmonary vessels, improved hemodynamics and reversed established PAH in three rodent models in vivo. Overall, application of integromic approaches has delineated epigenetic reactivation of developmental TF networks that contributes to PAH pathogenesis, opening new therapeutic avenues.

## A037 THE GLI1^+^ MESENCHYMAL STROMAL CELL IS A KEY CONTRIBUTOR TO NEO‐MUSCULARIZATION IN PULMONARY HYPERTENSION AND AN IMPORTANT CELLULAR THERAPEUTIC TARGET

Xuran Chu, Vahid Kheirollahi, Ana Ivonne Vazquez‐Armenderiz, Stefan Hadzic, Oleg Pak, Monika Heiner, Simone Kraut, Ali Khadim, Arun Lingampally, Stefan Günther, Janine Koepke, Guillerme Valente, Thomas Braun, Christos Samakovlis, Mario Looso, Susanne Herold, Werner Seeger, Norbert Weissmann, Jin San Zhang, Xiaokun Li, Elie El Agha and Saverio Bellusc

*School of Pharmaceutical Sciences, Wenzhou Medical University, Wenzhou, Zhejiang, China; Cardio‐Pulmonary Institute, Universities of Giessen and Marburg Lung Center (UGMLC), member of the German Center for Lung Research (DZL), Justus‐Liebig University Giessen, Giessen, Germany; Institute for Lung Health (ILH), Giessen, Germany; Max Planck Institute for Lung and Heart, Bad Nauheim, Germany; Key Laboratory of Interventional Pulmonology of Zhejiang Province, Department of Pulmonary and Critical Care Medicine, The First Affiliated Hospital of Wenzhou Medical University, Wenzhou, China*

Pulmonary hypertension occurs when the pressure in the blood vessels leading from the heart to the lungs is too high. During pulmonary hypertension, the blood vessels to the lungs develop an increased number of smooth muscle cells (SMCs) in the wall of the blood vessels. To investigate the origin of the neo‐muscularization is crucial to find potential therapies for PH. Previous studies have shown that pre‐existing SMCs are the primarily source of newly formed SMCs via clonal expansion in PH. In our study, we used *Gli1* ^*Cre‐ERT2*^ mice to trace the lineage of GLI1^+^ cells in PH during vascular remodeling and a reverse remodeling phase and showed that GLI1^+^ cells contribute to the neo‐muscularization in PH and resolution of PH in the reverse remodeling phase. We also used the *Gli1* ^*Cre‐ERT2*^; *iDTR* ^*flox*^ line to carry out genetic ablation of GLI1^+^ cells in mice to investigate the pathological role of GLI1^+^ cells in PH. Genetic ablation of GLI1^+^ cells at the beginning of hypoxic injury protected lungs from PH development, confirmed by echocardiography, FACS and IF analysis. Furthermore, IF analysis on PCLS showed that GLI1^+^ cells contributed to the newly formed ACTA2^+^ cells in distal blood vessels through local recruitment and transdifferentiation in hypoxia‐induced PH. scRNA‐seq on GLI1^+^ cells obtained from hypoxia‐induced PH mice indicated that the GLI1^+^ cell lineage was highly heterogenous. Among 12 clusters found in the scRNA‐seq, we found cluster 9 containing the signature genes of vascular SMCs, and the cells in cluster 9 were enriched in hypoxia‐induced PH condtions. In conclusion, the GLI1^+^ cell lineage is a main source of newly formed vascular SMCs in PH via local recruitment and transdifferentiation and represent a therapeutic target to treat PH.

## A038 A JUNCTOPHILIN‐2–MITOFUSIN‐2 INTERACTION IS IMPORTANT FOR MAINTAINING MITOCHONDRIAL FATTY ACID OXIDATION IN PULMONARY ARTERIAL HYPERTENSION‐MEDIATED RIGHT VENTRICULAR FAILURE

Sasha Z Prisco, Megan M Eklund, Lynn M Hartweck, Kurt W Prins

*Cardiovascular Division, Lillehei Heart Institute, Department of Medicine, University of Minnesota, Minneapolis, MN, USA*

Junctophilin‐2 (JPH2) plays a crucial role in t‐tubule structure/function and gene regulation in cardiomyocytes. Moreover, JPH2 downregulation causes mitochondrial dysfunction and mitofusin‐2 (MFN2) dysregulation, but the link between JPH2 and mitochondria is undetermined. Here, we investigated the hypothesis that a direct JPH2–MFN2 interaction is important for mitochondrial regulation and subsequent right ventricular (RV) function in pulmonary arterial hypertension (PAH). Confocal microscopy revealed JPH2 and MFN2 colocalized at t‐tubules in isolated cardiomyocytes. Immunoprecipitation demonstrated that JPH2 and MFN2 interacted in RV extracts. Pulldown experiments showed that JPH2 bound MFN2 directly and that the MFN2 binding domain was localized to the N‐terminal third of JPH2. To probe the importance of the JPH2–MFN2 interaction for RV function, we compared the effects of treatment with AAV9‐JPH2 with AAV9‐GFP in monocrotaline‐induced PAH. Immunoblots of RV extracts demonstrated that AAV9–JPH2 increased JPH2 and MFN2 expression. AAV9–JPH2 treatment prevented disruption of mitochondrial cristae structure as revealed by electron microscopy. Pathway analysis of quantitative proteomics of RV mitochondrial enrichments demonstrated that fatty acid oxidation (FAO) was a key pathway for differentiating the three groups. AAV9–JPH2 increased levels of several FAO proteins, which subsequently prevented accumulation of lipid droplets in the RV. At the organ level, AAV9–JPH2 reduced RV cardiomyocyte hypertrophy and RV fibrosis. AAV9–JPH2 increased tricuspid annular plane systolic excursion and RV free wall thickening as quantified by echocardiography. Pressure–volume loop analysis showed that AAV9–JPH2 augmented RV ejection fraction and right ventricular–pulmonary arterial coupling and lowered RV end‐diastolic pressure and tau. Importantly, the improvements in RV systolic and diastolic function were independent of PAH severity because RV systolic pressure, effective arterial elastance and pulmonary arterial small vessel remodeling were not altered with AAV9–JPH2.

A JPH2–MFN2 interaction is important for FAO in the RV in PAH. Strategies to increase JPH2 expression could have therapeutic value for RV failure.

## A039 ENGINEERED PULMONARY ARTERY TISSUES (EPATS)—A NOVEL TECHNIQUE TO ASSESS VASCULAR CONTRACTILITY IN VITRO

Adam Fellows, Katie Quigley, Alex Ainscough, Harry Barnett, David Miller, Beata Wojciak‐Stothard

*National Heart & Lung Institute, Imperial College London, London; Imperial College Advanced Hackspace, Imperial College London, London, UK*

Conventional monolayer culture of vascular smooth muscle cells suppresses their contractile phenotype, a property crucial to cardiovascular function and disease. Therefore, we developed a novel in vitro three‐dimensional (3D) culture platform using hydrogels containing human pulmonary artery smooth muscle cells – ‘engineered pulmonary artery tissues’ (EPATs). The aim was to allow rapid and direct measurement of vasoreactivity in vitro. The EPATs were produced using custom‐made racks fabricated from polydimethylsiloxane (PDMS) using a 3D‐printed resin mould. Primary human pulmonary artery smooth muscle cells were suspended with fibrinogen and seeded between pairs of posts hanging from the PDMS racks. The 3D constructs were formed after fibrin polymerization by thrombin. Auxotonic stretch exerted by the PDMS posts is designed to re‐establish contractility within smooth muscle cells. This method was adapted from a published protocol for ‘engineered heart tissues’. Crucially, EPATs were able to mimic both vasoconstriction and vasodilation in response to various vasoactive reagents, indicated by flexion of the posts and subsequent measurements of changes in EPAT length using time‐lapse microscopy. Contractility was apparent 7 days after fabrication, and changes were robust and reproducible over multiple experiments. Importantly, EPATs are viable for >3 weeks, enabling both long‐term treatments and repeated measurements over time. Finally, concentration–response curves were generated for endothelin‐1 and imatinib, which are both highly relevant for the modelling and treatment of pulmonary arterial hypertension. The EPAT methodology provides the unique ability to reproduce SMC vasoactivity in vitro and has enormous potential to address the clinical need for better vasodilatory therapies for diseases such as pulmonary arterial hypertension.

## A040 ENDOTHELIAL ADP‐RIBOSYLATION FACTOR 6 ACTIVATES HYPOXIA‐INDUCIBLE FACTOR 2α: IMPLICATIONS FOR PULMONARY ARTERIAL HYPERTENSION AND THE RESPONSE TO HYPOXIA

Adam Fellows, Sandro Satta, Nayana Iyer, Vahitha Abdul‐Salam, Lukas Schmidt, Xiaoke Yin, Manuel Mayr, Andrew Cowburn, Beata Wojciak‐Stothard

*National Heart & Lung Institute, Imperial College London, London; King's British Heart Foundation Centre, King's College London, London, UK*

ADP‐ribosylation factor 6 (ARF6) is a GTPase highly expressed in the human pulmonary artery endothelium and previously has been promoted as a therapeutic target for pulmonary arterial hypertension (PAH). However, the precise role of ARF6 in the context of this disease is unknown. The aim was to determine the cellular consequences of endothelial ARF6 activation and identify downstream pathophysiological pathways relevant to PAH. Human pulmonary artery endothelial cells were transduced with adenoviruses to induce ARF6 overexpression and model the ARF6 hyperactivation observed in PAH. After 24 h, whole‐cell lysates were analysed by proteomics, and associated biological pathway changes were determined using multiple bioinformatics databases. Overexpression of constitutively active ARF6 resulted in the upregulation of various hypoxia‐related pathways. This was accompanied by activation of hypoxia‐inducible factor 2α (HIF‐2α), as shown by enhanced nuclear protein localization and activation of a HIF‐dependent luciferase reporter. For the first time, we also demonstrated that ARF6 is activated during exposure to hypoxia, with a temporal regulation profile similar to that of HIF‐2α (peak activation at ~16 h). Finally, we investigated the translational potential of chlortetracycline, a veterinary antibiotic and newly discovered ARF6 inhibitor, in conditions of low oxygen. Chlortetracycline specifically attenuated HIF‐2α accumulation in response to acute hypoxia both in vitro and in vivo in human pulmonary artery endothelial cells and in mouse lungs, respectively. Hypoxia‐inducible factor 2α has recently emerged as a key contributor to both PAH and hypoxia‐induced pulmonary hypertension. We have shown that HIF‐2α activation is mediated by ARF6, which can be diminished with the administration of chlortetracycline, a clinically approved antibiotic.

## A041 TARGETING EUKARYOTIC TRANSLATION INITIATION FACTOR 5A HYPUSINATION: AN OPPORTUNITY TO COUNTER PULMONARY VASCULAR REMODELING IN PULMONARY ARTERIAL HYPERTENSION

Sarah‐Eve Lemay, Yann Grobs, Ghada Mkannez, Tsukasa Shimauchi, Sandra Breuils‐Bonnet, François Potus, Steeve Provencher, Sébastien Bonnet, Olivier Boucherat

*Pulmonary Hypertension Research Group, Québec Heart and Lung Institute Research Centre, Québec, Canada*

Pulmonary arterial hypertension (PAH) is characterized by progressive obstruction of the pulmonary arteries (PAs) leading to heart failure and death. Pulmonary arterial smooth muscle cells (PASMCs) of PAH patients display a ‘cancer‐like’ pro‐proliferative and apoptosis‐resistant phenotype that contributes to distal PA remodeling. Appreciation of the pivotal role of translational control in hyperproliferating diseases is steadily increasing. In this regard, eukaryotic translation initiation factor 5 A (eIF5A) was recently shown to provide cancer cells with a competitive advantage by increasing translation of specific mRNA with oncogenic properties. Strikingly, eIF5A is the only protein containing the unique, polyamine‐derived amino acid hypusine, which is required for its function. Hypusine formation occurs post‐translationally and is catalyzed by the sequential actions of deoxyhypusine synthase (DHPS) and deoxyhypusine hydrolase (DOHH). We hypothesized that hypusine signaling is increased in PAH and contributes to pulmonary vascular remodeling. Data derived from our comparative proteomic analysis (LC‐MSMS) between normal and PAH‐PASMCs and confirmed by western blot indicate that the hypusine‐forming enzymes DHPS and DOHH are highly overexpressed in PAH‐PASMCs compared with controls (*p* < 0.05). Consistently, total and hypusinated forms of eIF5A were found to be upregulated in dissected PAs and PASMCs from PAH patients and animal models (MCT and Su/Hx rats, *p* < 0.05). *In vitro*, pharmacological inhibition of DHPS and DOHH using GC7 and ciplopirox, respectively, significantly reduced PAH‐PASMCs survival and proliferation (Annexin V; Ki67 labeling and EdU incorporation, *p* < 0.01). These effects were accompanied by reduced expression of eiF5A^Hyp^, MCM2, PCNA, Survivin and BRD4 (*p* < 0.01). *In vivo*, preliminary data indicate that pharmacological inhibition of DHPS using GC7 in MCT rats with established PAH improves hemodynamics (right heart catheterization: RVSP, mPAP, CO, *p* < 0.05) and vascular remodeling (Elastica van Gieson staining, *p* < 0.05). We showed, for the first time, that hypusine signaling is implicated in PAH development and represents a new promising therapeutic target to improve PAH.

## A042 PULSAR OPEN‐LABEL EXTENSION: LONG‐TERM EFFICACY AND SAFETY OF SOTATERCEPT FOR THE TREATMENT OF PULMONARY ARTERIAL HYPERTENSION (PAH)

Marc Humbert, Vallerie McLaughlin, J. Simon R. Gibbs, Mardi Gomberg‐Maitland, Marius M Hoeper, Ioana R Preston, Rogerio Souza, Aaron Waxman, Hossein‐Ardeschir Ghofrani, Pilar Escribano Subias, Jeremy Feldman, Gisela Meyer, David Montani, Karen Olsson, Solaiappan Manimaran, Janethe de Oliveira Pena, David B Badesch

*Department of Respiratory and Intensive Care Medicine, Hôpital Bicêtre, Assistance Publique–Hôpitaux de Paris, INSERM Unité Mixte de Recherche 999, Université Paris‐Saclay, Le Kremlin‐Bicêtre, France; Division of Cardiovascular Medicine, Department of Internal Medicine, University of Michigan Health System, Ann Arbor, MI, USA; National Heart and Lung Institute, Imperial College London, and the National Pulmonary Hypertension Service, Hammersmith Hospital, Imperial College Healthcare NHS Trust, London, UK; Department of Medicine, George Washington University School of Medicine and Health Sciences, Washington, DC, USA; Department of Respiratory Medicine, Hannover Medical School, and the German Center for Lung Research, Hannover, Germany; Division of Pulmonary, Critical Care and Sleep Medicine, Tufts Medical Center, Boston, MA, USA; Pulmonary Division–Heart Institute, University of São Paulo Medical School, São Paulo, Brazil; Division of Pulmonary and Critical Care Medicine, Department of Medicine, Brigham and Women's Hospital, Boston, MA, USA; Department of Pneumology, University of Giessen and Marburg, Giessen, Germany; Department of Cardiology, Centro de Investigación en Red en Enfermedades Cardiovasculares, Hospital Universitario 12 de Octubre, Universidad Complutense, Madrid, Spain; Arizona Pulmonary Specialists, Phoenix, AZ, USA; Complexo Hospitalar Santa Casa de Porto Alegre, Pulmonary Vascular Research Institute, Porto Alegre, Brazil; Acceleron Pharma (a wholly owned subsidiary of Merck & Co., Inc.), Cambridge, MA, USA; Division of Pulmonary Sciences and Critical Care Medicine, and Cardiology, University of Colorado, Anschutz Medical Campus, Aurora, CO, USA*

Pulmonary arterial hypertension (PAH) is a progressive disease driven by pulmonary vascular remodeling. Sotatercept acts as a reverse‐remodeling agent proposed to rebalance antiproliferative (BMPR‐II‐mediated) and pro‐proliferative (ActRIIA‐mediated) signaling. In preclinical models of PAH, sotatercept reversed pulmonary arterial wall and right ventricular pathologic remodeling. PULSAR (NCT03496207) is a phase 2, randomized, double‐blind, placebo‐controlled study followed by an open‐label extension (OLE) period evaluating sotatercept on top of background PAH therapy in World Health Organization functional class (WHO FC) II/III PAH participants. Participants originally randomized to placebo were re‐randomized 1:1 to sotatercept 0.3 or 0.7 mg/kg (placebo‐crossed group). Previously treated sotatercept participants continued the same dose (continued‐sotatercept group). Safety was evaluated in all participants who received at least one dose of sotatercept. The primary efficacy endpoint was change from baseline to months 18–24 in pulmonary vascular resistance (PVR). Secondary endpoints included 6‐min walk distance (6MWD) and WHO FC. Two pre‐specified analyses (placebo‐crossed and delayed‐start) evaluated efficacy endpoints irrespective of sotatercept dose. Efficacy data at 18–24 months and cumulative safety as of 8 June 2021 are reported. Of 106 participants initially enrolled in the PULSAR study, 97 (92%) continued into the OLE period. Serious treatment‐emergent adverse events (TEAEs) were reported in 32 (31%) participants; 10 (10%) reported TEAEs leading to study discontinuation and 3 (3%) died, none considered related to study drug. The placebo‐crossed group demonstrated statistically significant improvements in PVR, 6MWD and WHO FC from baseline to months 18–24 (*p* < 0.0001), while the continued‐sotatercept group at least maintained improvement. Sotatercept was well tolerated, and safety is consistent with previous reports. Significant clinical improvement across multiple endpoints was achieved following 12–18 months of sotatercept treatment in previous placebo participants; clinical efficacy was maintained or further enhanced in those with longer‐term use. These results demonstrate the potential longer‐term durability of clinical benefit with sotatercept in participants with this progressive disease.

## A043 DICARBONYL STRESS DEPRESSES MITOCHONDRIAL FATTY ACID OXIDATION AND PROMOTES RIGHT VENTRICULAR DYSFUNCTION IN PULMONARY ARTERIAL HYPERTENSION

Sasha Z Prisco, Lynn Hartweck, Jennifer L Keen, Felipe Kazmirczak, Megan Eklund, Kurt W Prins

*Lillehei Heart Institute, Cardiovascular Division, Department of Medicine, University of Minnesota, Minneapolis, MN, USA*

The mechanisms underlying right ventricular (RV) dysfunction in pulmonary arterial hypertension (PAH) are incompletely defined, but multiple studies reveal RV metabolic derangements characterized by heightened glycolytic flux. Methylglyoxal, a byproduct of glycolysis, is a highly reactive dicarbonyl that has toxic effects via nonenzymatic posttranslational modifications (protein glycation). Methyglyoxal is degraded by the glyoxylase system, which includes the rate‐limiting enyzme glyoxylase‐1 (Glo1), to protect cells from dicarbonyl stress. We first performed a bioinformatics analysis of previously identified glycated proteins and found a relationship between mitochondrial metabolic function and protein glycation. To validate this *in silico* analysis, we evaluated how exogenous methylglyoxal impacted mitochondrial function in H9c2 cardiomyoblasts. Methylglyoxal minimally altered mitochondrial activity when cells metabolized glucose; however, methylglyoxal significantly depressed fatty acid oxidation. Then, we defined the effects of adeno‐associated virus serotype 9‐mediated (AAV9) overexpression of Glo1 in the RV of monocrotaline rats. AAV9 treatment increased RV cardiomyocyte Glo1 expression, which significantly reduced total protein glycation. AAV‐Glo1 treatment partly mitigated depressed mitochondrial density and pathological lipid accumulation. Glo1 overexpression decreased RV hypertrophy and RV fibrosis and improved RV systolic and diastolic function as assessed by both echocardiography and closed‐chest pressure–volume loop analyses. Importantly, the changes in RV structure/function were not attributable to differences in PAH severity. Our findings suggest that dampening excess protein glycation might enhance mitochondrial fatty acid oxidation and RV function in PAH.

## A044 NONINVASIVE SCORE TO PREDICT PRECAPILLARY PULMONARY HYPERTENSION

Himanshu Deshwal, Eric Bondarsky, David Morales, Ashish Rai, Shilpa DeSouza, Roxana Sulica

*Division of Pulmonary, Sleep and Critical Care Medicine, Department of Internal Medicine, New York, University Grossman School of Medicine, New York, NY, USA*

Delays in precapillary pulmonary hypertension (PH) diagnosis and treatment are associated with poor outcomes. A noninvasive scoring system might provide rationale for timely referral to PH centers and earlier treatment. We included in this observational study all patients newly referred to our PH program who underwent right heart catheterization (RHC) from 31 July 2017 to 1 January 2022. We collected clinical parameters from guideline‐recommended noninvasive diagnostic testing. Patients were randomly divided into derivation and validation cohorts. We identified independent predictors of precapillary PH by multivariate logistic regression analysis, and we derived a weighted score for each variable. Receiver operator curves and area under the curve (AUC) analysis were used to assess the performance of the derived score. We identified 256 patients (median age 66 years, 73% women), of whom 181 were assigned to the derivation and 75 to the validation cohort. Independent predictors of precapillary PH (found in 65% of patients) and respective weighted scores were diffusing lung capacity ≤ 60% (+1), right axis deviation (+4), dilated left atrium (−1), moderate‐to‐severe mitral regurgitation (−4), tricuspid valve regurgitation velocity ≥ 2.8 m/s (+2), contrast reflux in the hepatic veins (+2), pulmonary artery ≥ 3 cm (+1), and at least one segmental ventilation/perfusion mismatch (+2). The score had a good performance on internal (AUC of 0.85) and external (AUC of 0.80) validation. In derivation and validation cohorts, a score ≥ 1 had a sensitivity of 98.3% and 98%, with a negative predictive value of 86.7% and 85.7%, respectively, whereas a score ≥ 7 had a specificity of 98.4% and 96.4%, with a positive predictive value of 97% and 94%, respectively, with a 97% probability of precapillary PH. In conclusion, screening for precapillary PH might be facilitated by a noninvasive scoring system. Generalizability of this model requires prospective validation.

## A045 SEX DIFFERENCES IN ACTIVATION OF PULMONARY ARTERIAL ADVENTITIAL FIBROBLAST PROFIBROTIC GENES WHEN CULTURED IN VITRO

Ariel Wang, Daniela Valdez‐Jasso,

*Department of Bioengineering, University of California, San Diego, La Jolla, CA, USA*

Pulmonary arterial hypertension (PAH) is a vasculopathy to which women are more susceptible, but they fare better than men. It is characterized by an increase in mean arterial pressure, resulting in changes in circumferential strain and stiffening of the extracellular matrix (ECM), which is mediated by pulmonary arterial adventitial fibroblasts (PAAFs). We cultured PAAFs from control male and female rats on hydrogels formulated with stiffnesses to represent normotensive (0.5 kPa), mild PAH (3 kPa) and severe PAH (10 kPa) conditions and stretched equibiaxially at 10% for 24 h. At a stiffness of 0.5 kPa, male PAAFs responded significantly to stretch in six genes (*Col1a1*, *Col3a1*, *Acta2, Fn1*, *Loxl1* and *Eln*), while female PAAFs had a significant effect of stretch only for *Acta2* and *Fn1*. At 3 kPa, male PAAFs responded to stretch in *Col1a1*, *Col3a1*, *Acta2*, *Loxl1* and *Eln*, while female‐derived cells responded to stretch in genes *Col1a1* and *Acta2*. When cultured on the stiffer 10 kPa substrates, male PAAF genes *Col1a1*, *Col3a1*, *Eln* and *Loxl1* were upregulated in response to stretch, while female PAAFs were downregulated in response to stretch for *Col3a1*. Male‐derived cells exhibited a monotonic increase of gene expression from 0.5 to 3 kPa and from 3 to 10 kPa, whereas female cell gene expressions were not significantly different between 0.5 and 3 kPa, but there was a significant increase at 10 kPa, indicative of a higher threshold to the effects of stiffness than male‐derived cells. Female‐derived cells also displayed less sensitivity to stretch than male cells, which could affect activation of female PAAFs in response to mild PAH. These experimental findings suggest a lower sensitivity of female PAAFs to elevated ECM stiffness, which might explain why female rats, and patients, exhibit less severe vascular stiffening and subsequent hemodynamic dysfunction in PAH.

## A046 IMPLICATION OF FIBRONECTIN‐BINDING INTEGRINS IN MALADAPTIVE LUNG AND RIGHT VENTRICULAR REMODELING IN PULMONARY ARTERIAL HYPERTENSION

Sarah‐Eve Lemay, Mónica S Montesinos, Yann Grobs, Tetsuro Yokokawa, Tsukasa Shimauchi, Sandra Breuils‐Bonnet, Sandra Martineau, Ghada Mkannez, Alice Bourgeois, Charlotte Romanet, Xinqiang Huang, James E Dowling, Min Lu, Adrian S Ray, François Potus, Steeve Provencher, Olivier Boucherat, Sébastien Bonnet,

*Pulmonary Hypertension Research Group, Québec Heart and Lung Institute Research Center, Québec, Canada; Morphic Therapeutic, Waltham MA, USA*

Pulmonary arterial hypertension (PAH) is characterized by progressive obstruction of pulmonary arteries (PAs), culminating in right ventricular (RV) failure and premature death. Like cancer cells, PA smooth muscle cells (PASMCs) isolated from PAH patients exhibit exaggerated proliferation and resistance to apoptosis in response to extracellular matrix (ECM) remodeling. Integrins, members of the cell adhesion receptor superfamily, are known to promote cell proliferation, survival, hypertrophic growth and fibrosis, which are key processes leading to PA remodeling and RV failure. Therefore, we hypothesized that in PAH, integrin signaling promotes PAH‐PASMCs proliferation and resistance to apoptosis, contributing to PA vascular remodeling and RV cardiomyocyte maladaptive hypertrophy and RV fibrosis, leading to RV failure. By western blot (WB), we showed that fibronectin‐binding integrin (FiBIs) expression is significantly changed in distal PAs, PASMCs, PA endothelial cells and decompensated RV isolated from PAH patients compared with controls (*n* = 5–8). *In vitro*, pharmacological inhibition of FiBIs decreased PAH‐PASMCs proliferation (WB: PLK1, MCM2 and Ki67 labeling) and resistance to apoptosis (WB: Survivin and Annexin V labeling) (*p* < 0.05, *n* = 6). These effects were associated with a decreased activation of FiBIs downstream signaling, FAK and MAPK pathways. In adult rat cardiomyocytes and human RVFbs, inhibition of FiBIs decreased phenylephrine‐induced hypertrophy (*f*‐actin labeling) and TGFβ1‐induced RVFbs activation (WB: αSMA, CTGF, FN and Col1) (*p* < 0.05, *n* = 5). In both moncrotaline and sugen/hypoxia rats with established PAH, pharmacological FiBIs inhibition alone or in combination with macitentan and tadalafil improved hemodynamic (RVSP, mPAP, SV, CO, TAPSE, S‐Wave; *p* < 0.05, *n* = 7–19) and vascular remodeling (Elastica Van Gieson, *n* = 7–19; *p* < 0.01). In the PA banding rat model, FiBIs inhibition attenuated RV failure (SV, CO, TAPSE, S‐Wave, RVFAC; *p* < 0.001, *n* = 10). Our results suggest that integrins play a key role in the pathophysiology of PAH in both the lungs and the RV. Their inhibition provided significative beneficial effects both in vitro and in vivo.

## A047 SAFETY/EFFICACY OF RT234 VARDENAFIL INHALATION POWDER ON EXERCISE PARAMETERS IN PULMONARY ARTERIAL HYPERTENSION: PHASE 2 DOSE‐ESCALATION STUDY DESIGN

Raymond L Benza, Veronica Franco, Mandar A Aras, Leslie Spikes, Daniel Grinnan, Kevin Corkery, Carol Satler

*The Ohio State University Wexner Medical Center, Columbus, OH; University of California, San Francisco, San Francisco, CA, USA; University of Kansas Medical Center, Kansas City, KS; Virginia Commonwealth University School of Medicine, Richmond, VA; Respira Therapeutics Inc., Albuquerque, NM, USA*

Pulmonary arterial hypertension (PAH) is currently managed using chronic, scheduled treatments to improve exercise capacity and delay clinical worsening. Availability of an as‐needed (PRN) treatment might further enhance patient quality of life by rapidly resolving symptoms associated with physical activity. RT234 is a drug/device combination of vardenafil hydrochloride and the novel Axial Oscillating Sphere dry powder inhaler that is suitable for PRN use and has the potential to improve exercise capacity, physical activity and associated symptoms (i.?e., dyspnoea). The CL202 study [NCT04266197; September 2020‒December 2023 (expected completion)] is a multicenter, open‐label, dose‐escalation, phase 2b study designed to evaluate the safety and efficacy of RT234 on exercise parameters assessed by cardiopulmonary exercise testing (CPET) and 6‐min walk distance (6MWD) in patients with PAH. Up to 40 adult patients with right heart catherization‐confirmed World Health Organizaion Group I PAH on stable oral PAH‐specific (≤2) and/or inhaled therapy are being enrolled in two successive dose cohorts (0.5 mg; 1.0 mg). A Safety Monitoring Committee will decide whether to proceed with the 1.0 mg cohort or terminate the study. Safety measures include adverse events (at each of two single‐dose treatment days and for 30 days’ follow‐up) and acute physical and cardiac effects. Efficacy measures include changes in peak oxygen capacity (VO_2_; primary endpoint) and exertional symptoms from baseline (day 1) to 15 min post dose during CPET on day 8, and change in 6MWD from baseline (screening, day −28 to day −3) to 15 min post dose on day 15. Exploratory endpoints include pharmacokinetics and exposure–response analyses. The results of the CL202 study are expected to inform the design of phase 3 trials of RT234.

## A048 ACUTE HEMODYNAMIC IMPROVEMENT IN CHRONIC PULMONARY ARTERIAL HYPERTENSION ON DUAL THERAPY FOLLOWING RT234 INHALATION

Anne Keogh, Nathan Dwyer, Eugene Kotlyar, David Kaye

*St Vincent's Hospital, Darlinghurst, NSW; Royal Hobart Hospital, Hobart, TAS; The Alfred Hospital, Melbourne, VIC, Australia*

RT234 is an inhaled formulation of the phosphodiesterase type‐5 inhibitor (PDE5i) vardenafil, in development for episodic symptoms of pulmonary arterial hypertension (PAH). This phase 2a escalating‐dose trial evaluated acute changes in pulmonary vascular resistance (PVR) and other hemodynamic (HD) parameters in PAH patients on stable maintenance dual therapy. Three cohorts received RT234 0.2, 0.6 or 1.2 mg during right heart catheterization. Hemodynamic parameters were recorded up to 60 min post‐inhalation. Of 14 subjects enrolled, the mean ± SD age was 54 ± 14 years (79% female) and functional class was II (57%), III (36%) or IV (7%). All subjects took oral endothelin receptor antagonist and oral PDE5i. In the 0.2, 0.6 and 1.2 mg cohorts, respectively, mean PVR was 635 ± 344, 469 ± 431 and 579 ± 337 dyn/s/cm^−5^ at baseline and decreased by −6.6% (−22.2 to 2.7), −23.7% (−44.7 to −18.6) and −16.0% (−22.7 to −10.5) post‐inhalation. With 0.6 and 1.2 mg, PVR fell >10% at 5 min, a reduction sustained for ≥60 min. The PVR/systemic vascular resistance ratio changed by −8.0% (−27.1 to 14.1), −18.4% (−37.8 to 0.9) and −11.9% (−23.9 to −0.3) for the 0.2, 0.6 and 1.2 mg doses, respectively, indicating that the 0.6 mg dose might offer the greatest pulmonary selectivity. No clinically significant changes in systemic blood pressure or heart rate were observed. Change in arterial O_2_ tension was +1.8% (−13.5 to 27.4), +8.1% (−13.5 to 22.6) and +4.3% (−1.4 to 9.9) for the three dose levels. Improvements in pulmonary HD with 0.6 mg inhaled RT234 were on par with 20 mg oral vardenafil, but with less systemic hypotension and higher oxygenation. The only treatment‐related adverse events (AEs) were mild headache and mild throat irritation, each in a single subject. No respiratory AEs occurred. RT234 produced rapid reduction in PVR, sustained for ≥60 min, and was well tolerated. The optimally effective RT234 dose appears to be 0.6 mg. RT234 is well suited for development of as ‘as‐needed’ or pre‐emptive PAH therapy.

## A049 EVOLUTION OF THE HEMODYNAMIC PROPERTIES AND ARTERIAL WALL REMODELING IN PULMONARY ARTERIAL HYPERTENSION

H Mu, D Valdez‐Jasso

*Department of Bioengineering, University of California, San Diego, La Jolla, CA, USA*

Pulmonary arterial hypertension (PAH) is characterized by sustained elevation of mean pulmonary arterial pressure and vascular remodeling. Our preliminary data show that female and male PAH rats have different patterns of hemodynamic changes, while their vascular compliance decreases dramatically compared with control. We are building a one‐dimensional fluid model to explore how remodeling affects hemodynamics in different stages and sexes of PAH. Female and male Sprague–Dawley rats were injected with sugen 5416 (SUHX) and kept in a 10% O_2_ hypoxia chamber for 3 weeks to induce PAH. Taking the injection day as week 0, SUHX rats at week 6 and week 12 were selected for analysis. Control rats received no injection and were kept in normoxia. Rats underwent open‐chest surgery to measure pulmonary arterial flow and pressure simultaneously. The left and right pulmonary arteries were harvested to perform tubular biaxial mechanical testing and morphological measurements. Our model spans the main, left and right pulmonary arteries (MPA/LPA/RPA), with assumptions of incompressible, Newtonian blood and axisymmetric, inviscid, laminar flow. The blood flow is formulated with mass and momentum conservations and a linear thin‐wall state equation. The in vivo flow measurement is implemented as the inlet boundary condition. At the bifurcation, flow and pressure conservations are enforced. For the outlet boundary conditions of the LPA and RPA, three‐element Windkessel models are applied to quantify the effect of downstream vessels, in which the Windkessel parameters are calculated from the hemodynamic measurements. The simulated pressure resembles the measured in vivo pressure waveform. The wave propagation and reflection are observed in the simulated flow and pressure waveform. Flow waveforms are damped differently from the inlets to the outlets of each artery, and the LPA and RPA have slightly different waveforms, indicating that our simulation results reflect the difference in artery geometry and stiffness.

## A050 AUTOMATED HYPOPERFUSED LUNG VOLUME IN PROXIMAL AND DISTAL CHRONIC THROMBOEMBOLIC PULMONARY HYPERTENSION UNDERGOING PULMONARY THROMBOENDARTERECTOMY

Elizabeth Bird, Kyle Hasenstab, Nick Kim, Michael Madani, Atul Malhotra, Lewis Hahn, Seth Kligerman, Albert Hsiao, Francisco Contijoch

*Department of Bioengineering; Department of Radiology; Department of Medicne (Division of Pulmonary, Critical Care and Sleep Medicine); Department of Surgery, University of California, San Diego, La Jolla, CA, USA*

Pulmonary artery occlusions in chronic thromboembolic pulmonary hypertension (CTEPH) cause regional hypoperfusion. Variable occlusion and hypoperfusion patterns require imaging assessment of the extent of involvement (spatial amount and location) before pulmonary thromboendaterectomy (PTE). Using an automated deep learning algorithm in combination with intensity thresholding, perfusion deficits in each lobe and in total lung were analyzed from dual‐energy CT pulmonary angiograms (DECTPAs). We hypothesize that hypoperfused lung volume (HLV) will identify and quantify hypoperfusion in CTEPH, even with variability in disease location and spatial extent. We test this by evaluating whether HLV (1) separates CTEPH cases from controls, and (2) is correlated with pulmonary vascular resistance (PVR) in distal and proximal CTEPH cases. Hypoperfused lung volume was calculated retrospectively from DECTPA studies in pre‐PTE CTEPH cases and control cases without vascular findings obtained between 2018 and 2020. The receiver operating curve (ROC) was used to evaluate separation of CTEPH from controls, and pre‐PTE PVR and post‐PTE ΔPVR correlations were calculated for patients with proximal and distal (surgical disease level 3 or 4 in at least one lung) CTEPH. A CTEPH case with pre‐ and post‐PTE HLV is highlighted. Hypoperfused lung volume separated 51 CTEPH (47 ± 17 years, 52.9% female, *n* = 19 distal) cases from 96 unmatched control cases (51 ± 16 years, 70.8% female) with an ROC area under the curve = 0.84. Pre‐PTE PVR and post‐PTE ΔPVR correlated moderately with HLV in proximal (⍴_PVR_ = 0.62, p_PVR_ = 0.003; ⍴_ΔPVR_ = − 0.5, p_ΔPVR_ = 0.008) and distal (⍴_PVR_ = 0.81, p_PVR_ = 0.002; ⍴_ΔPVR_ = − 0.70, p_ΔPVR_ = 0.01) cases. The highlighted case shows visual HLV improvement after surgery, with decrease in HLV from 5.8% to 1.7% in the right upper lobe and from 10.5% to 3.4% in the right lower lobe. Hypoperfused lung volume quantified hypoperfusion in proximal and distal CTEPH cases, correlating with PVR and separating control cases. Future work will investigate the relationship of HLV with surgically accessible specimens and change in HLV after removal of these specimens.

## A051 MITOCHONDRIAL AND EXTRACELLULAR MATRIX PROTEIN IN DEVELOPMENT OF PULMONARY HYPERTENSION: A PROTEOMIC‐BASED STUDY

Payel Sen, Bachuki Shashikadze, Siyu Tian, Daphne Merkus

*Walter‐Brendel‐Centre of Experimental Medicine, University Hospital, LMU, Munich, Germany; Gene Center, LMU, Munich, Germany, Division of Experimental Cardiology, Department of Cardiology, Erasmus MC, University Medical Center Rotterdam, Rotterdam, The Netherlands*

Previous work by our collaborators on postcapillary pulmonary hypertension (PH) has shown that endothelial factors are important in development of PH in a swine model. They used pulmonary vein banding (PVB) of the lower lobes for 12 weeks to induce type II PH. The aim of our present study is to use a proteomic‐based approach on these swine lung tissues to identify cellular factors, which are influenced by alteration of pressure and flow, thus providing the impetus for development of PH. We analyzed the tissues from upper and lower lobes to investigate whether there are differences in protein expression attributable to PH by a label free quantification using Liquid chromatography–tandem mass spectrometry. Data analysis and statistics were done using Perseus bioinformatics software. Functional annotation of differentially abundant proteins using DAVID bioinformatics demonstrated that compared with the lungs of control animals, several secreted extracellular matrix (ECM) proteins were downregulated in the upper lobe of PVB animals, and mitochondrial proteins were among the most upregulated proteins. Volcano plots indicated several individual mitochondrial proteins to be upregulated along with several ECM proteins, such as vitronectin, plasminogen, etc., to be downregulated more than twofold (*p* < 0.05) in the upper lobe. In the lower lobe, the volcano plot also displayed downregulation of other actin‐binding proteins, such as actin like filament 1 (ALF1) and laminin (LAMA3), in the PVB group. These findings were confirmed by gene set enrichment analysis, which showed mitochondrial membrane protein groups enriched in the PVB in both upper and lower lobes. Our preliminary data argues in favor of investigating the interaction of mitochondrial proteins with ECM and the actin cytoskeleton pathway to elucidate the underlying pathology in development of PH. We aim to adopt a nontargeted metabolomics approach to delve further into in this interaction.

## A052 THE METABOLIC THEORY OF PULMONARY ARTERIAL HYPERTENSION: A NEW BIOMARKER AND A NEW MOUSE MODEL THAT RECAPITULATES THE HUMAN DISEASE BETTER THAN EXISTING MODELS

Yongneng Zhang, Sotirios D Zervopoulos, Aristeidis E Boukouris, Maria Areli Lorenzana‐Carrillo, Bruno Saleme, Linda Webster, Yongsheng Liu, Alois Haromy, Seyed Amirhossein Tabatabaei‐Dakhili, John R Ussher, Gopinath Sutendra, Evangelos D Michelakis

*Department of Medicine (Cardiology), Faculty of Medicine and Dentistry; Faculty of Pharmacy and Pharmaceutical Sciences, University of Alberta, Edmonton, Alberta, Canada*

Isolated loss‐of‐function single nucleotide polymorphisms (SNPs) for SIRT3 (a mitochondrial deacetylase) and UCP2 (an atypical uncoupling protein enabling mitochondrial calcium entry) have been associated with both pulmonary arterial hypertension (PAH) and insulin resistance, but their collective role in animal models and patients is unknown. In a prospective cohort of patients with PAH (*n* = 60), we measured SNPs for both SIRT3 and UCP2, along with several clinical features (including invasive hemodynamic data) and outcomes. We found SIRT3 and UCP2 SNPs often both in the same patient in a homozygous or heterozygous manner, correlating positively with PAH severity and associated with the presence of type 2 diabetes and 10‐year outcomes (death and transplantation). To explore this mechanistically, we generated double knockout mice for *Sirt3* and *Ucp2* and found increasing severity of PAH (mean pulmonary artery pressure, right ventricular hypertrophy/dilation and extensive vascular remodeling, including inflammatory plexogenic lesions, in a gene dose‐dependent manner), along with insulin resistance, compared with wild‐type mice. The suppressed mitochondrial function (decreased respiration, increased mitochondrial membrane potential) in the double knockout pulmonary artery smooth muscle cells was associated with apoptosis resistance and increased proliferation, compared with wild‐type mice. Our work supports the metabolic theory of PAH and shows that these mice exhibit spontaneous severe PAH (without environmental or chemical triggers) that mimics human PAH and might explain the findings in our patient cohort. Our study offers a new mouse model of PAH, with several features of human disease that are typically absent in other PAH mouse models.

## A053 ROLE OF LONG NONCODING RNAS TGFB2‐AS1 AND VIM‐AS1 IN PULMONARY HYPERTENSION

Chanil Valasarajan, Prabhdeep S Ghothra, Christian Mueke, Werner Seeger, Rajkumar Savai, Soni S Pullamsetti

*Max Planck Institute for Heart and Lung Research, Member of the German Center for Lung Research (DZL), Member of the Cardio‐Pulmonary Institute (CPI), Bad Nauheim, D‐61231, Germany; Department of Internal Medicine, Member of the DZL, Member of Cardio‐Pulmonary Institute (CPI), Justus Liebig University, Giessen, 35392, Germany, Department of Internal Medicine, Member of the DZL, Member of Cardio‐Pulmonary Institute (CPI), Justus Liebig University, Giessen, 35392, Germany, Max Planck Institute for Heart and Lung Research, Member of the German Center for Lung Research (DZL), Member of the Cardio‐Pulmonary Institute (CPI), Bad Nauheim, D‐61231, Germany, Max Planck Institute for Heart and Lung Research, Member of the German Center for Lung Research (DZL), Member of the Cardio‐Pulmonary Institute (CPI), Bad Nauheim, D‐61231, Germany; Department of Internal Medicine, Member of the DZL, Member of Cardio‐Pulmonary Institute (CPI), Justus Liebig University, Giessen, 35392, Germany; Institute for Lung Health (ILH), Justus Liebig University, D‐35390, Giessen, Germany*

Dysregulation of various long Noncoding RNAs (lncRNAs) is emerging as a major driver of disease induction and pathology. An imbalance in the TGFbeta and BMP signaling is known to play a crucial role in pathogenesis of pulmonary hypertension. The role of TGFbeta signaling in modulating lncRNAs is less explored in pulmonary hypertension. The aim was to delineate the role of lncRNA TGFb2‐AS1 and VIM‐AS1 in pulmonary hypertension and in disease progression via modulation of TGFbeta signaling. Expression analysis of human pulmonary artery smooth cells (hPASMCs) derived from idiopathic pulmonary arterial hypertension (IPAH) patients and healthy controls showed that TGFb2‐AS1 and VIM‐AS1 expression was upregulated in disease conditions. To delineate the downstream signaling of TGFb2‐AS1 and VIM‐AS1, RNA‐sequencing was performed upon TGFb2‐AS1 and VIM‐AS1 knockdown. It was observed that knockdown of TGFb2‐AS1 and VIM‐AS1 significantly altered the TFGbeta signaling in IPAH PASMCs. Interestingly, from our existing JMJD2b RNA‐seq data set, it was observed that TGFbeta signaling was a major altered signaling pathway upon JMJD2b knockdown. From the same data set, it was observed that TGFB2‐AS1 and VIM‐AS1 expression was downregulated upon JMJD2b knockdown, which was verified by qPCRs. It was also observed that of expression of JMJD2b was upregulated in PASMCs and laser micro‐dissected vessels of IPAH patients compared with healthy controls. Functional studies have shown that knocking down of JMJD2b, TGFb2‐AS1 and VIM‐AS1 using GapmeRs induced a strong proapoptotic and antiproliferative phenotype in IPAH PASMCs. The lncRNAs TGFB2‐AS1 and VIM‐AS1 are regulated by histone demethylase JMJD2b and play a role in induction of the disease phenotype in IPAH PASMCs via modulation of the TGFbeta signaling.

## A054 IMPAIRED FGF10 SIGNALING IS INVOLVED IN THE DEVELOPMENT OF CIGARETTE SMOKE‐INDUCED EMPHYSEMA AND PH

Stefan Hadzic, Cheng‐Yu Wu, Marija Gredic, Oleg Pak, Simone Kraut, Baktybek Kojonazarov, Jochen Wilhelm, Monika Brosien, Mariola Bednorz, Michael Seimetz, Andreas Günther, Djuro Kosanovic, Natascha Sommer, David Warburton, Friedrich Grimminger, Hossein A Ghofrani, Ralph T Schermuly, Werner Seeger, Elie El Agha, Saverio Bellusci, Norbert Weissmann

*Excellence Cluster Cardio‐Pulmonary Institute (CPI), Universities of Giessen and Marburg Lung Center (UGMLC), Member of the German Center for Lung Research (DZL), Justus‐Liebig‐University, Giessen, Germany; Institute for Lung Health (ILH), Excellence Cluster Cardio‐Pulmonary Institute (CPI), Universities of Giessen and Marburg Lung Center (UGMLC), Member of the German Center for Lung Research (DZL), Justus‐Liebig‐University, Giessen, Germany; Sechenov First Moscow State Medical University (Sechenov University), Moscow, Russia; Children's Hospital Los Angeles, Los Angeles, CA, USA; Keck School of Medicine, University of Southern California, Los Angeles, CA, USA; Max‐Planck Institute for Heart and Lung Research, Bad Nauheim, Germany*

Chronic obstructive pulmonary disease (COPD) is an incurable and poorly treatable medical condition characterized by emphysema and chronic bronchitis. In COPD patients, remodelling of the pulmonary vasculature can occur, causing at least mild pulmonary hypertension (PH). Several cellular and molecular mechanisms have been suggested to contribute to disease development, including nitrosative and oxidative stress, augmented inflammation, increased proteolysis, senescence and dysregulated developmental pathways. Fibroblast growth factor (FGF) 10 is an essential player during murine lung morphogenesis, and polymorphisms in the human *FGF10* gene are correlated with increased COPD susceptibility. However, the connection between *FGF10* haploinsufficiency and COPD pathogenesis, in addition to the underlying mechanisms, remain obscure. We found impaired FGF10 signalling in the lung alveolar compartment and in primary interstitial lung fibroblasts of COPD lungs. When exposed to cigarette smoke (CS) extract or nitrosative/oxidative stress, such effects were mimicked in healthy donor lung fibroblasts. Moreover, mice lacking one functional *Fgf10* or FGF receptor 2b (*Fgfr2b)* allele spontaneously developed emphysema, PH and other pathological features that usually arise in wild‐type (Wt) mice upon CS exposure, such as nitrotyrosine formation, increased matrix metalloproteinases (MMPs) activity and apoptosis. Interestingly, chronic CS exposure could not further worsen emphysema and PH phenotype in mice with impaired FGF10 signalling. Transcriptomic analysis performed in laser‐capture microdissected pulmonary vessels and alveolar walls revealed that *Fgf10* ^+/−^ mice have similar gene expression patterns to CS‐exposed Wt mice in both compartments. Overall, our study identified impaired FGF10 signalling as an integral part of the pathomechanisms underlying CS‐induced emphysema and PH development. Of interest, in therapeutic approaches, FGF10 overexpression successfully reversed CS‐ and elastase‐induced emphysema and PH in mice. Therefore, the application of recombinant FGF10 or stimulation of the downstream signalling cascade might represent a therapeutic strategy for treating emphysema and COPD‐associated PH in patients.

## A055 THE EFFECT OF LONG‐TERM OXYGEN THERAPY ON 6‐MINUTE WALKING DISTANCE, CLINICAL PARAMETERS, AND HEMODYNAMICS IN PATIENTS WITH PULMONARY ARTERIAL HYPERTENSION AND CHRONIC THROMBOEMBOLIC PULMONARY HYPERTENSION: A PROSPECTIVE, RANDOMIZED, CONTROLLED TRIAL (SOPHA STUDY)

Panagiota Xanthouli, Ishan Echampati, Satenik Harutyunova, Nicola Benjamin, Christina Alessandra Eichstaedt, Amina Salkić, Benjamin Egenlauf, Rebekka Seeger, Ekkehard Grünig

*Centre for Pulmonary Hypertension, Thoraxklinik at Heidelberg University Hospital, Heidelberg, Germany; Translational Lung Research Center Heidelberg (TLRC), Member of the German Center for Lung Research (DZL), Heidelberg, Germany; Department of Pneumology and Critical Care Medicine, Thoraxklinik University of Heidelberg, Germany; Division of Rheumatology, Department of Internal Medicine V: Hematology, Oncology and Rheumatology, University Hospital Heidelberg, Heidelberg, Germany; Laboratory for Molecular Genetic Diagnostics, Institute of Human Genetics, Heidelberg University, Heidelberg, Germany*

Current recommendations suggest oxygen (O_2_) supplementation in patients with pulmonary hypertension (PH). The effect of long‐term O_2_ administration has not been investigated much among these patients. Thus, the aim of this study is to investigate the effect of long term O_2_‐treatment in patients with pulmonary arterial hypertension (PAH) and chronic thromboembolic pulmonary hypertension (CTEPH) on exercise capacity, clinical parameters and hemodynamics. In this prospective, randomized, controlled trial, 20 patients with PAH or CTEPH under stable PH therapy experiencing oxygen desaturations at rest and/or during physical activity will be randomized to receive oxygen or standard of care (SoC) for 12 weeks. To patients receiving SoC, O_2_ therapy will be offered after 12 weeks (cross‐over design). The primary endpoint is the change in the distance walked in 6 min after 12 weeks of treatment. Secondary endpoints include change in clinical parameters and hemodynamics. Overall, 20 patients (O_2_ *n* = 10 vs. SoC *n* = 10) are planned to participate. So far, 12 patients have already been randomized and nine have completed the study. Two patients died, one patient owing to SARS‐CoV2 pneumonia in the oxygen arm and one owing to right heart failure in the SoC arm. So far, the O_2_ therapy is being well tolerated by all patients. Further results are expected in due course. The study is expected to complete recruitment by the end of September 2022. Oxygen therapy is well tolerated. Further results are expected soon. The effect of long‐term oxygen supplementation should be investigated further in larger controlled trials.

## A056 OXYGENATED HEMOGLOBIN AS PROGNOSTIC MARKER AMONG PATIENTS WITH SYSTEMIC SCLEROSIS SCREENED FOR PULMONARY HYPERTENSION

Panagiota Xanthouli, Ojan Gordjani, Christina Alessandra Eichstaedt, Nicola Benjamin, Franziska Trudzinski, Benjamin Egenlauf, Satenik Harutyunova, Alberto M Marra, Nicklas Milde, Christian Nagel, Norbert Blank, Hanns‐Martin Lorenz, Ekkehard Grünig

*Centre for Pulmonary Hypertension, Thoraxklinik at Heidelberg University Hospital, Heidelberg, Germany; Translational Lung Research Center Heidelberg (TLRC), Member of the German Center for Lung Research (DZL), Heidelberg, Germany; Department of Pneumology and Critical Care Medicine, Thoraxklinik University of Heidelberg, Germany; Division of Rheumatology, Department of Internal Medicine V: Hematology, Oncology and Rheumatology, University Hospital Heidelberg, Heidelberg, Germany; Laboratory for Molecular Genetic Diagnostics, Institute of Human Genetics, Heidelberg University, Heidelberg, Germany; Department of Translational Medical Sciences, “Federico II” University and School of Medicine, Naples, Italy*

Oxygenated hemoglobin (OxyHem) in arterial blood may reflect disease severity in patients with systemic sclerosis (SSc), particularly among those with pulmonary manifestations, such as pulmonary hypertension (PH). Hence, the aim of this study was to analyze the predictive value of OxyHem in SSc patients screened for PH. Systemic sclerosis patients were screened for PH including right heart catheterization, laboratory and clinical assessment. They were followed for 3.2 ± 2.6 (median 3.0) years. Oxygenated hemoglobin was measured by multiplying the concentration of hemoglobin by oxygen saturation assessed in arterialized capillary blood. Kaplan–Meier analysis was performed using the defined threshold from receiver operating characteristic. Prognostic power was compared with known parameters of prognostic significance in SSc using uni‐ and multivariable analysis. Clinical parameters of patients with high and low OxyHem were compared by Student's *t*‐test. A total of 280 SSc patients were screened, and 267 were included in the analysis (82% female, 59.8 ± 13.4 years, 73.8% limited cutaneous SSc); 56 patients had manifest PH and 112 interstitial lung disease (ILD). Low OxyHem ≤12.5 g/dL at baseline was significantly associated with a worse survival (*p* = 0.046) among SSc patients. In the multivariable analysis, the presence of ILD, age ≥60 years and diffusion capacity (DLCO) ≤ 65% were associated with worse survival among SSc patients. The combination of low DLCO and low OxyHem at baseline could predict the development of early pulmonary vascular disease at follow‐up (sensitivity 79.6%). This study detected, for the first time, that an OxyHem level ≤12.5 g/dL is a prognostic predictor in SSc patients. Further studies are needed to confirm these results.

## A057 HYPOCHROMIC ERYTHROCYTES AS PROGNOSTIC INDICATOR OF SURVIVAL AMONG PATIENTS WITH SYSTEMIC SCLEROSIS SCREENED FOR PULMONARY HYPERTENSION

Panagiota Xanthouli, Ojan Gordjani, Christina Alessandra Eichstaedt, Nicola Benjamin, Benjamin Egenlauf, Satenik Harutyunova, Alberto M. Marra, Vivienne Theobald, Nicklas Milde, Christian Nagel, Norbert Blank, Hanns‐Martin Lorenz, Ekkehard Grünig

*Centre for Pulmonary Hypertension, Thoraxklinik at Heidelberg University Hospital, Heidelberg, Germany; Translational Lung Research Center Heidelberg (TLRC), Member of the German Center for Lung Research (DZL), Heidelberg, Germany; Department of Pneumology and Critical Care Medicine, Thoraxklinik University of Heidelberg, Germany; Division of Rheumatology, Department of Internal Medicine V: Hematology, Oncology and Rheumatology, University Hospital Heidelberg, Heidelberg, Germany; Laboratory for Molecular Genetic Diagnostics, Institute of Human Genetics, Heidelberg University, Heidelberg, Germany; Department of Translational Medical Sciences, ‘Federico II’ University and School of Medicine, Naples, Italy*

Iron deficiency is frequent among patients with systemic sclerosis (SSc), particularly among those with pulmonary hypertension (PH). First data indicate prognostic importance of hypochromic erythrocytes (red cells) (HRC) > 2% among patients with PH. Hence, the aim of this study was to investigate the predictive value of HRC in SSc patients screened for PH.

In this retrospective, single‐center cohort study, SSc patients screened for PH were included. Clinical characteristics, laboratory and pulmonary functional parameters associated with the prognosis of SSc were analysed using uni‐ and multivariable analysis. A total of 280 SSc patients were screened, and 171 were included in the analysis, having complete data of iron metabolism (79% female, 61.0 ± 12.9 years of age, 73.2% limited cutaneous SSc, 56 manifest PH and 112 pulmonary fibrosis). The patients were followed for 2.4 ± 1.8 (median 2.4) years. HRC > 2% at baseline was significantly associated with worse survival in the uni‐ (*p* = 0.018) and multivariable analysis (*p* < 0.0001). Overall, 34.5% of the patients suffered from iron deficiency and 22% received iron substitution during follow‐up. HRC > 2% was identified as an independent predictor of mortality for patients with and without pulmonary manifestations of SSc. This study detected, for the first time, that HRC > 2% is an independent prognostic predictor and can possibly be used as a biomarker among SSc patients. Further studies are needed to confirm these results.

## A058 PROGNOSTIC MEANING OF RIGHT VENTRICULAR FUNCTION AND OUTPUT RESERVE IN PATIENTS WITH SYSTEMIC SCLEROSIS

Panagiota Xanthouli, Julia Miazgowski, Nicola Benjamin, Ojan Gordjani, Benjamin Egenlauf, Satenik Harutyunova, Rebekka Seeger, Alberto M. Marra, Norbert Blank, Hanns‐Martin Lorenz, Ekkehard Grünig, Christina Alessandra Eichstaedt

*Centre for Pulmonary Hypertension, Thoraxklinik at Heidelberg University Hospital, Heidelberg, Germany; Translational Lung Research Center Heidelberg (TLRC), Member of the German Center for Lung Research (DZL), Heidelberg, Germany; Department of Pneumology and Critical Care Medicine, Thoraxklinik University of Heidelberg, Germany; Division of Rheumatology, Department of Internal Medicine V: Hematology, Oncology and Rheumatology, University Hospital Heidelberg, Heidelberg, Germany; Department of Translational Medical Sciences, ‘Federico II’ University and School of Medicine, Naples, Italy; Laboratory for Molecular Genetic Diagnostics, Institute of Human Genetics, Heidelberg University, Heidelberg, Germany*

The objective of this study was to investigate the prognostic impact of right ventricular (RV) function at rest and during exercise in patients with systemic sclerosis (SSc) presenting for a screening for pulmonary hypertension (PH). In this study, data from SSc patients who underwent routinely performed examinations for PH screening, including echocardiography and right heart catheterization, at rest and during exercise were analyzed. Uni‐ and multivariable analyses were performed to identify prognostic parameters. Out of 280 SSc patients screened for PH, 225 were included in the analysis (81.3% female, mean age 58.1 ± 13.0 years, 68% limited cutaneous SSc, World Health Organization functional class II–III 74%, 24 manifest PH). During the observation period of 3.2 ± 2.7 (median 2.6) years, 35 patients died. Tricuspid annular plane systolic excursion at rest ≤18 mm (*p* = 0.0004), RV output reserve as an increase of cardiac index (CI) during exercise ≤2 L/min (*p* = 0.0002), RV pulmonary vascular reserve (Δmean pulmonary artery pressure/Δcardiac output) >3 mmHg/L/min (*p* = 0.001), peak CI ≤ 5.5 L/min (*p* = 0.01), pulmonary arterial compliance >2 mL/mmHg (*p* = 0.0005) and echocardiographic qualitative RV function at rest (*p* < 0.0001) significantly predicted survival. In the multivariable analysis, RV function at rest, diffusion capacity for carbon monoxide ≤65% and CI increase <2 L/min/m^2^ were identified as independent prognostic predictors and had >70% sensitivity to predict future development of pulmonary vascular disease (PVD) during follow‐up. This study demonstrates that assessment of RV function at rest and during exercise might provide crucial information to identify SSc patients who are at a high risk of poor outcome and for the development of PH and/or PVD.

## A059 INCIDENCE AND OUTCOMES OF COVID‐19 IN PATIENTS WITH PULMONARY ARTERIAL HYPERTENSION AND CHRONIC THROMBOEMBOLIC PULMONARY HYPERTENSION: DATA FROM THE HELLENIC PULMONARY HYPERTENSION REGISTRY (HOPE)

Ioannis T Farmakis, Panagiotis Karyofyllis, Frantzeska Frantzeskaki, Eftychia Demerouti, Anastasia Anthi, Alexandra Arvanitaki, Georgia Pitsiou, Katerina K Naka, Aris Bechlioulis, Adina Thomaidi, Aikaterini Avgeropoulou, Styliani Brili, Ioanna Mitrouska, Athanasios Manginas, Stylianos E Orfanos, Iraklis Tsangaris, George Giannakoulas

*Cardiology Department, AHEPA University Hospital, Aristotle University of Thessaloniki; Cardiology‐Pediatric Cardiology Department, Onassis Cardiac Surgery Center, Athens; Multidisciplinary Pulmonary Hypertension Center, Attikon University General Hospital, National and Kapodistrian University of Athens Medical School, Athens; 1st Department of Critical Care, National & Kapodistrian University of Athens Medical School, Pulmonary Hypertension Center, Evangelismos General Hospital, Athens, Greece; Adult Congenital Heart Centre and National Centre for Pulmonary Hypertension, Royal Brompton Hospital and National Heart and Lung Institute, Imperial College, London, UK; Respiratory Failure Unit, ‘G. Papanikolaou’ Hospital, Thessaloniki; 2nd Department of Cardiology, University of Ioannina Medical School, University Hospital of Ioannina, Ioannina; Cardiology Department, Democritus University of Thrace, Alexandroupolis; Cardiology Department, Hippokration General Hospital, Athens; Department of Thoracic Medicine, University Hospital of Heraklion, Heraklion; Interventional Cardiology and Cardiology Department, Mediterraneo Hospital, Athens, Greece*

Few reports on coronavirus disease 2019 (COVID‐19) infection in patients with group 1 or group 4 pulmonary hypertension (PH) have been published, with a discrepancy in the incidence of the disease in this rare and heterogeneous population and also differences in the reported outcomes, such as hospitalization and mortality rates. The aim was to describe the case incidence of COVID‐19 among Greek PH expert centers. A total of nine PH expert centers participated in this report, cumulatively caring for 499 PH patients [372 patients with pulmonary arterial hypertension (PAH) and 127 patients with chronic thromboembolic pulmonary hypertension (CTEPH)] according to recent data retrieved from the prospective HOPE Registry and personal communication with the expert centers, to record RT‐PCR‐confirmed COVID‐19 cases. Eighteen cases of COVID‐19 (12 PAH and six CTEPH) were reported from the end of February 2020 to 14 August 2021, contributing to an estimated incidence of 36.1 (95% confidence interval 21.5–56.4) COVID‐19 cases in 1,000 patients with group 1 and 4 PH. The median age of affected patients was 54.5 years (range 25–86 years), and 77.8% were women. All patients reported a symptomatic course of COVID‐19 [median duration 6 days (range 2–30 days)]. No case of incident venous thromboembolism was described. All patients resumed PAH‐targeted therapies during the course of the infection. Eight (44.4%) patients were hospitalized, and four (22.2%) died. Three of them were ≥70 years of age, and the other had significant comorbidities. The remaining four were discharged after a median of 11 days (range 8–30 days). Recovered patients do not report deterioration of their functional status after COVID‐19 infection, nor persistent symptoms. There is increased severity of COVID‐19 among PAH and CTEPH patients, mainly owing to the increased age and comorbidities of the patients. Strong medical advice should be offered to all PH group 1 and 4 patients to be vaccinated and continue to adhere to preventive infection measures.

## A060 A NOVEL REDOX‐SWITCH IN CYCLIN d‐CDK4 REGULATES PULMONARY VASCULAR CELL PROLIFERATION

Hannah Knight, Giancarlo Abis, Manpreet Kaur, Lan Zhao, Clemens Ruppert, Astrid Weiss, Ralph T Schermuly, Philip Eaton, Olena Rudyk

*School of Cardiovascular Medicine & Sciences, British Heart Foundation Centre of Research Excellence, King's College London, London, UK; Division of Biosciences, Institute of Structural and Molecular Biology, University College London, London, UK; Department of Medicine, Faculty of Medicine, Imperial College London, London, UK; Universities of Giessen & Marburg Lung Center Giessen Biobank, Justus‐Liebig‐University Giessen, Giessen, Germany; Department of Internal Medicine, Justus‐Liebig‐University Giessen, Giessen, Member of the German Center for Lung Research (DZL), Germany; William Harvey Research Institute, Barts & The London School of Medicine & Dentistry, Queen Mary University of London, UK*

Pulmonary vascular remodeling is caused, in part, by hyperproliferation of pulmonary smooth muscle cells. Current treatments for pulmonary hypertension (PH) provide only modest improvements to patients. Recent reports show that targeting the cell cycle using cyclin dependent kinase (CDK) inhibitors reverses disease progression in experimental PH. Interestingly, PH is associated with a perturbed redox environment owing to expressional changes of NADPH oxidase and superoxide dismutase, among others. Oxidants, including H_2_O_2_, cause cell cycle arrest by regulating proteins that contain a redox‐active cysteine; however, many of these redox switches remain elusive. Here, we provide evidence for a novel kinase‐inhibitory disulfide bond in cyclin d‐CDK4, which forms in human pulmonary arterial smooth muscle cells in vitro and in pulmonary arteries *in vivo*. By using cysteine mutants and in vitro kinase activity assays, we identified that this reversible disulfide bond forms between specific cysteines in cyclin d‐CDK4 to inhibit phosphorylation of the tumor suppressor protein, retinoblastoma, and induce cell cycle arrest. Importantly, we found that this disulfide bond forms at a critical functionally relevant cysteine, meaning that its mutation is sufficient to impair kinase activity and attenuate cell proliferation. It was found that the abundance of cyclin d‐CDK4 disulfide is decreased in pulmonary arteries of PAH patients, which might account, at least in part, for the previously reported hyperactivity of CDK4 in PH. Next, we investigated whether this kinase‐inhibitory cyclin d‐CDK4 disulfide offers protection in experimental PH, by using a pharmacological thioredoxin reductase inhibitor to potentiate accumulation of the disulfide. We found that this approach attenuated hemodynamic measures of disease severity and decreased vascular remodeling in experimental PH, as measured by immunohistochemical staining of α‐smooth muscle actin. To conclude, a redox switch in cyclin d‐CDK4 represents a novel regulatory site that might provide the opportunity to target this kinase complex selectively using covalent inhibitors to attenuate disease severity in PH.

## A061 ROLE OF HISTONE DEACETYLASE 9 IN THE PATHOGENESIS OF CHRONIC OBSTRUCTIVE PULMONARY DISEASE‐ASSOCIATED PULMONARY HYPERTENSION

Edibe Avci, Siavash Mansouri, Elisabetta Gamen, Sven Zukunft, Ingrid Fleming, Andreas Weigert, Miguel A Alejandre Alcázar, Werner Seeger, Rajkumar Savai, Soni Pullamsetti

*Max Planck Institute for Heart and Lung Research, Parkstraße 1, Bad Nauheim; Metabolomics Core Facility Cardio‐Pulmonary Institute, Goethe‐Universität Frankfurt, Theodor‐Stern‐Kai 7, 60590 Frankfurt am Main; Institute of Biochemistry I, Faculty of Medicine, Goethe‐Universität Frankfurt, Theodor‐Stern‐Kai 7, Frankfurt am Main; Center for Infection and Genomics of the Lung, JLU Giessen, Aulweg 130, Giessen and CECAD Excellent in Aging Research, Uniklinik Köln, Kerpener Straße 62, Köln, Germany*

Histone deacetylase 9 (HDAC9), a member of class IIa, has been shown to play roles in regulation of gene expression in tumor formation, inflammation, cardiac hypertrophy, atherosclerosis and metabolic diseases, such as obesity. However, the regulation of HDAC9 in the aging‐associated lung changes and pulmonary hypertension (PH) remains to be explored. In this study, we used three age groups [group 1, 8–12 weeks old (young); group 2, 30–34 weeks old (middle aged); and group 3, ≥48 weeks old (aged)]. Aged *HDAC9* knockout (KO) animals exhibited reduced survival rate and decreased body weight when compared with matching aged wild‐type (WT) animals. Histological assessment of *HDAC9* KO mouse lungs displayed reduced alveolarization and thickening of the pulmonary vessels (increased muscularization and medial wall thickness), hinting at chronic obstructive pulmonary disease‐associated PH (COPD‐PH) owing to the loss of *HDAC9* in aged mice. Following pulmonary inflammation, structures known as inducible bronchus‐associated lymphoid tissue (iBALT) were observed close to remodelled pulmonary vasculature. To characterize the inflammatory lungs, Hematoxylin and Eosin staining and fluorescence‐activated cell sorting (FACS) analysis were performed in the three age groups. Interestingly, FACS analysis revealed that fibroblast, vascular smooth muscle cell and endothelial cell abundance increased significantly in aged *HDAC9* KO total lungs. CD45^+^/CD8^+^ T‐cell abundance increased significantly, as did the CD8^+^/CD4^+^ ratio, which indicates chronic adaptive inflammation along with iBALT formation. Hematoxylin and Eosin staining confirmed the recruitment of CD45^+^/CD3^+^ T cells to inflamed compartments of the lungs in aged *HDAC9* KO mice. Notably, HDAC9 is the only member of the class IIa HDACs to bbe downregulated in the lung tissues of both COPD and COPD‐PH patients. HDAC9 is the crucial molecule driving COPD and COPD‐PH via modulation of inflammatory pathways.

## A062 MICROFLUIDIC MODEL OF PULMONARY ARTERIAL HYPERTENSION—A NEW PLATFORM FOR DRUG TESTING?

Alexander J Ainscough, Maike Haensel, Timothy J Smith, Christopher J Rhodes, Adam Fellows, Luke S Howard, John Wharton, Martin R Wilkins, Joshua B Edel, Beata Wojciak‐Stothard

*National Heart and Lung Institute, Imperial College London, London, UK; Department of Chemistry, Imperial College London, London, UK*

Pulmonary arterial hypertension (PAH) is an unmet clinical need. The disease is triggered by endothelial damage, followed by exaggerated repair involving increased proliferation and resistance to apoptosis of cells in the arterial intimal and medial layers. Loss of function of bone morphogenetic protein receptor 2 (BMPR2) in pulmonary endothelium predisposes to PAH. The lack of a disease model representative of the human condition is a key obstacle to the development of new treatments. Here, we present an inducible ‘two hit’ microfluidic model of PAH, based on a biomimetic pulmonary artery (PA)‐on‐a‐chip, that permits the study of the molecular and functional changes in human pulmonary vascular endothelial and smooth muscle cells in response to triggers of the disease and their response to drugs. The device, produced with computer microchip technologies, consists of two channels accommodating human pulmonary vascular endothelial cells (HPAECs) cocultured under flow with human pulmonary vascular smooth muscle cells (HPASMCs). Natural or induced endothelial BMPR2 dysfunction combined with hypoxia trigger PASMC proliferation, which is responsive to treatment with imatinib and ambrisentan. Changes in gene expression in HPAECs, blood derived endothelial cells from patients with *BMPR2* mutations and HPASMCs are consistent with observations made in genomic and biochemical studies of the human disease and enable insights into underlying disease pathways and mechanisms of drug response. This inducible disease model offers a novel, promising and more easily accessible approach for researchers to study pulmonary vascular remodelling and advance drug development in PAH.

## A063 IRG1/ITACONATE AXIS RE‐ORCHESTRATES IMMUNE CELL PHENOTYPE AND ACTIVATION IN PULMONARY HYPERTENSION

Golnaz Hesami, Siavash Mansouri, Chanil Valasarajan, Andreas Weigert, Baktybek Kojonazarov, Werner Seeger, Rajkumar Savai, Soni S Pullamsetti

*Department of Lung Development and Remodeling, Max‐Planck Institute for Heart and Lung Research, Bad Nauheim; Institute of Biochemistry I, Goethe‐University Frankfurt, Frankfurt; Max‐Planck Institute for Heart and Lung Research, Bad Nauheim, Germany*

Pulmonary hypertension (PH) is a severe, progressive and lethal cardiopulmonary disorder characterized by remodeling of the pulmonary vascular wall and elevated mean pulmonary arterial pressure. Bone marrow (BM)‐derived hematopoietic lineage cells, particularly myeloid cells, can contribute to the process of pulmonary vascular remodeling. Immune‐responsive gene 1 (IRG1) encodes the *cis‐aconitate* decarboxylase enzyme that produces itaconate in inflammatory conditions in myeloid lineage cells, principally in macrophages. However, the association of the IRG1/itaconate axis with vascular remodeling and PH progression is largely unknown. Here, we reveal that hypoxic exposure of *Irg1* ^−/−^ mice, in addition to adoptive transfer of bone marrow (BM) from Irg1‐deficient mice into naïve wild‐type (WT) mice, exacerbates hypoxia‐induced pulmonary vascular remodeling, PH and right ventricular dysfunction. Furthermore, flow cytometry immune profiling showed that *Irg1*‐deficient mice exhibit altered immune cell populations in both lung and BM; especially, infiltration of macrophages, dendritic cells and natural killer cells into the lungs of *Irg1* ^−/−^ mice upon hypoxia. Moreover, *Irg1* depletion can dysregulate activation of BM‐derived macrophages. *In vitro* studies demonstrated that treatment with the IRG1‐dependent metabolite 4‐octyl itaconate (4‐OI) reduces the proliferation of human pulmonary artery smooth muscle cells and pulmonary artery adventitial fibroblasts. Furthermore, IRG1 is upregulated in pulmonary artery smooth muscle cells upon inflammatory stimulation, and silencing IRG1 in these cells results in a hyper‐proliferative phenotype, while IRG1 overexpression significantly reduces the growth rate of the cells. Notably, the immunomodulatory effect of itaconate was translated in vivo, where administration of 4‐octyl itaconate 4‐OI in a therapeutic approach attenuated monocrotaline‐induced PH in rats. Taken together, this study demonstrates that the IRG1/itaconate axis can play a regulatory role in the development of PH through an immune–metabolic reprogramming of the phenotype and activation of immune cells.

## A064 NONINVASIVE PREDICTORS OF PULMONARY ARTERIAL HYPERTENSION IN INTERSTITIAL LUNG DISEASE

Stella M. Savarimuthu, Phillip Joseph, Marjorie Cullinan, Paul M Heerdt, Inderjit Singh

*Division of Pulmonary, Critical Care and Sleep Medicine, Department of Medicine, Yale School of Medicine, New Haven, CT; Department of Respiratory Care, Yale New Haven Hospital, New Haven, CT; Department of Anesthesiology, Division of Applied Hemodynamics, Yale New Haven Hospital and Yale School of Medicine, New Haven, CT, USA*

Pulmonary arterial hypertension (PAH) in interstitial lung disease (ILD) is associated with increased mortality, reduced quality of life and impaired exertional capacity. Right heart catheterization (RHC) is the diagnostic gold standard for PAH but is invasive and not readily available. Noninvasive physiologic evaluation might be predictive of PAH in ILD. We conducted a retrospective analysis of patients with ILD who underwent 6‐min walk testing (6MWT), pulmonary function testing (PFT), submaximum cardiopulmonary exercise testing (CPET) and RHC between January 2019 and January 2022. Thirty‐eight patients with ILD and PAH diagnosed on RHC (PAH‐ILD) were compared with 16 age‐ and sex‐matched control patients with ILD and without PAH. Area under the receiver‐operating characteristic curve (AUC) analysis was performed on variables from submaximum CPET, 6MWT, PFT and echocardiogram to characterize ILD patients with and without PAH. Univariate and multivariate analyses were performed to determine predictors of extrapolated maximum oxygen uptake (VO_2max_) on submaximum CPET. Six‐minute walk distance (6MWD) and diffusing capacity for carbon monoxide (DLCO) were lower in patients with PAH‐ILD. On submaximum CPET, PAH‐ILD patients exhibited a lower gas exchange‐derived pulmonary vascular capacitance (GXCAP) and lower submaximum and extrapolated VO_2max_. On univariate analysis, primary independent predictors of extrapolated VO_2max_ among all ILD patients were DLCO [β‐coefficient: 12.16, 95% confidence interval (CI): 3.54–20.79, *p* < 0.01], 6MWD (β‐coefficient: 21.47, 95% CI: 11.66–31.29, *p* < 0.0001) and GXCAP (β‐coefficient: 19.38, 95% CI: 11.86–26.89, *p* < 0.0001). Only GXCAP predicted extrapolated maximum VO_2_ on multivariate analysis (β‐coefficient: 16.62, CI: 2.61–30.64, *p* < 0.02). Among all noninvasive variables, GXCAP was the strongest predictor of PAH‐ILD (AUC 0.78, CI: 0.65–0.90). The GXCAP obtained from submaximum CPET was most predictive of PAH‐ILD compared with noninvasive measurements from 6MWT, PFT and echocardiogram. Submaximum CPET might allow for earlier identification of PAH in ILD.

## A065 PROGNOSTIC IMPACT OF HYPOCHROMIC ERYTHROCYTES IN PATIENTS WITH PULMONARY ARTERIAL HYPERTENSION

Christina A Eichstaedt, Panagiota Xanthouli, Vivienne Theobald, Nicola Benjamin, Alberto M Marra, Anna D'Agostino, Benjamin Egenlauf, Memoona Shaukat, Ding Cao, Antonio Cittadini, Eduardo Bossone, Maria Kögler, Martina Muckenthaler, Ekkehard Grünig

*Center for Pulmonary Hypertension, Thoraxklinik Heidelberg gGmbH at Heidelberg University Hospital; Translational Lung Research Center Heidelberg (TLRC) at the German Center for Lung Research (DZL), Laboratory for Molecular Diagnostics, Institute of Human Genetics, Heidelberg University, Heidelberg, Germany; Department of Translational Medical Sciences, ‘Federico II’ University Hospital and School of Medicine, ‘Federico II’ University, Naples; IRCCS SDN, Naples; Department of Cardiology, Division of Cardiac Rehabilitation‑Echo Lab Antonio Carderelli Hospital, Naples, Italy; Department of Pediatric Oncology, Hematology, Immunology and Pulmonology, Molecular Medicine Partnership Unit (MMPU) Group Leader, University Hospital Heidelberg, Germany*

Iron deficiency affects ≤50% of patients with pulmonary arterial hypertension (PAH), but iron markers, such as ferritin and serum iron, are confounded by several non‐disease‐related factors, such as acute inflammation and diet. The aim of this study was to identify a new marker for iron deficiency and clinical outcomes in PAH patients. In this single‐center, retrospective study, we assessed indicators of iron status and their impact on time to clinical worsening (TTCW) and survival in PAH patients at the time of initial diagnosis and at 1‐year follow‐up using univariable and multivariable analysis. In total, 150 patients were included with an invasively confirmed PAH and complete data on iron metabolism. The proportion of hypochromic erythrocytes >2% at initial diagnosis was identified as an independent predictor for a shorter TTCW (*p* = 0.0001) and worse survival (*p* = 0.002), in addition to worse survival (*p* = 0.016) at 1‐year follow‐up. Only a subset of these patients (64%) suffered from iron deficiency defined by serum iron levels <12 g/dL in women and <13 g/dL in men. Low ferritin or low serum iron was not correlated with TTCW or survival. Severe hemoglobin deficiency at baseline was significantly associated with a shorter TTCW (*p* = 0.001). The presence of hypochromic erythrocytes >2% was a strong and independent predictor of mortality and shorter TTCW in this cohort of PAH patients. Thus, it can serve as a valuable indicator of iron homeostasis and prognosis even in patients without iron deficiency or anemia. Further studies are needed to confirm the results and to investigate therapeutic implications.

## A066 NON‐CARDIOVASCULAR, NONOBSTETRIC SURGERY IN PRECAPILLARY PULMONARY HYPERTENSION AT A SINGLE CENTER IN JAPAN

Yoichi Sugiyama, Hiroto Shimokawahara, Ayane Miyagi, Takeshi Suetomi, Aiko Ogaya, Hiromi Matsubara

*National Hospital Organization Okayama Medical Center, Japan*

Recently, the survival outcome of precapillary pulmonary hypertension (pc‐PH) has been remarkably improved, with the progress of treatment options. Hence, the number of non‐cardiovascular, non‐obstetric surgeries for patients with pc‐PH will be expected to increase. The perioperative clinical outcome from Western countries has been reported, but data from Asia have not been described. From January 2008 to December 2020, a total of 33 pc‐PH cases (82% female, mean age 53 years) underwent the initial non‐cardiovascular, non‐obstetric surgeries with general anesthesia at National Hospital Organization Okayama Medical Center. We retrospectively assessed variables associated with perioperative complications using binary logistic analysis. Perioperative complications were defined as heart failure, respiratory failure, renal failure, hepatic failure and sepsis. At the time of surgery, 17 cases (52%) were in World Health Organization functional class III/IV, and 16 cases (49%) were treated with parenteral prostacyclin. Preoperative mean pulmonary arterial pressure >40 mmHg was in nine cases (27%). Emergency surgery was performed in six cases (18%). Preoperative use of catecholamines was undertaken in seven cases (21%). Perioperative complications occurred in seven cases (21%), of whom one (3%) died. The predictive factor for perioperative complications was preoperative mean pulmonary arterial pressure using multivariate regression analysis (*p* < 0.05), of which the cut‐off value was 31 mmHg from the receiver operating characteristic curve (area under the curve: 0.920; sensitivity: 1.000; specificity: 0.731). Mean pulmonary arterial pressure was significantly associated with perioperative complications. Careful perioperative management would be recommended for pc‐PH with high mean pulmonary arterial pressure.

## A067 ATP CITRATE LYASE (ACLY), A NEW THERAPEUTIC TARGET FOR VASCULAR REMODELING DISEASES

Yann Grobs, Charlotte Romanet, Alice Bourgeois, Sarah‐eve Lemay, Tsukasa Shimauchi, Sandra Breuils Bonnet, François Potus, Steeve Provencher, Olivier Boucherat, Sebastien Bonnet

*IUCPQ, Quebec, QC; CRIUCPQ, Quebec, QC; Laval University, Quebec, QC, IUCPQ Research Center, Quebec, QC, Canada; Quebec Heart and Lung institute, Quebec, QC, Canada*

Pulmonary arterial hypertension (PAH) and coronary arterial disease (CAD) are two vascular remodeling diseases (VRD) characterized by a cancer‐like pro‐proliferative and apoptosis‐resistant phenotype of smooth muscle cells (SMCs), fueled by a metabolic shift toward glycolysis and interconnected global changes in the epigenetic landscape. The nucleo‐cytoplasmic enzyme ATP citrate lyase (ACLY) has recently emerged as a key player and therapeutic target in cancer by favoring the Warburg effect, lipid synthesis and chromatin remodeling. However, its role in VRD is unknown. We hypothesized that ACLY is upregulated in VRD and supports the abnormal phenotype of VRD‐SMCs. Increased expression and nuclear localization of ACLY were observed in distal pulmonary artery (PA), isolated pulmonary artery SMCs (PASMCs) from PAH patients and in coronary arteries (CoAs) and CoASMCs of CAD patients compared with controls (immunoblot and immunofluorescence) and in PAH& CAD animal models (Sugen/hypoxia and carotid injury). We found that inhibition of ACLY impedes PAH‐PASMC bioenergetics (TMRM and seahorse assessment) and decreased histone H3 and H4 acetylation leading to reduced PAH‐PASMC proliferation and survival (Ki67 and Annexin V labeling, PCNA, Survivin, PLK1 and STAT3 immunoblots). *Ex vivo*, ACLY inhibition in CoAs and CoASMCs of CAD patients reverses the proliferative and antiapoptotic phenotype. *In vivo*, pharmacological inhibition of ACLY in Sugen/hypoxia‐challenged rats with established PAH significantly improved pulmonary hemodynamics (RVSP, mPAP and TAPSE) and RV function (stroke volume and cardiac output), as determined by echocardiography and right heart catheterization. Consistently, pulmonary vascular remodeling (EVG) of distal PAs was reduced by ACLY inhibition. In agreement with this, we found that inactivation of *Acly* targeted to smooth muscle cells confers protection in a gene dosage‐dependent manner against Sugen/hypoxia‐induced PAH in mice. Inhibition of ACLY also resulted in an anti‐remodeling effect in a rat model of carotid injury. In conclusion, we demonstrated that inhibition of ACLY might represent a novel and attractive therapeutic avenue to correct VRD.

## A068 THE EFFECT OF MITOTEMPO ON REGULATION OF HYPOXIA‐INDUCIBLE FACTOR‐1α AND DEVELOPMENT OF HYPOXIA‐INDUCED PULMONARY HYPERTENSION

Esraa M Zeidan, Ashraf Taye, Mohamed M A Khalifa, Oleg Pak, Anika Nolte, Hossein A Ghofrani, Werner Seeger, Norbert Weissmann, Natascha Sommer, Claudio Nardiello

*Excellence Cluster Cardio‐Pulmonary Institute (CPI), University of Giessen and Marburg Lung Center (UGMLC), Member of the German Center for Lung Research (DZL), Justus‐Liebig‐University, Giessen, Germany; Department of Pharmacology and Toxicology, Faculty of Pharmacy, Minia University, El‐Minia; Department of Pharmacology and Toxicology, Faculty of Pharmacy, South Valley University, Egypt*

Pulmonary hypertension (PH) is an incurable, progressive disease, characterized by pulmonary arterial remodeling that can subsequently culminate in right heart failure and premature death. The hypoxia‐inducible factor‐1 (HIF1) orchestrates the physiological response to hypoxia and plays a fundamental role in the pathogenesis of PH. Mitochondria regulate hypoxic sensing and signaling, however, the exact role of mitochondrial reactive oxygen species (ROS) for stabilization of HIF1α in pulmonary arterial smooth muscle cells (PASMCs) and development of PH remains unclear. Stabilization of HIF1α was determined at different time points and degrees of hypoxic exposure and in different cell types [mouse lung adenocarcinoma cell line (CMT167) and human pulmonary arterial smooth muscle cells (hPASMCs)] by western blot. Protein expression was measured in the presence and absence of the mitochondrial superoxide dismutase mimetic MitoTempo. Chronic hypoxia‐induced PH was quantified in mice exposed to normobaric hypoxia (10% O_2_) for 4 weeks by in vivo hemodynamic measurement and echocardiography analysis after treatment with MitoTempo or its inactive carrier, triphenylphosphonium (TPP). HIF1α was upregulated after hypoxic incubation with 1% O_2_ for 24 and 48 h in both CMT167 cells and hPASMCs. Interestingly, the metabolic parameters, pyruvate dehydrogenase kinase‐1 (Pdk1) and lactate dehydrogenase A (Ldha), were upregulated in CMT167 cells after 24 h, 48 h and 5 days of hypoxia (1% O_2_), but we could not detect upregulation of Pdk1 in hPASMCs. Application of MitoTempo did not change HIF1α expression in vitro or the chronic hypoxia‐induced PH parameters in vivo in comparison to treatment with TPP. HIF1α protein level was regulated in a time‐ and cell type‐specific manner. Mitochondrial ROS inhibition by MitoTempo treatment did not affect HIF1α stabilization in vitro or development of hypoxia‐induced PH in vivo.

## A069 ENDOBRONCHIAL AEROSOLIZED AAV1.SERCA2A GENE THERAPY FOR TREATING PULMONARY HYPERTENSION IN A PIG MODEL: THERAPEUTIC EFFICACY AND DIRECT COMPARISON WITH THE INTRATRACHEAL AEROSOLIZATION IN PH PIGS

Olympia Bikou, Serena Tharakan, Taro Kariya, Kelly Yamada, Roger Hajjar, Kiyotake Ishikawa

*Cardiovascular Research Institute, Icahn School of Medicine at Mount Sinai, New York, NY, USA; Klinikum rechts der Isar, Technical University of Munich, Munich, Germany; Phospholamban Foundation, Amsterdam, The Netherlands*

Gene therapy has the ability to target the roots of pulmonary hypertension (PH) and holds evident promise. However, two bottlenecks are currently encountered in the gene therapy for PH: (1) lack of an efficient delivery mode; and (2) lack of large animal models mimicking the human condition. The differences in lung anatomy and (patho)physiology between humans and rodents render the pulmonary application of new gene therapeutic approaches in large animals inevitable. The goals of this study were: (1) to test the safety and efficacy of sarcoplasmic/endoplasmic reticulum Ca^2+^‐ATPase 2a (SERCA2a) gene therapy using an adeno‐associated virus as vector (AAV1. SERCA2a) in a pig PH model; and (2) to identify the most efficient delivery mode for PH in‐lung gene therapy. In part one, PH was induced in Yorkshire pigs by surgically binding the pulmonary veins. Two months after surgery, the animals were randomized, and 1 × 10^13^ AAV1. SERCA2a or saline was aerosolized in the distal bronchi using a sprayer inserted in a flexible bronchoscope. We hypothesized that targeted delivery would increase viral uptake and avoid virus loss in off‐target compartments. The animals were observed for a further 2 months. High viral genomes were detected in the AAV1. SERCA2a‐treated lung parts. This was accompanied by functional and morphometrical amelioration of PH. In part two, the efficiency of the endobronchial aerosolization was directly compared with another in‐lung delivery mode from a previous study: intra‐tracheal aerosolization. These pigs underwent the same experimental protocol apart from the delivery mode: AAV1. SERCA2a was aerosolized using a stiff metal sprayer inserted through the endotracheal tube. The virus was aerosolized before the carina. Endobronchial delivery led to higher viral expression (6.719 ± 927 vs. 1.444 ± 402 vgc/100 ng DNA, *p* = 0.0017). These results indicate that endobronchial aerosolization is a promising delivery method for translating PH gene therapy to the clinic.

## A070 INVESTIGATION OF EFFICACY, SAFETY AND OPTIMAL DOSE OF CS1 IN SUBJECTS WITH PULMONARY ARTERIAL HYPERTENSION: A PROSPECTIVE, RANDOMIZED, MULTICENTER, PARALLEL‐GROUP PHASE 2 STUDY

Raymond Benza, Niklas Bergh, Philip Adamson, Björn Dahlöf

*Wexner Medical Center, Ohio State University, OH, USA; Institute of Medicine, University of Gothenburg, Gothenburg, Sweden; Cereno Scientific, Gothenburg, Sweden; Abbott Inc., Austin, TX, USA*

Pulmonary arterial hypertension (PAH) is a fatal rare disease, which currently has no disease‐modifying therapy available to target the underlying pathogenesis. CS1 (from Cereno Scientific) is a controlled‐release formulation of valproic acid (VPA), with a putative fourfold mechanism of action: antifibrotic, antithrombotic, anti‐inflammatory and PAH pressure reducing, mediated, in part, by epigenetic modulation via histone deacetylase inhibition (HDACi). In this phase 2a trial (NCT05224531), patients with New York Heart Association/World Health Organization functional class II or III, PAH class I (male and female, aged 18–80 years, body mass index 18‐40 kg/m^2^) with limited exercise capacity, on stable monotherapy or dual combination therapy and at intermediate/high risk (REVEAL Risk Score 2.0) of clinical worsening will be randomized single‐blind to one of three daily oral doses of CS1 (480, 960 or 1,920 mg), 6 weeks after right heart catheterization (RHC) and implantation of the CardioMEMS (Abbott) pulmonary artery sensor to enable ambulatory hemodynamic monitoring over the following 12 weeks of CS1 treatment. The primary endpoint is safety and tolerability, measured by adverse events, adverse device effects, bleeding, changes in vital signs and abnormalities in laboratory and ECG parameters. Secondary endpoints include changes from baseline to end of treatment in: mean pulmonary artery pressure and other CardioMEMS hemodynamic measures, cardiac MRI and echocardiogram parameters (both blindly read at core centre), RHC parameters, PAH disease management and risk scores (REVEAL 2.0 and REVEAL Lite 2), quality of life using the PAH‐Symptoms and Impact Questionnaire scale and the Minnesota Living with Heart Failure Questionnaire, 6‐min walk distance, brain natriuretic peptide levels (BNP and N‐terminal pro‐BNP), plasminogen activator inhibitor‐1 levels and actigraphy. Pre‐ and post‐dose plasma concentrations of VPA at visits 4, 5 and 9 (end of treatment) will also be analysed. The one or two most efficacious (multiparameter assessment), safe and well‐tolerated doses will be selected for the phase 2b/3 study.

## A071 HEMODYNAMIC REMISSION INDUCTION BY INTRAVENOUS PROSTACYCLIN IN SEVERE PULMONARY ARTERIAL HYPERTENSION

Takeshi Suetomi, Hiroto Shimokawahara, Yoichi Sugiyama, Ayane Miyagi, Aiko Ogawa, Mari Nishizaki, Hiromi Matsubara

*National Hospital Organization Okayama Medical Center, Okayama, Japan*

Intravenous prostacyclin (PGI_2_) is the most effective treatment for severe pulmonary arterial hypertension (PAH). We had previously reported that rapid uptitration of PGI_2_ significantly decreased mean pulmonary artery pressure (mPAP) and improved long‐term survival of the patients. As a next step, we have been aiming for hemodynamic remission of severe PAH patients by dual or triple combination therapy including intravenous PGI_2_ with more rapid uptitration. Thirty‐five consecutive patients with severe PAH who initiated intravenous PGI_2_ were analyzed retrospectively. The rapid uptitrate group was defined as those receiving PGI_2_ > 20 ng/kg/min within 1 month and >40 ng/kg/min within 3 months after the initiation. Hemodynamic remission was defined as mPAP ≤ 20 mmHg and PVR < 3 Wood units. At 1‐year follow‐up, the hemodynamic remission was achieved in 10 of 19 cases in the rapid up‐titrate group, but none of 16 cases in the control group (53 vs. 0%, *p* < 0.001). In the rapid up‐titrate group, patients who commenced PGI_2_ within 1 month after oral medications had a higher remission rate with a lower dose of PGI_2_ at 1 year compared with the others (hemodynamic remission rate 67 vs. 29%, average PGI_2_ dose 55.3 ± 15.8 vs. 72.5 ± 17.0 ng/kg/min, *p* = 0.019). In PAH patients, upfront combination use of intravenous PGI_2_ with rapid uptitration resulted in a high probability of achieving hemodynamic remission with a smaller dose of PGI_2_ and would be expected to improve long‐term outcome.

## A072 PULMONARY HYPERTENSION MEDICATIONS IN SPECIAL POPULATIONS: RENAL AND LIVER DYSFUNCTION

Franck F Rahaghi, Samantha Gillenwater, Starlet Harrimon, Teresa Palacios, Phelen McCoy, Jinesh Mehta

*Cleveland Clinic Florida, Weston, FL, USA*

Pulmonary hypertension medications, typically directed to remedy increased pulmonary vascular resistance, have a variety of formulations and are affected differently by renal and liver insufficiency. During their lifetime or at the time of initial need for therapy, many patients have various degrees of renal or liver insufficiency. In the USA, the package inserts include information about these populations, but often there is more information available in the literature or is known to the pharmaceutical manufacturers. Here, we sought all available information to compile directions for the use of PH medications. Package inserts of medications were accessed online. A literature review was performed on each medication with associated keywords search. Medical affairs departments of pharmaceutical manufacturers were also contacted regarding pharmacodynamics data at their disposition. It is possible to find information to prescribe PH medications in each pathway. In patients with renal conditions, ERAs require no adjustment, nor does sildenafil. All but the most severe patients may get riociguat at lower dose. Treprostinil requires no renal adjustment. For patients with liver dysfunction, macitentan requires no adjustment. Tadalafil can be started at half dose in patients with mild to moderate liver failure and uptitrated. Riociguat can be titrated carefully in patients with mild to moderate liver disease. Selexipag can also be initiated at 200 mg once daily and slowly titrated in moderate liver disease. Iloprost and various forms of treprostinil can be used with slow titration. As our patients get older and have more significant comorbidities, greater attention should be given to medication selection, titration and dose adjustment in renal and liver disease. There are still many gaps in the data available to clinicians regarding safe use of PH medications in special populations. We encourage drug manufacturers and researchers to pay greater attention to this area of need.

## A073 CT FEATURES AND RISK FACTORS OF PULMONARY CEMENT EMBOLISM IN PATIENTS WITH VERTEBRAL COMPRESSION FRACTURE

Xuebiao Sun, Xiapei Meng, Wenqing Xu, Mei Deng, Peiyao Zhang, Haishuang Sun, Min Liu

*Peking University, Beijing; China–Japan Friendship School of Clinical Medicine, Beijing; Chinese Academy of Medical Sciences & Peking Union Medical College, Beijing, China; Department of Radiology, China–Japan Friendship Hospital, Beijing, China; Department of Respiratory Medicine, The First Hospital of Jilin University, National Clinical Research Center for Respiratory Diseases, Changchun, China*

Our objective was to explore computed tomography (CT) features and risk factors for pulmonary cement embolism (PCE) in patients with vertebral compression fracture. Three hundred and seventy‐three patients (male 96, mean age 78.0 ± 9.4 years old) were retrospectively included. Their clinical data were recorded and postprocedural chest CT reviewed for evaluation of pulmonary cement emboli. Of all 373 patients, 258 patients underwent percutaneous vertebroplasty (PVP) and the other 115 underwent percutaneous kyphoplasty (PKP). Pulmonary cement embolism was found in 64 patients (17.2%) on postprocedural chest CT images. The typical CT findings of PCE were multiple lines or branching hyperintensity in pulmonary arteries. The upper lobe of bilateral lungs were mostly involved, followed by the middle lobe of the right lung. Meanwhile, CT indicated that 103 cases had cement emboli in the azygos vein and eight cases had cement emboli in the inferior vena cava. Binary logistic regression analysis demonstrated that PVP/PKP in T9 vertebra, cement emboli in the azygos vein and inferior vena cava were the risk factors for PCE [T9, odds ratio (OR) = 4.222, 95% confidence interval (CI), 1.490–11.966; cement emboli in azygos vein, OR = 7.647, 95% CI, 3.937–14.856; cement emboli in inferior vena cava, OR = 42.701, 95% CI, 7.525–242.302]. The incidence of PCE during PVP or PKP was 17.2%. Postprocedural chest CT can clearly show PCE as hyperintensive lines in pulmonary arteries. T9 vertebra, cement emboli in azygos vein and inferior vena cava were the risk factors of PCE.

## A074 PERIPHERAL BLOOD T CELLS OF PATIENTS WITH IDIOPATHIC PULMONARY ARTERIAL HYPERTENSION HAVE A REDUCED CYTOKINE‐PRODUCING CAPACITY

Thomas Koudstaal^*^, Denise van Uden^*^, Jennifer A C van Hulst, Madelief Vink, Menno van Nimwegen, Leon M van den Toorn, Prewesh P Chandoesing, Annemien E van den Bosch, Mirjam Kool, Rudi W Hendriks, Karin A Boomars

*Department of Pulmonary Medicine; Department of Cardiology, Erasmus MC, University Medical Center Rotterdam, Rotterdam, The Netherlands*

Pulmonary arterial hypertension (PAH) is rare disease that is categorized as idiopathic (IPAH) when no underlying cause can be identified. The finding that the lungs of most patients with IPAH contain increased numbers of T cells and dendritic cells (DCs) suggests the involvement of the immune system in its pathophysiology. To date, a detailed characterization of circulating immune cells in IPAH is lacking. We used flow cytometry to characterize peripheral blood DCs and T cells in treatment‐naïve IPAH patients, compared with connective tissue disease–PAH (CTD‐PAH) patients and healthy controls (HCs). At diagnosis, T‐helper (Th) cells of IPAH patients were less capable of producing tumor necrosis factor‐α, interferon‐γ, interleukin (IL)‐4 and IL‐17 compared with HCs. The T‐cell compartment of IPAH patients showed an increase of Th2 cells and enhanced expression of the CTLA4 checkpoint molecule in both naïve and memory CD4^+^ and CD8^+^ T cells. Expression of the PD‐1 checkpoint molecule or the activation marker ICOS was normal. In contrast, CTD‐PAH patients showed increased expression of both CTLA4 and ICOS. Frequencies and surface marker expression of circulating DCs and monocyte subsets were essentially comparable between IPAH patients and HCs. Principal components analysis separated IPAH patients, but not CTD‐PAH patients, from HCs, based on T‐cell cytokine profiles. At 1‐year follow‐up, the frequencies of IL‐17^+^ production by memory CD4^+^ T cells was increased in IPAH patients and was accompanied by an increase in the proportions of Th17 and Tc17 cells, as well as decreased CTLA4 expression. Principal components analysis separated IPAH patients, but not CTD‐PAH patients, at diagnosis from patients at 1‐year follow‐up. Treatment‐naïve IPAH patients displayed a unique T‐cell phenotype that was different from CTD‐PAH patients and was characterized by a reduced cytokine‐producing capacity. These findings point to involvement of adaptive immune responses in IPAH, which might have implications for development of therapeutic interventions.

## A075 CONTRIBUTIONS OF MYOCARDIAL HYPERTROPHY AND STIFFENING TO SEX DIFFERENCES IN RIGHT VENTRICULAR REMODELING IN A RAT MODEL OF PULMONARY ARTERIAL HYPERTENSION

Ethan D, Kwan, Hao Mu, Kristen Garcia, Daniela Valdez‐Jasso

*Department of Bioengineering, University of California, San Diego, La Jolla, CA, USA*

Right ventricular (RV) function is an important prognostic indicator in pulmonary arterial hypertension (PAH), a progressive vasculopathy that stimulates RV hypertrophy and chamber remodeling. Women are twice as likely to develop PAH but fair better than men^1^. Combining RV hemodynamic and morphologic measurements with a mathematical model of RV biomechanics, we investigated sex differences in systolic and diastolic chamber function in a rat model of PAH. Age‐matched male and female rats were treated with sugen‐hypoxia (SuHx) to induce PAH. Right ventricular pressure–volume (PV) time series were measured during the steady state and during changes in preload. Right ventricular pressure, myocardial wall mechanics and morphology were related using a RV biomechanics model^2^. Model analysis distinguished the relative contributions of chamber volume, geometry and myocardial material properties. In SuHx rats, RV end‐systolic pressures increased and RVs hypertrophied. End‐diastolic pressures rose three‐ to fourfold in the male SuHx group but remained unchanged in female SuHx rats. Despite differences in blood pressures, the ejection fraction was maintained in both groups. Predicted sarcomere length–tension relations showed passive stiffening only in male SuHx rats (*p* < 0.01). In contrast, active length–tension relations were remodeled only in the female SuHx group. In this study, diastolic chamber PV remodeling in male rats was explained largely by myocardial passive stiffening, matching previous clinical reports^3^. Diastolic stiffness in this study was not associated with extracellular matrix collagen accumulation but could be explained by fibrillar collagen structural changes. Model predictions of myocardial material remodeling are yet to be validated, with planned *ex vivo* experiments including biaxial testing of myocardium (passive) and calcium‐dependent contractility measurements from isolated cardiomyocytes (active). This study demonstrates distinct sex‐dependent phenotypes of RV remodeling, emphasizes the importance of investigating ventricular adaptation to PAH in both males and females, and proposes mechanisms that would explain sex‐dependent outcomes.

## A076 ALTERED CALCIUM–CYTOSKELETAL DYNAMICS IMPAIR ENDOTHELIAL WOUND RESPONSES IN CHRONIC THROMBOEMBOLIC PULMONARY HYPERTENSION

Corey Wittig, Xue D Manz, Thang V Pham, Connie R Jimenez, Petr Symersky, Harm‐Jan Bogaard, Robert Szulcek

*Laboratory of In Vitro Modeling Systems of Pulmonary and Thrombotic Diseases, Institute of Physiology, Charité – Universitätsmedizin Berlin, corporate member of Freie Universität Berlin and Humboldt‐Universität zu Berlin, Berlin, Germany; Department of Pulmonary Diseases, Amsterdam UMC, VU University Medical Center, Amsterdam Cardiovascular Sciences (ACS), Amsterdam; OncoProteomics Laboratory, Cancer Center Amsterdam, Amsterdam UMC, VU University Medical Center, Amsterdam; Department of Cardio‐thoracic Surgery, Amsterdam UMC, VU University Medical Center, Amsterdam; Department of Pulmonary Diseases, Amsterdam UMC, VU University Medical Center, Amsterdam Cardiovascular Sciences (ACS), Amsterdam, The Netherlands; German Heart Center Berlin, Berlin, Germany*

Chronic thromboembolic pulmonary hypertension (CTEPH) is clinically defined by a pathological increase in mean pulmonary artery pressure caused by thrombotic obstruction of the pulmonary vasculature, persisting after effective anticoagulation therapy. Proposed causes for CTEPH include pulmonary arteriopathy triggering in situ thrombosis. We hypothesize that pulmonary artery endothelial cells (PAECs) of CTEPH patients show impaired wound healing capabilities, leading to prolonged exposure of subendothelial proteins to circulating hemostatic factors and increasing the propensity for pulmonary in situ thromboses. Pulmonary artery endothelial cells were isolated from human pulmonary endarterectomy samples of CTEPH patients or donor lung lobectomy tissues. The study was approved by the institutional review board of the VU University Medical Center (2019.231), and written informed consent was given. The isolated cells were expanded in culture, submitted to global proteomic analysis, subjected to electrical and mechanical wound healing assays, and intracellular calcium (Ca^2+^) was quantified. The CTEPH proteome demonstrates significantly altered levels of proteins associated with the cytoskeleton, including CAPZA2, ACTG1 and ARPC5. Additionally, CTEPH‐PAECs highly express the calcium‐/calmodulin‐binding proteins NUCB1, ANXA4 and FBL. Wound healing assays reveal that CTEPH‐PAECs exhibit an initial migratory lag phase after wounding, where the time required to close 25% of the wound almost doubles compared with controls (*p* < 0.001). Subsequently, while keeping proliferation rates constant, CTEPH‐PAECs gradually increase migration rate up to 2.5‐fold their initial rate (*p* < 0.001). Analysis of intracellular Ca^2+^ levels revealed a biphasic intracellular Ca^2+^ decay with a plateau phase after histamine stimulation in CTEPH‐PAECs but not controls. Our data show delayed early‐phase migration after wounding in CTEPH‐PAECs, potentially attributable to altered intracellular Ca^2+^ dynamics and the associated cytoskeletal reorganization response. We postulate that the prolonged exposure of subendothelial proteins after wounding favors activation of platelets and their binding, promoting prothrombotic conditions in the pulmonary vasculature of patients with CTEPH.

## A077 BEST PRACTICES FOR UNSUPERVISED MACHINE LEARNING TO STRATIFY PATIENTS FROM HIGH‐DIMENSIONAL MOLECULAR PROFILES

Sokratis Kariotis, Emmanuel Jammeh, Allan Lawrie, Dennis Wang

*Department of Neuroscience, University of Sheffield, Sheffield, UK*

A variety of machine learning approaches are currently being used to develop new diagnostics and discover new disease subtypes (endophenotypes) across medical specialties, including pulmonary hypertension. Unsupervised machine learning approaches are required to address uncertainty in complex diseases where the exact diagnosis is undetermined and/or the amount of data from patients is too large for direct interpretation. Large datasets, such as electronic patient records or ‐omics, have highlighted the importance of expertly selected unsupervised clustering methodologies. There is currently a lack of systematic ways in which to tune data for the unbiased estimation of patient subgroups from cohorts and validate new patient clusters through mathematical metrics. To address this, we have created a toolkit with best practices for non‐machine learning experts to test a variety of clustering methods, select relevant biomarkers and decide on the optimal number of patient subgroups based on stability metrics and clinical evidence. The tools can be implemented in an automated pipeline that can help to discover subtypes from any type of high‐dimensional molecular data (‐omics) from patient tissue. We have previously demonstrated its utility on blood RNA profiles from pulmonary hypertension patients, where we identified biologically relevant subgroups with significantly different clinical outcomes and unique gene signatures and are currently validating in other ‐omic datasets.

## A078 LONG‐TERM SURVIVAL OF PATIENTS WITH PULMONARY ARTERIAL HYPERTENSION IN A JAPANESE REFERRAL CENTER

Ayane Miyagi, Hiroto Shimokawahara, Yoichi Sugiyama, Takeshi Suetomi, Aiko Ogawa, Hiromi Matsubara

*Department of Cardiology, National Hospital Organization Okayama Medical Center, Okayama, Japan*

Outcomes at >10 years in patients with pulmonary arterial hypertension (PAH) remain unknown. This study was aimed to investigate the long‐term prognosis and to clarify factors that determine the survival at a single referral center in Japan. We retrospectively reviewed 186 consecutive PAH patients in our hospital from May 1999 to September 2021. Long‐term survival rates and determinants were investigated. Eighty‐two patients were idiopathic/heritable, 57 patients were associated with connective tissue disease, 21 patients were associated with congenital heart disease, 24 patients were porto‐pulmonary hypertension (PoPH), and two patients were drug‐induced PAH. Median time from diagnosis to the latest follow‐up was 6.0 years [interquartile range (IQR), 2.7–11.0]. Fifty‐two patients died, and four patients underwent lung transplantation during the follow‐up period. The 5‐, 10‐, 15‐ and 20‐year cumulative survival was 76.7, 61.7, 56.4% and 49.4%, respectively. At baseline, prognostic factors for survival were non‐PoPH (*p* < 0.001) and low‐risk status of ESC/ERS risk stratification (*p* = 0.015). And at the latest follow‐up, prognostic factors for survival were low‐risk status of ESC/ERS risk stratification (*p* < 0.001), percentage predicted diffusing capacity of lung (*p* = 0.001) and mean pulmonary arterial pressure (mPAP) (*p* = 0.028). Median mPAP decreased from 50.0 mmHg [IQR, 39.0–61.0] to 28.0 mmHg [IQR, 21.0–39.0] after initiation of PAH‐specific therapy (*p* < 0.05). The 20‐year cumulative survival rate in patients with mPAP at the latest follow‐up of <21, 22–28, 29–39 and >40 mmHg was 85.9, 65.4, 64.5% and 27.0%, respectively. The lower the mPAP at the follow‐up, the better the prognosis of the patient.

Survival at >10 years in PAH patients was still unsatisfactorily. To obtain the best long‐term survival in PAH patients, aggressive therapy aiming at normalization of mPAP seems to be necessary.

## A079 TARGETING METABOLIC SYNDROME REVERSAL THROUGH NUTRITIONAL KETOSIS IMPROVES QUALITY OF LIFE AND EXERCISE TOLERANCE IN PULMONARY HYPERTENSION ASSOCIATED WITH HEART FAILURE WITH PRESERVED EJECTION FRACTION (PH‐HFPEF): EARLY FINDINGS FROM THE REVERSE‐HFPEF STUDY

Darlene Kim, Dthia Kalkwarf, Mohammad Dalabih, Cole Waliczek, Maria Saucedo Garcia, Zulma Yunt, Vera Pilliteri, M Patricia George

*Department of Medicine, National Jewish Health, Denver, CO, USA*

Pulmonary hypertension (PH) is a major driver of morbidity and mortality when it complicates heart failure with preserved ejection fraction (HFpEF). Of all group 2 PH sub‐phenotypes, PH‐HFpEF is the most common. An increasing body of evidence supports metabolic syndrome (MetS) as a key contributor to the development of PH‐HFpEF. Nutritional ketosis successfully reverses Type 2 diabetes and MetS^1^. We hypothesized that a medically supervised ketogenic diet (MSKD) in patients with MetS and PH‐HFpEF would improve quality of life (QOL) and exercise tolerance. We report an interim analysis of our novel pilot study. We prospectively enrolled subjects with MetS and PH‐HFpEF diagnosed by right heart catheterization (mean pulmonary artery pressure > 25 mmHg, PAOP > 15 mmHg), with a cardiopulmonary exercise test (CPET) and echocardiogram within 6 months before enrollment and with stable weight and medication regimens. Subjects were enrolled in MSKD education for 1 month. After baseline laboratory, imaging, exercise and QOL assessments, subjects initiated a 6‐month MSKD intervention with regular safety checks including laboratory tests, physical examinations and daily symptom surveys. Subjects measured serum β‐hydroxybutyrate ketone and glucose levels daily. Complete re‐assessments were repeated at 3 and 6 months of the intervention. After 6 months, there was a decrease in mean weight, body mass index and waist circumference. The Minnesota Living with Heart Failure Questionnaire (MLHFQ) score decreased on average by 24 points, reflecting substantially improved QOL. On CPET, the average peak work achieved increased by 7 W, and the average ratio of work to peak oxygen consumption achieved increased by 16.8 W/L/min. Additionally, all other exercise measures improved. A, MSKD is safe in patients with PH‐HFpEF and MetS and substantially improves QOL. Improvement in validated QOL scores might be driven by the consistent improvement in exercise tolerance seen in our study.

## A080 MITOQ TREATMENT AS THERAPEUTIC APPROACH FOR MALADAPTIVE RIGHT HEART REMODELING

Oleg Pak, Claudia F Garcia Castro, Stefan Hadzic, Akylbek Sydykov, Marija Gredic, Matthias Hecker, Michael P Murphy, Hossein A Ghofrani, Ralph T Schermuly, Werner Seeger, Norbert Weissmann, Natascha Sommer

*Excellence Cluster Cardiopulmonary System, University of Giessen and Marburg Lung Center (UGMLC), member of the German Center for Lung Research (DZL), Justus‐Liebig‐University, 35392 Giessen, Germany; MRC Mitochondrial Biology Unit, Cambridge, UK; Max Planck Institute for Heart and Lung Research, 61231 Bad Nauheim, Germany*

Oxidative stress may promote maladaptive right ventricular (RV) remodelling. We previously showed that treatment with the mitochondria‐targeted antioxidant MitoQ attenuated murine chronic hypoxia‐ and pressure overload‐induced RV remodelling. We thus now investigated the effect of MitoQ treatment on tobacco smoke‐induced RV remodelling and pulmonary hypertension (PH). Mice were exposed to cigarette smoke for 8 months (6 h per day, 5 days per week) and subsequently treated with 500 µM MitoQ (Antipodean Pharmaceuticals, Auckland, New Zealand) or its inactive carrier substance, decyltriphenylphosphonium (TPP^+^), for 3 months applied via the drinking water. Three months after the cessation of smoke exposure, TPP^+^‐treated animals (TPP^+^‐SE) exhibited increased RV systolic pressure (RVSP), RV remodelling and dysfunction, in addition to signs of cigarette smoke‐induced emphysema compared with mice that were exposed to room air for the whole experimental time period of 11 months (TPP^+^‐RA). MitoQ‐treated and smoke‐exposed animals (MitoQ‐SE) showed significantly lower RVSP 3 months after re‐exposure to room air compared with TPP^+^‐SE animals (RVSP: MitoQ‐SE 22.5 ± 0.6 mmHg vs. TPP^+^‐SE 27.3 ± 0.5 mmHg, *p* < 0.0001), and similar RVSP to TPP^+^‐RA mice (21.6 ± 0.5 mmHg, *p* < 0.0001 compared with TPP^+^‐SE). Furthermore, MitoQ‐treated mice showed less dilatation of the RV (RV inner diameter: MitoQ‐SE 0.97 ± 0.03 mm vs. TPP^+^‐SE 1.16 ± 0.03 mm, *p* = 0.0001), but no statistically significant difference of RV wall thickness compared with TPP^+^‐SE mice. Right ventricular function, determined by tricuspid annular plane systolic excursion (TAPSE), was also improved compared with TPP^+^‐SE (TAPSE: MitoQ‐SE 1.28 ± 0.01 mm vs. TPP^+^‐SE 1.18 ± 0.02 mm, *p* = 0.0035) and restored to the level of control TPP^+^‐RA mice (1.26 ± 0.01 mm, *p* < 0.0001 compared with TPP^+^‐SE). Moreover, MitoQ treatment improved lung function (static compliance: MitoQ‐SE 0.09 ± 0.002 mL/cmH_2_O vs. TPP^+^‐SE 0.11 ± 0.004 mL/cmH_2_O, *p* = 0.0076) in mice with fully established cigarette smoke‐induced emphysema. In conclusion, MitoQ could be a new potential treatment for cigarette smoke‐induced PH and for RV insufficiency of different aetiologies.

## A081 PULMONARY VASCULAR PATHOLOGY DUE TO HIV AND SCHISTOSOMA COEXPOSURE IS ASSOCIATED WITH AN ALTERED IMMUNE RESPONSE

Sandra Medrano‐Garcia, Daniel Morales‐Cano, Bianca Barreira, Rahul Kumar, Ralph Theo Schermuly, Brian B Graham, Djuro Kosanovic, Francisco Perez‐Vizcaino, Alistair Mathie, Rajkumar Savai, Soni Pullamseti, Ghazwan Butrous, Angel Cogolludo, Edgar Fernández‐Malavé

*Department of Immunology, Ophthalmology and ENT, Complutense University School of Medicine and 12 de Octubre Health Research Institute (imas12), Madrid; Department of Pharmacology and Toxicology, School of Medicine, Universidad Complutense de Madrid, Instituto de Investigación Sanitaria Gregorio Marañón, Centro de Investigación Biomédica en Red Enfermedades Respiratorias, Madrid; Centro Nacional de Investigaciones Cardiovasculares (CNIC), Madrid, Spain; Department of Medicine, University of California, San Francisco, CA, USA; Sechenov First Moscow State Medical University (Sechenov University), Moscow, Russia; Department of Internal Medicine, Justus‐Liebig University, Member of the German Center for Lung Research (DZL), Giessen, Germany; Medway School of Pharmacy, University of Kent and University of Greenwich, Chatham, UK; Max Planck Institute for Heart and Lung Research, Member of the German Center for Lung Research (DZL), Member of the Cardio‐Pulmonary Institute (CPI), Bad Nauheim; Institute for Lung Health (ILH), Justus Liebig University, Giessen, Germany*

HIV and *Schistosoma* infections have been associated individually with pulmonary vascular disease. By using a noninfectious animal model based on lung embolization of *Schistosoma mansoni* eggs in HIV‐1 transgenic (HIV) mice, we have previously shown that HIV and *Schistosoma* co‐exposure results in augmented pulmonary arterial pressure associated with enhanced vascular remodeling and dysfunction. Given that immune responses are a major driver of tissue damage in various pulmonary diseases, we analyzed the pulmonary immune landscape of co‐exposed mice. HIV mice displayed an impaired immune response to parasite eggs in the lung, as suggested by decreased pulmonary leukocyte infiltration, with altered myeloid‐to‐lymphoid ratio. Analysis of the pulmonary immune landscape of co‐exposed mice revealed increased abundance of γδ T cells with upregulated TCR, patrolling‐type monocytes and interstitial macrophages; and heightened expression of pro‐inflammatory/profibrotic cytokines, including inerferon‐γ and interleukin‐17, by both CD4^+^ and γδ T cells, and interleukin‐13 in myeloid but not T cells. Taken together, these results suggest that alterations in the immune landscape could underlie, in part, the structural and functional changes in the pulmonary vasculature observed in mice co‐exposed to HIV and *Schistosoma* and, ultimately, contribute to the development of vascular pathology.

## A082 SMYD2‐MEDIATED HIF‐1A STABILIZATION PLAYS A PROTECTIVE ROLE IN RIGHT VENTRICULAR HYPERTROPHY

Swathi Veeroju, Baktybek Kojonazarov, Argen Mamazhakypov, Aysel Ashir, Nabham Rai, Sandra Breuils‐Bonnet, Jochen Wilhelm, Norbert Weissmann, Steve Provencher, Sebastien Bonnet, Soni Savai Pullamsetti, Werner Seeger, Ralph T Schermuly, Tatyana Novoyatleva

*Universities of Giessen and Marburg Lung Center (UGMLC), Excellence Cluster Cardio‐Pulmonary Institute (CPI), Member of the German Center for Lung Research (DZL), Justus‐Liebig University, Giessen, Germany; Institute for Lung Health, Giessen, Germany; Max Planck Institute for Heart and Lung Research, Bad Nauheim, Germany, Universities of Giessen and Marburg Lung Center (UGMLC), Excellence Cluster Cardio‐Pulmonary Institute (CPI), Member of the German Center for Lung Research (DZL), Justus‐Liebig University, Giessen, Germany; Pulmonary Hypertension and Vascular Biology Research Group, Institut Universitaire de Cardiologie et de Pneumologie de Québec, Université Laval, Department of Medicine, Québec, Canada; Universities of Giessen and Marburg Lung Center (UGMLC), Excellence Cluster Cardio‐Pulmonary Institute (CPI), Member of the German Center for Lung Research (DZL), Justus‐Liebig University, Giessen, Germany*

Pulmonary hypertension upon disease progression leads to heart failure. Previous observations encompass that angiogenesis was severely dysregulated in the failing heart and that targeting proangiogenic factors, such as HIF‐1A, might play a crucial role in maintenance of right ventricular (RV) function. Smyd2 is a protein methyltransferase known to be highly expressed in the heart, and *in silico* analysis demonstrated the presence of the highly conserved Smyd2 substrate motif in HIF‐1A. In this study, we hypothesize that Smyd2‐mediated methylation might play a crucial role in HIF‐1A‐mediated angiogenesis and RV hypertrophy. Expression analysis from human RVs demonstrated that Smyd2 and HIF‐1A were upregulated in compensated RVs and downregulated in decompensated RVs. A similar expression pattern was observed in RVs from pulmonary arterial banded (PAB) mice. Interestingly, microarray analysis from Smyd2 overexpression in neonatal rat ventricular cardiomyocytes (NRCMs) showed significant upregulation of proangiogenic genes. Mass spectrometry analysis revealed HIF‐1A as a novel substrate of Smyd2 methyltransferase activity; this was further substantiated by utilization of a specific HIF‐1A methylation‐specific antibody and overexpression of a HIF‐1A methylation site‐deficient mutant. Both NRCMs and human cardiac microvascular endothelial cells (HMEC‐C) exposed to hypoxia showed Smyd2 expression, and interactions between HIF‐1A and Smyd2 were studied by immunoprecipitation. Nuclear and cytoplasmic extracts and immunofluorescence staining in NRCMs exposed to hypoxia revealed that Smyd2 overexpression leads to HIF‐1A stability and HIF‐1A nuclear translocation, whereas this expression was diminished by Smyd2 Y240F overexpression. Interestingly, knockdown of Smyd2 reduces hypoxia‐induced HIF‐1A protein stability in NRCMs and HMEC‐C. Cardiomyocyte‐specific overexpression of Smyd2 using an adeno‐associated viral approach (AAV9‐CMVIVS‐mSmyd2) in PAB mice displayed improved cardiac function associated with HIF‐1A stabilization, activation of proangiogenic factors and significant reduction in RV fibrosis. Our study demonstrates that HIF‐1A is a novel substrate of Smyd2 methylation and AAV mediated Smyd2 overexpression displayed cardioprotective effects.

## A083 PROFOUND LOSS OF LUNG C‐KIT‐EXPRESSING ENDOTHELIAL CELLS DURING THE EARLY DEVELOPMENT OF SEVERE PULMONARY ARTERIAL HYPERTENSION POINTS TO AN IMBALANCE BETWEEN INJURY AND REPAIR IN INITIATION OF LUNG VASCULAR DISEASE

Nicholas D Cober, Emma McCourt, Rafael Soares Godoy, Yupu Deng, Ken Schlosser, David P Cook, Liyuan Wang, Duncan J Stewart

*Ottawa Hospital Research Institute, University of Ottawa, Ottawa, ON, Canada*

Pulmonary arterial hypertension (PAH) is a devastating disease characterized by occlusive vascular remodeling and increased pulmonary vascular resistance and triggered by endothelial cell (EC) injury and apoptosis. We hypothesized that an imbalance in lung EC injury and repair plays a central role in arterial remodeling in PAH. We used single‐cell RNA sequencing (scRNA‐seq) to study transcriptomic changes during PAH progression. Sprague–Dawley rats were injected with 20 mg/kg SU5416 (SU) and exposed to 3 weeks of chronic hypoxia (CH, 10% O_2_), followed by a return to normoxia for 5 weeks. Right ventricular systolic pressure (RVSP) was measured at baseline, 1, 3, 5 and 8 weeks. At each time point, lungs were digested and dissociated into single cells, which were sequenced (10× genomics). Elevation of RVSP was observed at 1 week after SU (43 ± 4 mmHg), with progression to 102 ± 11 mmHg at 5 weeks, persisting until 8 weeks. Dimensionality reduction of the scRNAseq data was performed using uniform manifold approximation and projection (UMAP) analysis, resulting in 17 lung clusters. Subclustering of EC populations produced seven distinct clusters, including all expected subtypes (capillary, arterial, venous and lymphatic), in addition to a novel ‘activated’ population that emerged after 1 week and persisted throughout PAH development. This cluster was characterized by downregulation of typical endothelial genes (*Cldn5* and *Cdh5*) and upregulation of genes involved in antigen presentation (*Cd74* and *RT1‐Da*), suggesting a role in vascular inflammation. Moreover, *cKit* expression, which was largely restricted to general capillary (gCap) ECs, was almost completely abolished at 1 week after SU and throughout PAH progression. The early expansion of activated, pro‐inflammatory ECs, together with depletion of regenerative *cKit* ^+^ ECs, represents an imbalance in mechanisms of lung microvascular injury and repair in the initiation and progression of PAH in the SU/CH model.

## A084 PARADOXICAL APELIN EXPRESSION BY GCAP ALVEOLAR ENDOTHELIAL STEM CELLS: ROLE IN MICROVASCULAR REGENERATION AFTER ACUTE LUNG INJURY INDUCED BY TARGETED ENDOTHELIAL CELL ABLATION

Rafael Soares Godoy, David P Cook, Nicholas D Cober, Emma McCourt, Yupu Deng, Liyuan Wang, Ken Schlosser, Katelynn Rowe, Duncan J Stewart

*Ottawa Hospital Research Institute, University of Ottawa, Canada*

Endothelial cell (EC) injury is thought to contribute to breakdown of the air–blood barrier in acute lung injury (ALI), and EC regeneration is necessary for ALI resolution. Therefore, we sought to define the cellular and molecular mechanisms underlying lung microvascular regeneration in a severe ALI model induced by selective lung EC ablation. Changes in lung cell populations and gene expression profiles were determined in transgenic mice expressing human diphtheria toxin (DT) receptor targeted to ECs. Lungs were isolated, digested and subjected to single‐cell RNA sequencing at baseline (day 0) and days 3, 5 and 7 after EC ablation induced by intratracheal DT instillation. Eight distinct endothelial clusters were resolved, including alveolar aerocyte (aCap) ECs expressing apelin at baseline and general capillary (gCap) ECs expressing the apelin receptor. Intratracheal instillation of DT resulted in ablation of >70% of lung ECs, producing severe ALI with near complete resolution by 7 days. At 3 days post‐injury, a novel gCap population emerged, characterized by *de novo* expression of apelin together with the stem cell marker, protein C receptor. These stem‐like cells transitioned to proliferative ECs, expressing apelin receptor together with the pro‐proliferative transcription factor, FoxM1. This progenitor‐ like cell population was responsible for the rapid replenishment of all depleted EC populations by 7 days post‐injury, including aerocytes, which play a crucial role in re‐establishment of the air–blood barrier. Treatment with an apelin receptor antagonist prevented recovery and resulted in excessive mortality, consistent with a central role for apelin signaling in EC regeneration and microvascular repair. The lung has a remarkable capacity for microvascular EC regeneration, which is orchestrated by newly emergent apelin‐expressing gCap endothelial stem‐like cells that give rise to highly proliferative, apelin receptor‐positive endothelial progenitors.

## A085 DISCREPANT PROGNOSTIC PREDICTION OF RIGHT VENTRICLE PULMONARY ARTERY COUPLING IN GROUP 2 PULMONARY HYPERTENSION DUE TO HEART FAILURE WITH PRESERVED EJECTION FRACTION COMPARED WITH HEART FAILURE WITH REDUCED EJECTION FRACTION

Yochai Adir, Saleh Nazzal, Shemy Carasso, Claudia Simsolo, Marwan Amara, Marco Guazzi

*Pulmonary Division, Lady Davis Carmel Medical Center, Faculty of Medicine Technion Institute of Technology, Haifa; Pulmonary Division, Baruch Padeh Medical Center, Faculty of Medicine, Azrieli Faculty of Medicine, Bar Ilan University, Safed; Cardiology Division, Shaare Zedek Medical Center, Jerusalem, Israel; University of Milano, University Cardiology Department, San Paolo Hospital, Milano, Italy*

Pulmonary hypertension (PH)‐induced right ventricular (RV) failure indicates a poor prognosis in heart failure (HF). Right ventricular–pulmonary artery coupling (RVPAC) may refine this association, clarifying group 2 PH even at early stages, its hemodynamic correlate of impairment and outcome prediction. The aim was to assess the association between RVPAC and mortality in patients with group 2 PH owing to HF with preserved ejection fraction (HFpEF) and HF with reduced ejection fraction (HFrEF). Heart failure patients with systolic pulmonary artery pressure (PASP) > 40 mmHg on echocardiography were included in the study. The RVPAC ratio was calculated by dividing the tricuspid annular plane systolic excursion (TAPSE) by the PA systolic pressure (PASP) from transthoracic echocardiograms. Cox proportional hazard regression models were used to assess the association between RVPAC and mortality and Kaplan–Meier with the primary outcome of time to death. Six hundred and nine patients were included in the study. The average age was 71 ± 13.2 years, and 301 were female. Two hundred and eight patients had HFrEF, and 401 had HFpEF. Low RVPAC was found to increase the risk of mortality significantly (*p* < 0.038). However, when we evaluate the mortality risk prediction in the HFpEF group as opposed to the HFrEF group, RVPAC was found to be an independent predictor of mortality only in patients with HFpEF (*p* < 0.001). In group 2 PH, RVPAC is an independent risk factor for mortality, with a discrepant prognostic value between HFpEF and HFrEF. This discrepancy might be explained by an intrinsic pathology of the RV in patients with advanced HFrEF that affects pressure increase, yielding low TAPSE and low PASP and resulting in a pseudo‐normal TAPSE/PASP ratio.

## A086 EXOSOMES INDUCE DNA DAMAGE RESPONSE IN CARDIOMYOCYTES WITH CARDIAC REMODELING AND DYSFUNCTION IN MICE WITH PERINATAL OBESITY

Jaco Selle, Dennis Mehrkens, Deniz Bartsch, Hendrik Hennig, Simon Geißen, Baktybek Kojonazarov, Xiaojie Yu, Margarete Odenthal, Christina Vohlen, Rebecca Wilke, Simon Grimm, Uta Eule, Martin Mollenhauer, Stephan Baldus, Soni S Pullamsetti, Leo Kurian, Jörg Dötsch, Miguel A Alejandre Alcazar

*Translational Experimental Pediatrics – Experimental Pulmonology, Department of Pediatric and Adolescent Medicine; Center for Molecular Medicine Cologne (CMMC); Institute for Neurophysiology; Cologne Excellence Cluster on Stress Responses in Aging‐associated Diseases (CECAD); Institute of Pathology; Center of Integrated Oncology Cologne‐Bonn; Department III of Internal Medicine, Heart Center, Faculty of Medicine and University Hospital Cologne, Cologne, Germany; Justus‐Liebig University Gießen, German Centre for Lung Research (DZL), Institute for Lung Health (ILH), University of Giessen and Marburg Lung Center (UGMLC), Gießen; Max‐Planck‐Institute for Heart and Lung Research, Department of Lung Development and Remodeling, Member of the German Center for Lung Research (DZL), Bad Nauheim, Germany*

Obesity and metabolic disorders are intimately linked to cardiopulmonary diseases. Accumulating clinical and experimental evidence suggests that maternal obesity determines child health. White adipose tissue (WAT) is an active endocrine organ with subacute chronic inflammatory effects on the cardiopulmonary system. Studies from our group demonstrated that perinatal obesity induces bronchial and vascular remodeling through interleukin‐6, resulting in bronchial obstruction and pulmonary hypertension. Given that obesity and aging processes converge in similar pathways, we tested whether perinatal obesity adversely affects cardiac remodeling by early activation of the DNA‐damage response (DDR), a hallmark of aging. (1) To study the impact of perinatal obesity on the cardiopulmonary system. (2) To test whether serum exosomes derived from obese dams or offspring mediate cardiac remodeling.

Female mice were fed with high‐fat diet (HFD) or standard‐diet (SD) before mating and during pregnancy and lactation. The hearts of offspring were analyzed at postnatal day 21 (P21) and 1.5 years. Inducible pluripotent stem cell (iPSC)‐derived cardiomyocytes were exposed to exosomes from dams or their respective P21 offspring. (1) Quantitative histomorphometry revealed right ventricular remodeling, with hypertrophy of cardiomyocytes and increased collagen deposition, in hearts of HFD offspring. These structural changes were related to increased γH2AX^+^, indicating early onset of DDR after perinatal obesity. This early cardiac remodeling was linked to functional impairment at P70 that was evident even after 1.5 years. (2) Exposure of iPSC‐derived cardiomyocytes to serum exosomes of HFD offspring induced DDR and apoptosis, characteristics of aging. Subsequent proteomics and RNASeq transcriptomic profiling followed by KEGG‐pathway analysis identified pathways involved in survival, DDR and DNA integrity. Our data demonstrate that perinatal obesity induces cardiac remodeling and genomic instability, indicating early activation of aging processes in cardiomyocytes. We identified a perinatal WAT–heart axis through exosomes that might cause premature cardiac aging and determine susceptibility to cardiac diseases.

## A087 NUDT21 LINKS ALTERNATIVE POLYADENYLATION WITH VASCULAR REMODELING IN PULMONARY ARTERIAL HYPERTENSION

Scott Collum, Rajarajan T Amirthalingam, Eric E Wagner, W Jim Zheng, James D West, Tingting W Mills, Harry Karmouty‐Quintana

*Department of Biochemistry and Molecular Biology*; Department of Internal Medicine (Division of Critical Care, Sleep and Pulmonary Medicine), *McGovern Medical School; School of Biomedical Informatics, The University of Texas Health Science Center at Houston, Houston, TX; Houston Methodist J. C. Walter Jr Transplant Center, Houston Methodist Hospital, Houston, TX; Department of Biochemistry and Biophysics, University of Rochester Medical Center, Rochester, NY; Department of Medicine, Division of Allergy, Pulmonary and Critical Care Medicine, Vanderbilt University Medical Center, Nashville, TN, USA*

Pulmonary arterial hypertension (PAH) is characterized by a mean pulmonary arterial pressure (mPAP) >20 mmHg. A central pathophysiological process is vascular remodeling that results from enhanced proliferation of the endothelial and smooth muscle layers of the vessel, resulting in narrowing of the lumen. Our laboratory has recently discovered a novel molecular process known as alternative polyadenylation (APA) that is linked to increased cellular proliferation. A hallmark of APA is 3′ UTR shortening that leads to loss of binding sites for mRNA regulatory elements, allowing transcripts to escape mRNA regulation and resulting in heightened translation of affected transcripts. We have recently found that a protein that regulates APA, known as nudix hydrolase 21 (NUDT21), is depleted in the pulmonary arteries (PAs) of patients with PAH. We hypothesize that loss of NUDT21 contributes to vascular remodeling in PAH. Our data demonstrate reduced NUDT21 levels in isolated PAs from patients with a diagnosis of PAH. Using an experimental model of hypoxia–sugen (HX‐SU)‐induced PAH, we reveal reduced signals for NUDT21 concomitant with elevated right ventricular systolic pressure (RVSP). To demonstrate a causative role for loss of NUDT21 in PAH, mice lacking smooth muscle NUDT21 were exposed to HX‐SU, resulting in amplified RVSP. RNA‐seq analysis and subsequent determination of 3′ UTR lengths using bio‐informatic algorithms revealed RUNX1 as a downstream target for NUDT21 in pulmonary artery smooth muscle cells (PASMCs) lacking NUDT21. Using isolated normal human PASMCs, we demonstrate that knockdown of NUDT21 leads to elevated RUNX1 expression and enhanced cell proliferation that is blocked by Ro5‐3335, a RUNX1 inhibitor. Intriguingly, overexpression of NUDT21 in PAH‐derived PASMCs leads to reduced cell proliferation. We also report increased RUNX1 levels in experimental models and in isolated PAs in PAH patients. In conclusion, depletion of NUDT21 results in increased RUNX1 levels that promote cell proliferation in PAH.

## A088 CLASSICAL DENDRITIC CELLS DRIVE AND MAINTAIN HYPOXIA‐INDUCED PULMONARY HYPERTENSION

Claudia Mickael, Linda A Sanders, Michael H Lee, Rahul Kumar, Amy McKee, David Irwin, Delaney Swindle, Kurt Stenmark, Brian Graham, Rubin Tuder

*Division of Pulmonary Sciences and Critical Care Medicine, Division of Allergy and Clinical Immunology, Department of Medicine; Department of Pediatrics‐Critical Care Medicine, University of Colorado, Aurora, CO; Cardiovascular Pulmonary Research Laboratory, University of Colorado School of Medicine, Denver, CO; Division of Pulmonary Sciences, Department of Medicine, University of California, San Francisco, San Francisco, CA, USA*

Pulmonary hypertension (PH) is characterized by elevated right ventricular pressures and pulmonary vascular remodeling, resulting in heart failure and death. Exacerbated perivascular inflammation contributes to the pathophysiology of this disease. Classical dendritic cells (cDCs) secrete cytokines, chemokines and growth factors, which can result in recruitment of other inflammatory cells into their microenvironment. Our goal is to determine the role cDCs in hypoxia‐induced perivascular inflammation and PH. *Zbtb46* (zinc finger and BTB domain containing 46) is expressed exclusively by cDCs. We generated ZBTB46 DTR chimera mice with transplanted bone‐marrow cells originated from ZBTB46 DTR into wild‐type mice, which deplete cDCs upon diphtheria toxin (DT) injection. Animals treated with either vehicle or DT were hypoxia challenged (fraction of inspired O_2_ 10%), and right ventricular systolic pressure (RVSP) was assessed. We used ZBTB46^GFP^ all flow cytometry studies. We also performed scRNA‐Seq in cDCs of lungs of ZBTB46^GFP^ mice. We observed that the number of a subset of cDCs was increased in the lungs after acute exposure to hypoxia, indicating that these cells are involved in inflammation. When cDCs were depleted before hypoxia challenge, the mice were protected from PH, demonstrating that bone marrow‐derived cDCs (BMcDCs) are determinant in driving PH. When we depleted DCs after PH was established (14 days of hypoxia), and re‐exposed the mice (for 7 days), we observed PH protection, demonstrating that BMcDCs are determinant in reversing established PH. We observed fewer interstitial macrophages (IMs) after cDC depletion, suggesting that IM recruitment into the lungs is dependent on BMcDCs. Using SC‐RNA‐Seq, we observed that genes responsive to hypoxia were located in a particular cluster characterized by chemokine signaling, complement activation and expression of metalloproteinases. In conclusion, classical dendritic cells are important to drive and maintain hypoxia‐induced PH. Our studies open new avenues to gain a better understanding of the role of cDCs in hypoxia‐induced PH pathophysiology.

## A089 COMPARISON BETWEEN PULMONARY ARTERIAL HYPERTENSION RISK ASSESSMENT METHODS INCLUDING PHORA

Charles Fauvel, Zilu Liu, Shili Lin, Priscilla Correa‐Jaque, Amy Webb, Rebecca Vanderpool, Puneet Mathur, Manreet Kanwar, Jidapa Kraisangka, Adam Perer, Allen D Everett, Raymond Benza

*Internal Medicine Department, Division of Cardiovascular Medicine, Wexner Medical Center, The Ohio State University, Columbus, OH; Department of Statistics, Department of Biomedical Informatics, Research Information Technology, The Ohio State University, Columbus, OH; Temple University School of Medicine, Philadelphia, PA, USA; Mahidol University, Thailand; Carnegie Mellon University, Pittsburgh, PA; The John Hopkins University, Baltimore, MD, USA*

Risk assessment is crucial to predict survival and then guide therapeutic management for pulmonary arterial hypertension (PAH) patients. Several risk assessment methods have been developed, but the comparison of their performances using the same data set of PAH patients remains to be investigated. In the same patient's data set, to compare the performance of the main PAH risk assessment methods used nowadays. We used a harmonized data set of patients from several PAH trials. First, we built a new version of the Pulmonary Hypertension Outcome and Risk Assessment (PHORA) model, (PHORA 2.1) using random forest and Bayesian network analysis, as previously published. In the same data set, the Registry to Evaluate Early and Long‐term PAH Disease Management (REVEAL) 2.0 and REVEAL Lite 2.0, 3‐ and 4‐strata Comparative Prospective Registry of Newly Initiated Therapies for Pulmonary Hypertension (COMPERA) 2.0 and noninvasive French Pulmonary Hypertension Registry (FPHR) risk assessment methods were applied. The discrimination performance to predict 1‐year survival was compared between all these methods using the area under the curve (AUC) after fivefold cross‐validation and the Wilcoxon signed rank test. A data set of 494 PAH patients was used for this analysis (mean age 47 ± 18 years old, 77% female, 62% idiopathic or heritable PAH, mean pulmonary artery pressure 53 mmHg). The PHORA 2.1 method included 13 variables, with an AUC of 0.84 compared with 0.71, 0.78, 0.72, 0.81 and 0.80 for noninvasive FPHR, 4‐stata and 3‐strata COMPERA, REVEAL 2.0 and REVEAL Lite 2.0, respectively, after the fivefold cross‐validation. In general, the 95% confidence interval AUC of PHORA 2.0 covers higher values in general, with a Wilcoxon signed rank test significantly higher compared with all the other methods (*p* < 0.001). The PHORA 2.1 model, based on a Bayesian network analysis, depicted the highest AUC to predict the outcome, compared with other PAH risk assessment methods widely used.

## A090 PULMONARY VASCULAR RESISTANCE THRESHOLDS OF 3, 5, AND 10 WU ARE ASSOCIATED WITH SIGNIFICANTLY DIFFERENT SURVIVAL IN PULMONARY HYPERTENSION WITH CHRONIC LUNG DISEASE

K Dwivedi, R Lewis, S Alabed, A Swift, D Kiely, R Condliffe

*Department of Infection, Immunity & Cardiovascular Disease, University of Sheffield, Medical School, Beech Hill Road, Sheffield S10 2RX; Pulmonary Vascular Disease Unit, Royal Hallamshire Hospitals, Sheffield Teaching Hospitals NHS Trust, Sheffield S10 2JF, UK*

The 6th World Symposium on Pulmonary Hypertension (WSPH) suggested a new haemodynamic definition of precapillary pulmonary hypertension (PH): mean pulmonary artery pressure (mPAP) >20 mmHg and pulmonary vascular resistance (PVR) ≥ 3 WU. In PH secondary to chronic lung disease (PH‐CLD), recent studies have shown a prognostic difference, with significantly different mortality in patients with PVR > 5 WU. The aim was to quantify the impact of different PVR thresholds on mortality in PH‐CLD in a large patient cohort with baseline right heart catheterization. All patients diagnosed with PH‐CLD between February 2001 and January 2019 were identified in the ASPIRE registry. Patients were grouped into singular thresholds of PVR (2–3, 3–4, 4–5…) and survival was compared using Kaplan–Meir (KM) curves and pairwise log‐rank tests. Thresholds were explored until there was a significant (*p* < 0.05) pairwise log‐rank difference between each group. Locally weighted scatterplot smoothing (LOWESS) analysis was performed to identify PVR thresholds with 20% and 40% 1‐year mortality. Three hundred and eighty‐four patients with a diagnosis of PH‐CLD, chronic obstructive pulmonary disease or ILD, mPAP >20 mmHg and pulmonary arterial wedge pressure ≤15 mmHg were identified. Initial exploration of the KM curve and pairwise log‐rank group comparisons identified three thresholds, 3, 5 and 10 WU, with a significant (*p* < 0.05) difference in between‐group survival. Five‐year survival was 54, 26, 13% and 5.1% in the 0–3, 3–5, 5–10 and 10+ WU group, respectively. LOESS analysis identified 2.6 and 6.7 WU as thresholds for 20% and 40% 1‐year mortality, respectively. Survival in patients with PVR 2–3 was significantly better than in patients with PVR 3–4. In conclusion, the severity of PVR at baseline in PH‐CLD is an important prognostic predictor. Groups divided by PVR thresholds of 3, 5 and 10 WU have significantly different survival in PH‐CLD.

## A091 FIBROSIS ON COMPUTED TOMOGRAPHY IS AN INDEPENDENT PREDICTOR OF MORTALITY IN PULMONARY HYPERTENSION WITH CHRONIC LUNG DISEASE

K Dwivedi, M Sharkey, M Mamalakis, R Lewis, S Alabed, S Rajaram, C Hill, J Wild, D Kiely, R Condliffe, A Swift

*Department of Infection, Immunity & Cardiovascular Disease, University of Sheffield, Medical School, Beech Hill Road, Sheffield S10 2RX; 3DLab, Sheffield Teaching Hospitals NHS Trust, Sheffield S5 7 AU; Department of Computer Science, University of Sheffield, Sheffield S1 4DP; Pulmonary Vascular Disease Unit, Royal Hallamshire Hospitals, Sheffield Teaching Hospitals NHS Trust, Sheffield S10 2JF; Department of Radiology, Sheffield Teaching Hospitals NHS Trust, Sheffield S5 7 AU, UK*

Computed tomography (CT) imaging is routinely used to assess lung disease severity in group 3 pulmonary hypertension with chronic lung disease (PH‐CLD). Emphysema and fibrosis are the two most common disease patterns, but their prognostic importance is not well understood.

The aim was to quantify impact of moderate/severe CT emphysema and fibrosis, independent of demographics and pulmonary vascular resistance (PVR). All patients diagnosed with PH‐CLD between February 2001 and January 2019 were identified from the ASPIRE registry. CTs were reported by specialist PH radiologists and disease severity graded semi‐quantitatively as none, mild, moderate or severe. For analysis, none/mild and moderate/severe were grouped. CT reports were searched with a regex‐string‐search algorithm; results were validated manually, blinded to clinical data. Multivariate Cox regression adjusted for age, sex and PVR. Kaplan–Meier curves were compared using the log‐rank test. Analysis was repeated for the subgroup of severe PH‐CLD, defined as mean pulmonary artery pressure ≥35 mmHg and cardiac index <2 L/min/m^2^. Two hundred and seventy‐eight patients were identified (191 severe PH‐CLD). In PH‐CLD, 29% had moderate/severe fibrosis, which was a significant univariate [hazard ratio (HR) 1.69, 1.25–2.27, *p* < 0.001] and multivariate (HR 1.42, 1.04–1.95, *p* = 0.033) predictor for poor survival. Survival was significantly different between moderate/severe and none/mild fibrosis (*p* = 0.00046). In severe PH‐CLD, 27% had moderately severe fibrosis, which was a significant univariate (HR 1.72, 1.16–2.57, *p* = 0.007) but not a significant multivariate predictor (HR 1.43, 0.94–2.16, *p* = 0.10). Survival, however, remained significantly different between moderate/severe and none/mild fibrosis (*p* = 0.006). Emphysema was not prognostic in either group. Moderate or severe fibrosis on chest CT is associated with poor survival, even after adjusting for age, sex and pulmonary vascular resistance, in PH‐CLD. Further quantitative analysis is warranted before the use of CT features as biomarkers.

## A092 THE THERAPEUTIC POTENTIAL OF OMEGA‐3 FATTY ACIDS AND IT'S DERIVATIVES IN PULMONARY HYPERTENSION

Julia Schäffer, Mareike Gierhardt, Poonam Sarode, Oleg Pak, Kathrin Malkmus, Simone Kraut, Jonas Münsk, Nils Schupp, Ilker Kanbagli, Sylvia Reiche, H Ardeschir Ghofrani, Friedrich Grimminger, Werner Seeger, Ralph T Schermuly, Norbert Weissmann, Rajkumar Savai, Matthias Hecker, Natascha Sommer

*Department of Internal Medicine, Universities of Giessen and Marburg Lung Center (UGMLC), Member of the German Center for Lung Research (DZL), Excellence Cluster Cardio‐Pulmonary Institute (CPI), Justus‐Liebig University, Giessen, Germany; Instituto de Investigación en Biomedicina de Buenos Aires (IBioBA) – CONICET – Partner Institute of the Max Planck Society, Buenos Aires, Argentina; Department of Lung Development and Remodeling, Max Planck Institute for Heart and Lung Research, Bad Nauheim, Germany, Institute for Lung Health (ILH), Giessen, Germany*

Pulmonary hypertension (PH) is characterized by pulmonary vascular remodeling that leads to right heart insufficiency. Pulmonary vascular remodeling is promoted by perivascular inflammatory cell infiltration. Resolvin E1 (5 S, 12 R, 18R‐ trihydroxyeicosa‐pentaenoic acid; RvE1), derived from an omega‐3 fatty acid (n‐3 FA) is an anti‐inflammatory and pro‐resolving lipid mediator that might affect inflammatory cell infiltration, but also pulmonary arterial smooth muscle cell (PASMC) proliferation. Pulmonary hypertension was either initiated by exposure of wild‐type (WT) and transgenic fat‐1 mice (which generate n‐3 FA) to hypoxia or by injection of WT rats with monocrotaline. In both models, the WT rodents were treated with RvE1, its precursor, 18‐hydroxyeicosapentaenoic acid (18‐HEPE), or placebo after induction of PH. Development of PH was quantified by echocardiography, hemodynamic in vivo measurements and histological analysis. Proliferation of human PASMCs incubated with conditioned media of macrophages (CMM) and RvE1 was determined by incorporation of bromodeoxyuridine. Furthermore, the effect of RvE1 on pulmonary vasoconstriction was measured in isolated, ventilated and perfused mouse lungs. Wild‐type mice exposed to hypoxia and treated with RvE1 or 18‐HEPE showed significantly lower right ventricular systolic pressure (RVSP) and pulmonary vascular remodeling compared with placebo‐treated mice, whereas fat‐1 mice showed only attenuated RVSP. Rats injected with monocrotaline and treated with RvE1 also showed reduced RVSP, better right heart function and less CD68^+^ cell infiltration. Moreover, RvE1 treatment did not directly affect pulmonary vasoconstriction or PASMC proliferation but attenuated increased proliferation of human PASMCs incubated with CMM. Therapeutic treatment with RvE1 (and its precursor, 18‐HEPE) attenuated PH in different rodent models, most probably by affecting inflammatory cell infiltration. Further studies to investigate the underlying protective mechanism of RvE1 in PH and the contribution of perivascular inflammation need to be performed.

## A093 THE Β_3_‐ADRENERGIC TARGET IN PULMONARY ARTERIAL HYPERTENSION

Susana F Rocha, Monika Spaczynksa, Anabel Diaz‐Guerra, Álvaro Macías, Mónica Gómez, Ana García‐Alvarez, Borja Ibáñez, Eduardo Oliver

*Centro Nacional de Investigaciones Cardiovasculares (CNIC) Carlos III, Centro Nacional de Investigaciones Cardiovasculares (CNIC) Carlos III CIBER de Enfermedades Cardiovasculares (CIBERCV), Centro de Investigaciones Biológicas Margarita Salas (CIB‐CSIC), Madrid; Institut Clínic Cardiovascular‐Hospital Clínic, IDIBAPS, Barcelona; IIS‐Hospital Fundación Jiménez Díaz, Madrid, Spain*

The role of β_3_‐adrenergic receptor (β_3_‐AR) in the heart and vessels has been studied widely, and the use of β_3_‐agonists has been proposed as a therapeutic strategy in certain cardiovascular diseases. Pulmonary arterial hypertension (PAH) is originated by an aberrant vascular remodelling characterized by endothelial dysfunction and vascular cell proliferation. These changes lead to an increase in pulmonary arterial pressure, followed by right ventricular hypertrophy, heart failure and premature death. To clarify the role of β_3_‐AR in PAH and whether it might be a potential therapeutic target, we have used two animal models: hypoxia‐ and monocrotaline‐induced PAH in mice and rats, respectively. In addition, we have analysed both β_3_‐AR knockout and transgenic mice, with conditional cell‐specific restoration of β_3_‐AR expression in a β_3_‐AR knockout background. We found that loss of β_3_‐AR aggravates the PAH phenotype, and its restoration in endothelial cells (ECs), but not in cardiomyocytes or in vascular smooth muscle cells (SMCs), leads to an ameliorated pathophysiology. This amelioration is reflected in a decrease in right ventricular systolic pressure, right ventricular hypertrophy, arterial remodelling, SMC proliferation and a recovered endothelial dysfunction (reflected by a decreased ectopic von Willebrand factor expression and normalized NO production). Accordingly, pharmacological activation of β_3_‐AR with mirabegron, both in mice and rats also leads to better hemodynamic and pathophysiological parameters. *In vitro* experiments with human pulmonary artery ECs and SMCs demonstrate that activation of endothelial β_3_‐AR induces NO production, which acts indirectly on SMCs to regulate vasodilation and proliferation. Concurrently, pharmacological activation of β_3_‐AR ceases to have any beneficial effect in endothelial NO synthase knockout mice exposed to hypoxia. Additionally, the β_3_‐AR regulates levels of reactive oxygen species and has a role in controlling endothelial cellular stress and mitochondrial fitness. In conclusion, the β_3_‐AR stands as a new therapeutic target for the treatment of PAH and other pulmonary vascular diseases in which endothelial dysfunction plays a relevant role.

## A094 IMPLICATION OF HISTONE ACETYLTRANSFERASE P300 IN RIGHT HEART FAILURE ASSOCIATED WITH PULMONARY ARTERIAL HYPERTENSION

A Bourgeois, S E Lemay, Y Grobs, T Shimauchi, S Martineau, G Mkannez, S Breuils‐Bonnet, F Potus, S Provencher, O Boucherat, S Bonnet,

*Quebec Heart and Lung Institute Research Center, Laval University, Quebec City, QC, Canada*

Pulmonary arterial hypertension (PAH) is defined by increased pulmonary artery pressure, leading to progressive hypertrophy of the right ventricle (RV). At first, RV hypertrophy allows adaptation to increased resistance, necessary to maintain a normal cardiac output (CO). With time, maladaptive remodeling takes place, leading ultimately to RV failure. Although significant progress has been made, the molecular mechanism driving this transition towards maladaptive hypertrophy remains elusive. Histone acetyltransferase P300 has been identified as a central player in various pathological processes, such as proliferation/apoptosis and hypertrophy/fibrosis, all of which are crucial features of RV remodeling in PAH. Given its role in controlling gene transcription programs, we hypothesized that P300 might contribute to pathological RV remodeling and failure in PAH. We demonstrated by western blot (WB) and immunofluorescence (IF) that P300 expression is increased in compensated and decompensated RV from PAH patients (*n* = 10–19, *p* < 0.05), monocrotaline (MCT)‐treated rats (*n* = 5–12, *p* < 0.05) and rats subjected to pulmonary artery binding (PAB; *n* = 5–12, *p* < 0.05). P300 upregulation was observed in cardiomyocytes (CMs) and fibroblasts (RVfbs; by IF and WB). Using in vitro CM models (H9C2 cells and rat neonates), we show that P300 inhibition using CCS‐1477 prevents phenylephrine‐induced hypertrophy (*n* = 5, *p* < 0.05). Additionally, P300 inhibition decreased cell surface area in CMs isolated from adult MCT‐treated rats. In PAH‐RVfbs, P300 inhibition reduced proliferation and survival (Ki67, PCNA and Survivin), activation (pSMAD2/3 and aSMA) and production of extracellular matrix proteins (Fn, Col1A and MMP2). *In vivo* administration of CCS‐1477 significantly improved RV functions in MCT‐treated rats (right ventricular systolic pressure, *p* < 0.01; CO, *p* < 0.01). This was associated with a decrease in histone acetylation in the RV (WB H3K27ac, *p* < 0.01; H3ac, *p* < 0.01; H4ac, *p* < 0.05) and reduced fibrosis (Masson Trichrome staining, *p* < 0.05). These results are being confirmed in the PAB rat model. Our findings identified P300 as a new therapeutic target for PAH that could improve associated cardiac dysfunction.

## A095 CRISPR/CAS9‐MEDIATED GENE EDITING OF NOXO1 REVERSED CHRONIC CIGARETTE SMOKE‐INDUCED PULMONARY HYPERTENSION AND EMPHYSEMA

Z I Kanbagli, C Garcia Castro, J M Schäffer, S Hadzic, M Gredic, R T Schermuly, W Seeger, N Weissmann, N Sommer, O Pak

*Department of Internal Medicine, Universities of Giessen and Marburg Lung Center (UGMLC), Member of the German Center for Lung Research (DZL), Excellence Cluster Cardio‐Pulmonary Institute (CPI), Justus‐Liebig University, Giessen, Germany, Department of Lung Development and Remodeling, Max Planck Institute for Heart and Lung Research, Bad Nauheim, Germany; Department of Internal Medicine, Universities of Giessen and Marburg Lung Center (UGMLC), Member of the German Center for Lung Research (DZL), Excellence Cluster Cardio‐Pulmonary Institute (CPI), Justus‐Liebig University, Giessen, Germany*

Chronic obstructive pulmonary disease (COPD) is a severe, progressive disease with a poor prognosis, characterized by airway remodelling and emphysema development, which can be induced by cigarette smoke exposure. Previously, we have identified upregulation of the NADPH oxidase organizer (*NoxO1*) as a key player for development of murine cigarette smoke‐induced emphysema and pulmonary hypertension (PH). Thus, we applied the CRISPR/Cas9‐mediated genome editing system to knock out the *NoxO1* gene as a therapeutic strategy for treatment of smoke‐induced emphysema and PH. *In vitro* CRISPR/Cas9‐mediated gene editing downregulated mRNA expression of *NoxO1* and inhibited the cigarette smoke extract (CSE)‐induced decrease of cellular viability of mouse lung epithelial cells (MLE12). Adeno‐associated viral (AAV) vector‐mediated deletion of *NoxO1* by CRISPR/Cas9 constructs significantly diminished the effect of 10% CSE exposure on cellular viability and late apoptosis/necrosis of precision‐cut lung slices, which were determined by Alamar Blue assay and propidium iodide immunostaining, respectively. Lastly, after smoke exposure of wild‐type mice for 8 months, intratracheal application of AAV‐mediated deletion of *NoxO1* by CRISPR/Cas9 system was performed. Three months after intratracheal delivery of AAV‐CRISPR‐Cas9 constructs, *NoxO1* deletion diminished the established alteration of lung function and PH, which were detected by histological assessment, hemodynamic measurements and lung function tests. Together with our previous results on the preventive effect of *NoxO1* knockout on chronic cigarette smoke‐induced emphysema, CRISPR/Cas9‐mediated genome editing of *NoxO1* might be a potential and promising therapeutic strategy to treat cigarette smoke‐induced PH and emphysema.

## A096 VESPER, A NOVEL LNCRNA, REGULATES RIGHT VENTRICULAR HYPERTROPHY IN RESPONSE TO PRESSURE OVERLOAD

Jordy Marinus Maria Kocken, Pedro Mendes Ferreira, Raquel Figuinha Videira, Adelino Leite Moreira, Paula A Da Costa Martins,

*Department of Molecular Genetics, Faculty of Sciences and Engineering, Maastricht University, 6229 ER Maastricht, The Netherlands; CARIM School for Cardiovascular Diseases, Faculty of Health, Medicine and Life Sciences, Maastricht University, 6229 ER Maastricht, The Netherlands; Unic@RISE, Department of Surgery and Physiology, Faculty of Medicine, Cardiovascular Research and Development Centre, University of Porto, Al. Prof. Hernâni Monteiro, 4200‐319 Porto, Portugal; Paris–Porto Pulmonary Hypertension Collaborative Laboratory (3PH), UMR_S999, INSERM, Université Paris‐Sarclay, F‐94270 Le Kremlin‐Bicêtre, France*

Pulmonary artery hypertension (PAH) is a chronic disease leading to progressive right ventricular (RV) remodeling followed by RV failure. With no cure available, current treatment strategies focus on reducing endothelial dysfunction, lowering PA pressure and maintaining cardiac output. Much is known on left ventricle (LV) remodeling, but it appears that RV remodeling follows distinct molecular pathways that are poorly understood. To identify novel mechanisms triggering RV remodeling, we subjected mice to 4 weeks of increased RV pressure overload through pulmonary artery banding (PAB) surgery or sham surgery as a control, isolated the RV and performed RNA sequencing on the tissue. When focusing on the differentially expressed long Noncoding RNA (lncRNA), we identified Gm38399, now called Vesper (right ventricle specific pressure overload), as the most consistently upregulated lncRNA after PAB. After RT‐qPCR validation of the RNA‐seq data, we also analyzed murine LV tissue of mice that underwent transaortic constriction (TAC) to induce LV pressure overload. Although we observed similar baseline levels of expression in physiological conditions, no changes in Vesper expression in the LV after TAC were detected. These results suggest that this lncRNA is specifically upregulated in the RV in response to pressure overload. To understand the role of Vesper in RV hypertrophy, we investigated neighboring genes and observed that Pkp1 was downregulated while Phlda3 and Nav1 were upregulated in RV tissue of PAB mice compared with sham. *In vitro*, in HL‐1 cells, we were able to silence Vesper with a gapmer and saw Pkp1 expression upregulation and downregulation of Nav1 and Phlda3. We are currently performing in vivo studies in mice subjected to either PAB or sham surgery, and inhibiting Vesper with a specific gapmer to assess the contribution of this lncRNA to RV physiology and remodeling, identify its underlying molecular pathways and, hopefully, unravel its potential as a therapeutic target for PAH.

## A097 CHARACTERIZATION OF PATIENTS WITH CHRONIC THROMBOEMBOLIC DISEASE BY USING CARDIOPULMONARY EXERCISE TESTING

*Abdul G. Hameed, Ian Smith, Andrew J. Swift, David G. Kiely, Charlie A. Elliot, Robin Condliffe, Alex M. Rothman, A. A. Roger Thompson, Ian Sabroe, Mark Anthony Sammut, Athanasios Charalampopoulos*

*Pulmonary Vascular Disease Unit, Cardiology Department, Sheffield Teaching Hospital NHS Foundation Trust, Sheffield; The University of Sheffield, Sheffield, UK*

Data on functional characterization of patients with chronic thromboembolic disease (CTED) with the use of cardiopulmonary exercise testing (CPET) are scanty. We performed CPET in 35 patients with CTED but no pulmonary hypertension (PH). We compared the results with 17 patients with chronic thromboembolic disease and PH (CTEPH; mean pulmonary artery pressure ≥25 mmHg) who also underwent a CPET. All patients underwent a CT pulmonary angiogram, a cardiac MRI/MR angiogram and a right heart catheterization. The *P*(A–a)O_2_ and *V*_D_/*V*_T_ were measured based on an ABG at peak exercise. There was no difference in age, body mass index or sex distribution between the groups, but the CTEPH group had a higher mean pulmonary artery pressure and pulmonary vascular resistance than the CTED group. The CTED patients showed a trend towards a higher peak O_2_ consumption. There was no difference in parameters of cardiac performance, such as anaerobic threshold, *V*O_2_/workload, O_2_ pulse or chronotropic index. There was no difference in ventilator efficiency indices, such as *V*_E_/*V*CO_2_ and *P*_ET_CO_2_, between the two groups. However, CTED patients had significantly lower dead‐space ventilation with a lower *P*(A–a)O_2_ and less O_2_ desaturation compared with the CTEPH group. Peak lactate was also higher in CTED patients. Additional contributors to reduced exercise capacity/exercise termination in the CTED group were: chronotropic incompetence (6%), airways obstruction or restriction (20%), deconditioning (20%) and dysfunctional breathing (6%). Patients with CTED did not differ from patients with CTEPH in cardiac performance or ventilatory efficiency CPET parameters. Patients with CTED had significantly lower dead‐space ventilation and less O_2_ desaturation than patients with CTEPH. Patients with CTED showed a trend towards a higher peak O_2_ consumption than patients with CTEPH. Other comorbidities often contribute to exercise intolerance in patients with CTED.

## A098 LONGITUDINAL ^129^XE AND ^1^H LUNG MRI ASSESSMENT OF LUNG STRUCTURE, VENTILATION, PERFUSION, AND GAS TRANSFER FOLLOWING HOSPITALIZATION WITH COVID‐19

Laura C Saunders, Guilhem J Collier, Ho‐Fung Chan, Paul J C Hughes, Laurie J Smith, James Watson, James Meiring, Zoë Gabriel, Thomas Newman, Megan Plowright, James A Eaden, Jody Bray, Helen Marshall, David J Capener, Leanne Armstrong, Jennifer Rodgers, Martin Brook, Alberto M Biancardi, Madhwesha R Rao, Graham Norquay, Oliver Rodgers, Ryan Munro, Neil J Stewart, Gisli Jenkins, James Grist, Fergus Gleeson, Fred Wilson, Tony Cahn, Andy J Swift, Smitha Rajaram, Gary H Mills, Lisa Watson, Paul J Collini, Rod Lawson, A A Roger Thompson, Jim M Wild

*Department of Infection, Immunity and Cardiovascular Disease, University of Sheffield, Sheffield; Sheffield Teaching Hospitals NHS Foundation Trust, Sheffield, UK, National Heart and Lung Institute, Imperial College London; Department of Radiology, Oxford NHS Foundation Trust, Oxford, UK; GlaxoSmithKline, Stevenage, UK*

The long‐term effects of coronavirus disease 2019 (COVID‐19) pneumonia on the lungs and pulmonary circulation require further characterization. We assessed progression of pathophysiological pulmonary changes during 1 year of follow‐up of patients who had been hospitalized because of COVID‐19. After discharge, recruited patients had up to four MRI examinations at a median of 6 (*n* = 9), 12 (*n* = 9), 25 (*n* = 7) and 52 (*n* = 3) weeks. Lung MRI examinations included: ultra‐short echo time (UTE), dynamic contrast‐enhanced (DCE) lung perfusion, ^129^Xe diffusion weighted (DW‐MRI), ^129^Xe ventilation and ^129^Xe 3D dissolved phase imaging. Nine patients (age 56 ± 9 years; six male) were recruited. Ventilation defect percentage and whole lung coefficient of variation of lung ventilation decreased significantly at 25 weeks (visit 3) compared with visit 1 at 6 weeks (*p* = 0.010 and *p* = 0.048). The UTE imaging indicated no evidence of lung scarring, and DW‐MRI indicated normal lung microstructure across all visits. Dissolved phase xenon imaging showed that RBC:TP increased significantly at visits 2 and 3 compared with visit 1 (*p* = 0.048). Median RBC:TP was abnormally low at all visits compared with reference age‐ and sex‐matched data. An individual's RBC:TP was associated significantly and positively with an increase in their pulmonary blood volume (*p* = 0.026). For patients with 52‐week data available, one showed a continued improvement in RBC:TP; however, two of the patients maintained a low RBC:TP. In patients recovering from COVID‐19, xenon gas transfer improves alongside pulmonary blood volume. Further work is needed to establish the proportion of post‐COVID‐19 patients who have longer‐term impairment in xenon transfer and to correlate changes in lung MRI parameters with symptoms, lung function tests and other imaging modalities. Persistent impairment of xenon transfer might represent a physiological mechanism underlying ongoing symptoms in some patients and might indicate damage to the pulmonary microcirculation.

## A099 PHASE 1 STUDIES TO ASSESS INHALED SERALUTINIB AS A PERPETRATOR OR A VICTIM OF DRUG–DRUG INTERACTIONS IN HEALTHY SUBJECTS

Jack Li, Ed Parsley, Matt Cravets, Anita Mathias

*Gossamer Bio, Inc., San Diego, CA, USA*

Seralutinib is an inhaled, small molecule kinase inhibitor that targets PDGFRα/β, CSF1R and c‐KIT pathways that play an important role in the pathological progression of pulmonary arterial hypertension (PAH). Seralutinib is designed specifically to maximize the therapeutic index by directly targeting diseased pulmonary arterioles and reducing systemic exposure. The effects of seralutinib as a perpetrator (alters exposure of co‐administered drugs) of drug–drug interactions (DDIs) have been reported previously^1^. This study expands the evaluation of seralutinib as a victim (co‐administered drugs alter exposure of seralutinib) of DDIs. Seralutinib is currently being examined in a phase 2 trial (TORREY; NCT04456998). To evaluate the potential for seralutinib as a perpetrator, 24 healthy adults received a cocktail of probe substrates: caffeine (CYP1A2), montelukast (CYP2C8), flurbiprofen (CYP2C9), midazolam (CYP3A), digoxin (P‐gp) and pravastatin (OATP1B1/1B3), with or without seralutinib. To evaluate the potential for seralutinib as a victim, 19 healthy adults received seralutinib with or without the strong CYP3A inhibitor itraconazole or the weak CYP3A inhibitor fosaprepitant. Pharmacokinetics (PK), including peak plasma concentration (*C*_max_) and area under the plasma–concentration time curve (AUC), and safety endpoints were evaluated. The effect of the moderate CYP3A inhibitor erythromycin on the PK of seralutinib was assessed with static mechanistic modeling. Seralutinib coadministration increased midazolam AUC threefold and caffeine AUC by 33%, indicating moderate CYP3A and weak CYP1A2 inhibition, respectively. Seralutinib slightly inhibited P‐gp, with digoxin *C*_max_ increased by 28%. Seralutinib is neither an inhibitor nor an inducer of CYP2C8, CYP2C9 and OATP1B1/1B3. Fosaprepitant and itraconazole increased seralutinib AUC by 8% and 84%, respectively. Erythromycin was predicted to increase seralutinib AUC by 25–39%. Seralutinib was well tolerated; no serious adverse events or adverse events leading to drug withdrawal or early termination were reported. Inhaled seralutinib demonstrated a favorable DDI profile and can be co‐ administered with most medications, including PAH background therapies.

## A100 A FIRST MULTICENTRE, ADAPTIVE RECALL‐BY‐GENOTYPE STUDY IN PULMONARY ARTERIAL HYPERTENSION: STUDY PROTOCOL

Emilia M Swietlik, Benjamin Dunmore, Christopher J Rhodes, Alessandro Ruggiero, Matina Prapa, Elaine Soon, Daniel Greene, Carmen Treacy, Martin R Wilkins, Nicholas W Morrell

*Department of Medicine, University of Cambridge, School of Clinical Medicine, Cambridge, UK; National Heart and Lung Institute, Faculty of Medicine, Imperial College London, London, UK; Royal Papworth Hospital, NHS Foundation Trust, Radiology Department, Cambridge, UK; St George's University Hospitals NHS Foundation Trust, London, UK; Department of Genetics and Genomic Sciences, Icahn School of Medicine at Mount Sinai, New York, NY, USA; NIHR BioResource – Rare Diseases Consortium (NBR‐RD), National Cohort Study of Idiopathic and Heritable PAH (PAH Cohort), International Consortium for Genetic Studies in Pulmonary Arterial Hypertension (PAH ICON), National Heart and Lung Institute, Faculty of Medicine, Imperial College London, London, UK*

Although pulmonary arterial hypertension (PAH) is traditionally considered a monogenic condition with incomplete penetrance, it remains unexplained in >70% of patients without a positive family history. Recent research found new rare and common disease variants and epigenetic changes associated with PAH. Here, we propose an adaptive methodology within a recall‐by‐genotype (RbG) framework using detailed phenotyping to describe established and new genetic pathways in an attempt to estimate their contribution to PAH. Participants of the NBR‐RD, PAH Cohort study and PAH ICON, in addition to their relatives and healthy controls, will be recalled based on genotype or genetic, epigenetic or proteomic risk scores. The continuous analysis of ‘‐omic’ datasets will guide further recruitment to sequential test groups. The deep phenotyping will be customized to each test group and will include any of the following: detailed personal and family history, physical examination, collection of blood, urine, cells and tissues for biochemical and ‘‐omic’ studies, invasive and noninvasive cardiovascular and pulmonary testing, imaging and qualitative research. The Phenopackets schema, which supports the exchange of computable longitudinal case‐level phenotypic information, will be used to collect, share and compute over phenotype data. The design of this adaptive RbG study offers efficiency (lower cost and higher definition of phenotyping in comparison to population studies) in the assessment of associations between new genomic findings and detailed PAH phenotypes. The possibility to add test groups in response to the new genetic findings will streamline the recruitment and will allow filling of the gap caused by shortcomings of in vitro and animal testing. This specific adaptive RbG design aims to uncover the influence of rare and common variants in addition to epigenetic changes on the PAH phenotype, and it is the first study of this type in pulmonary arterial hypertension.

## A101 DEPTOR DOWNREGULATION BY HIF/DEC1 AXIS LEADS TO MTOR ACTIVATION IN PULMONARY VASCULATURE

Shruthi Devkumar, Estela Rodríguez, Peter Barnes, Veronica Carroll,

*St George's University of London, London; Imperial College London, London, UK*

Tissue hypoxia commonly occurs in lung diseases, such as chronic obstructive pulmonary disease (COPD), which causes structural and phenotypic changes in cells of the pulmonary vasculature, leading to remodelling of vessels. Vascular remodelling can, ultimately, result in pulmonary hypertension (PH) and right ventricular heart failure. The phenotypic changes that occur in chronic hypoxia to pulmonary artery vascular smooth muscle cells (PAVSMCs) are orchestrated by the transcription factors, hypoxia‐inducible factors, HIF‐1 and HIF‐2. The HIFs regulate genes involved in increased cell proliferation, migration and metabolism, but the complexity of altered gene expression that leads to vascular remodelling in PH is still poorly understood. In this study, we investigated HIF‐mediated mechanisms of vascular remodelling in pulmonary vasculature. We found that HIFs can activate mechanistic target of rapamycin (mTOR) signalling through downregulion of the mTOR inhibitor, DEPTOR, in PAVSMCs. mTOR is a key mediator of vascular remodelling in PH. Further studies revealed that the transcriptional repressor DEC1 (BHLHe40) is a HIF‐regulated gene involved in DEPTOR downregulation. Furthermore, DEPTOR was abundantly expressed in normal rat and mouse pulmonary arteries and human PAVSMCs but was absent in pulmonary vessels of patients with PH associated with COPD. Taken together, our data show that the HIF/DEC1 axis downregulates DEPTOR in hypoxia, which might contribute to vascular remodelling and PH by activation of mTOR signalling. Our work helps to further our understanding of the pathology of PH, which might help to uncover new therapeutic targets for this disease.

## A102 SEX DIFFERENCES IN RIGHT VENTRICULAR FUNCTION AS DETERMINED BY STRAIN ECHOCARDIOGRAPHY IN MULTIPLE PULMONARY HYPERTENSION ETIOLOGIES

Felipe Kazmirczak, Sasha Z Prisco, Thenappan Thenappan, Kurt W Prins

*Cardiovascular Division, Lillehei Heart Institute, Department of Medicine, University of Minnesota, Minneapolis, MN, USA*

Sex differences in right ventricular (RV) function, defined predominantly by imaging modalities, are present in multiple etiologies of pulmonary hypertension (PH). Strain echocardiography is an emerging imaging modality that evaluates myocardial dynamics with increased sensitivity over traditional echocardiography, but the impact of biological sex on RV strain imaging in PH is not well defined. We evaluated 217 pulmonary arterial hypertension (PAH) (60 males and 157 females) and 233 group 3 patients (105 males and 128 females) from the University of Minnesota PH registry. The RV free wall and RV global longitudinal strain was determined using TomTec software. We defined the relationship between RV strain and pulmonary vascular resistance (PVR) based on sex and PH type to understand how PH etiology and biological sex influenced RV function. Male group 3 patients had lower mean pulmonary arterial pressure (mPAP; 40 ± 10 vs. 47 ± 16 mmHg; *p* = 0.007) and no difference in PVR (6.5 ± 3.4 vs. 7.5 ± 4.2 WU; *p* = 0.17) in comparison to PAH males. Female group 3 PH patients had less severe PH compared with female PAH patients (mPAP 42 ± 12 mmHg and PVR 7.2 ± 3.8 WU vs. mPAP 44 ± 13 mmHg and PVR 9.5 ± 5.8 WU; *p* = 0.13 and *p* = 0.002, respectively). Females had better RV function than males as quantified by both RV free wall strain and RV global longitudinal strain in both PH etiologies. RV–PA coupling, estimated by RV strain relative to PVR, was less impaired in females in both populations. Interestingly, there were no significant differences in strain rates or estimated RV–PA coupling when PH etiology was evaluated in both males and females. Females have superior RV function and RV–PA coupling compared with their male counterparts independent of PH etiology as quantified by strain echocardiography.

## A103 INTERMITTENT FASTING RESTRUCTURES RIGHT VENTRICULAR LIPID METABOLISM AND MICROTUBULES IN RODENT PULMONARY ARTERIAL HYPERTENSION

Felipe Kazmirczak, Sasha Z Prisco, Megan Eklund, Lynn Hartweck, Kurt W Prins

*Cardiovascular Division, Department of Medicine, University of Minnesota Medical School, Minneapolis, MN; Lillehei Heart Institute, University of Minnesota Medical School, Minneapolis, MN, USA*

Intermittent fasting (IF) is a well‐documented lifespan extender via pleotropic mechanisms, including AMP‐kinase activation. AMP‐kinase regulates lipid metabolism and the microtubule cytoskeleton, and both these key biological processes contribute to right ventricular (RV) failure in pulmonary arterial hypertension (PAH). Here, we show that IF restructures both mitochondrial and peroxisomal lipid metabolism in the RV of rodent PAH. Concomitant with these findings, IF restores mitochondrial and peroxisomal density. Intermittent fasting normalizes levels of electron transport chain (ETC) protein abundance and activity. Importantly, echocardiographically and hemodynamically defined measures of RV function are positively associated with the abundance of fatty acid oxidation proteins and ETC proteins in correlational heatmapping analyses. Finally, IF prevents pathological microtubule remodeling, which corrects t‐tubule derangements. In summary, our study shows that IF counteracts impaired RV lipid metabolism and pathological microtubule remodeling and suggests that it might be a novel treatment approach for RV failure, a currently lethal and untreatable consequence of PAH.

## A104 MICROBIOME‐DERIVED SHORT‐CHAIN FATTY ACID BUTYRATE ATTENUATES PULMONARY VASCULAR ENDOTHELIAL INFLAMMATORY ACTIVATION AND PULMONARY HYPERTENSION

J Andres Pulgarin, Jacob Dubner, Imad Al Ghouleh,

*Division of Medicine, Division of Cardiology, and the Pittsburgh Heart, Lung and Vascular Medicine Institute, University of Pittsburgh, Pittsburgh, PA, USA*

Pulmonary hypertension (PH) is a progressive, severe disease characterized by high blood pressure in the pulmonary circulation, inflammatory cell infiltration and excessive pulmonary vascular remodeling. Endothelial cell (EC) dysfunction is increasingly recognized as a precipitating event for this remodeling. Short‐chain fatty acids (SCFAs) present in circulating blood and produced solely by the host microbiome (MB) have been associated with benefit in cardiovascular diseases and hypertension. However, there is a dearth of knowledge of the role of MB‐derived molecules in PH‐ECs. We hypothesized that butyrate (a bacterial SCFA) plays a protective role on ECs under PH. In our results, supplementation of mice with butyrate [sodium butyrate (NaB)] attenuated hypoxia‐induced right ventricular (RV) pressure (from 43 ± 2 to 35 ± 3 mmHg, *p* < 0.05) and hypertrophy (Fulton index from 0.43 ± 0.008 to 0.38 ± 0.01, *p* < 0.05) in vivo. Sodium butyrate also lowered hypoxia‐induced circulating monocyte numbers (*p* < 0.05) and reduced gene expression in hypoxic lung CD31^+^ ECs of the inflammatory adhesion molecule VCAM‐1 and increased expression of ERM binding phosphoprotein 50 (EBP50), a protein we have shown to modulate endothelial reprogramming in PH (qRT‐PCR, *p* < 0.05). *In vitro*, NaB attenuated the PH‐related inflammatory cytokine IL1b‐induced human pulmonary arterial EC (HPAEC) migration at 48 h (*p* < 0.001). Consistent with in vivo results, butyrate also reversed IL1b‐induced increase in VCAM‐1 expression at the mRNA and protein levels in HPAECs (qRT‐PCR and western blot, *p* < 0.0001 and *p* < 0.05, respectively). Moreover, butyrate rescued the IL1b‐induced reduction in cell surface protein integrin a3 (*p* < 0.01) and EBP50 (*p* < 0.05) expression. Collectively, our results demonstrate that butyrate attenuates PH, potentially by protecting against myeloid cell induction and pathophysiological pro‐inflammatory, promigratory pulmonary vascular EC activation. Butyrate might also reduce EC reprogramming through its effects on EBP50. These findings support the potential for therapeutic benefits of SCFAs in PH and the need for further investigation of the role of microbiome‐derived metabolites in this devastating disease.

## A105 OUTCOMES OF COVID‐19 IN PULMONARY HYPERTENSION PATIENTS

Ana Paula S Oliveira, Raíssa K Dantas, Amanda T Campoy, Juliana Lucena, Thaís C F Menezes, Andrei A A Cordeiro, Melliane Daud, Angelo F X Cepeda, Roberta P, Ramos, Rudolf K F Oliveira (PVRI Member), Eloara V M Ferreira (PVRI Member), Jaquelina S Ota‐Arakaki

*Federal University of São Paulo, Unifesp/EPM, SP, Brazil*

Pulmonary hypertension (PH) is a severe disease that can progress to clinical decompensation, risk of hospitalization and death owing to disease‐related or other diseases. In the context of coronavirus disease 2019 (COVID‐19), PH was considered a risk factor for complications. The purpose of the study was to assess the mortality rate of COVID‐19 in PH patients from a PH Center in Brazil. We conducted a telephone survey between June and August 2021 among all patients or relatives from the PH referral center who were followed after the first case of COVID‐19 in Brazil. Only patients with a confirmed diagnosis of PH were included in the analysis. Of the 426 patients followed in the first 18 months of the pandemic, 115 patients were excluded (lost to follow‐up, post‐acute PE or unconfirmed PH). Among 311 patients included, 39 had a confirmed diagnosis of COVID‐19 (COVID‐19 + ), and 38.5% of patients were hospitalized. The estimated incidence rate was 12.5%. Comparing the COVID‐19+ versus patients without infection (COVID‐19 − ) in the period, the mean age was similar (55 ± 17 vs. 54 ± 16 years) and the majority in the COVID‐19+ group were female (85% vs. 69%, *p* = 0.039), respectively. There was no difference in the proportion of patients diagnosed with pulmonary arterial hypertension (PAH; 49% and 42%) and chronic thromboembolic pulmonary hypertension (CTEPH; 24% and 33%) between groups. All PAH patients and the majority of CTEPH patients were treated on specific therapy (combination/triple therapy, 70%). The case fatality rate in the PH‐COVID‐19+ group was 23%. Considering only PAH and CTEPH, the case fatality rate was 21,9%, while COVID‐19 mortality was 2.9% and overall lethality in Brazil was 2.8%. In the COVID‐19+ group, the mean pulmonary artery pressure was 48 ± 14 mmHg, cardiac index 2.7 ± 0.6 L/min/m^2^ and pulmonary vascular resistance 730 ± 424 dyn.s/cm^5^. In conclusion, among PH patients there was high incidence and mortality from COVID‐19, even in those with PH‐specific therapy. Further studies are needed to evaluate the prognostic predictors in PH‐COVID‐19 patients.

## A106 RODATRISTAT ATTENUATES PROLIFERATION OF HUMAN AND RAT PULMONARY ARTERIAL SMOOTH MUSCLE CELLS IN MODELS OF PULMONARY ARTERIAL HYPERTENSION

Michelle Palacios, Howard Lazarus, Stephen Wring

*Altavant Sciences, Inc., Cary, NC, USA*

Rodatristat ethyl is a prodrug for rodatristat, a peripherally restricted tryptophan hydroxylase 1 (TPH1) inhibitor in clinical development to evaluate the impact of reducing serotonin elevations associated with pulmonary arterial smooth muscle cell (PASMC) proliferation in group 1 pulmonary arterial hypertension (PAH). Rodatristat lowers mean pulmonary artery pressure and reverses vascular remodeling in monocrotaline and sugen–hypoxia rat PAH models. Here, we discuss in vitro and in vivo nonclinical data that characterize the ability of rodatristat to reduce PASMC proliferation. *In vitro* proliferation of PASMCs (by 5‐bromo‐2‐deoxyuridine incorporation) was compared following exposure to conditioned media after incubation with human PA‐endothelial cells (PAECs) isolated from healthy and idiopathic PAH patient‐derived lung tissues. Monocrotaline‐challenged rats were treated for 7, 14 or 28 days with 200 mg/kg/day rodatristat ethyl. Longitudinal endpoints included: right ventricular (RV) hypertrophy, histopathology, serotonin levels and cell proliferation [by immunohistochemistry staining for proliferating cell nuclear antigen (PCNA)]. Clinically relevant levels of rodatristat (0.1–1.0 µM) elicited a dose‐dependent reduction (maximal effect −41%, *p* < 0.0001, *n* = 3) in PASMC proliferation for conditioned media from idiopathic PAH PAECs. Healthy controls demonstrated lower effect (−19%, *n* = 3), consistent with reported increased TPH1 expression in PAH PAECs. In rats, rodatristat reduced: RV hypertrophy [RV/(left ventricle + septum), −23%, *n* = 6, day 28 versus monocrotaline vehicle treatment], vessel wall thickness (−39%, *p* < 0.0001, *n* = 3) and serum serotonin (~−79%, days 7–28). Reductions in lung serotonin and PASMC proliferation (as %PCNA^+^ cells/mm^2^) lagged serum, achieving significance on day 28 (−83%, *n* = 6, *p* < 0.001; and −34%, *p* < 0.01, *n* = 3, respectively). Rodatristat inhibited proliferation of human PASMCs exposed in vitro to idiopathic PAH PAEC‐conditioned media and in rat PASMCs after monocrotaline challenge. Across the treatment periods, the in vivo time course for improved histopathology and reductions in PCNA^+^ cells tracked reductions in lung serotonin. The data support inhibition of serotonin biosynthesis as a promising treatment to attenuate the PASMC proliferation characteristic of PAH.

## A107 SELECTIVE INHIBITION OF HISTONE DEACETYLASE 1 AND 2 ATTENUATES PULMONARY VASCULAR REMODELING IN EXPERIMENTAL PULMONARY HYPERTENSION

Kondababu Kurakula, Xiao‐Qing Sun, Quint A J Hagdorn, Diederik E Van der Feen, Frances S de Man, Rolf M F Berger, Harm Jan Bogaard, Sébastien Bonnet, Marie‐José Goumans

*Department of Cell and Chemical Biology, Leiden University Medical Center, Leiden, The Netherlands; Department of Pulmonary Medicine, Amsterdam Cardiovascular Sciences, Amsterdam UMC, Vrije Universiteit Amsterdam, Amsterdam, The Netherlands; Center for Congenital Heart Diseases, Department of Pediatric Cardiology, Beatrix Children's Hospital, University Medical Center Groningen, University of Groningen, The Netherlands; Pulmonary Hypertension and Vascular Biology Research Group of Quebec Heart and Lung Institute, Laval University, Quebec, Canada*

The expression and activity of histone deacetylases (HDACs) are increased in pulmonary arterial hypertension (PAH). Although inhibition of HDACs has been promoted as a treatment for PAH, results from preclinical studies were equivocal and raised concerns regarding safety. Quisinostat is a ‘second generation’ HDAC inhibitor, which is highly potent against class I and II HDACs and elicits a prolonged pharmacodynamic response. We hypothesized that quisinostat might exert therapeutic effects in experimentally induced PAH. HDAC1 expression was measured in lungs and microvascular endothelial cells (MVECs) from control donors and PAH patients. HDAC activity, proliferation and inflammation were measured after quisinostat treatment of PAH MVECs. Chronic quisinostat treatment was tested in three PAH rat models: the sugen + hypoxia (SuHx), the monocrotaline shunt flow (MF) and pulmonary artery banding (PAB) models. HDAC1 expression is increased in lungs of PAH patients, where it regulates transforming growth factor‐β (TGFβ) signalling. Quisinostat decreased proliferation and mitigated inflammatory cytokine expression in MVECs. Conditioned medium from quisinostat‐treated PAH MVECs inhibited the growth of healthy pulmonary artery smooth muscle cells. Oral treatment with quisinostat reversed vascular remodeling and improved pulmonary hemodynamics in SuHx‐PAH and MF‐PAH rats by reducing proliferation, perivascular inflammation and TGFβ signalling. Quisinostat could be combined safely with contemporary PAH standard of care. Quisinostat treatment also supported the pressure‐loaded right ventricle in PAB rats. Quisinostat attenuates proproliferative and pro‐inflammatory pathways, potentially through inhibition of TGFβ signalling. Quisinostat reversed pulmonary vascular remodeling in diverse PAH rat models and supported the pressure‐loaded right ventricle in the PAB rat model. Altogether, these data demonstrate that quisinostat could be a promising intervention for PAH.

## A108 EFFECT OF DIGOXIN USE ON MORTALITY IN PULMONARY ARTERIAL HYPERTENSION: A SINGLE‐CENTER EXPERIENCE

Kevin Y Chang, Katherine Giorgio, Rob F Walker, Pamela L Lutsey, Kurt W Prins, Marc Pritzker, Thenappan Thenappan

*Cardiovascular Division, Department of Medicine, University of Minnesota, Minneapolis, MN, USA; Division of Epidemiology and Community Health, School of Public Health, University of Minnesota, Minneapolis, MN, USA*

Digoxin increases cardiac output modestly in acute settings in patients with pulmonary arterial hypertension (PAH) and right ventricular failure; however, very little is known about the effects of chronic digoxin therapy in PAH. We studied 251 patients with PAH from the Minnesota Pulmonary Hypertension Repository, a prospective registry that enrolls all consecutive patients treated at the University of Minnesota. Person‐time began at the date of PAH diagnosis. Date of digoxin prescription was recorded. Primary endpoint was all‐cause mortality. Mortality hazard ratios (HRs) were calculated using Cox proportional hazards regression, with digoxin use as a time‐dependent covariate, adjusted for baseline patient characteristics. The mean age was 55 ± 15 years, and 73% were women. Sisty‐seven (27%) patients were treated with digoxin. When compared with those who were not treated with digoxin, patients on digoxin were more likely to be younger (52 vs. 56 years; *p* = 0.07), had idiopathic PAH (28 vs. 17%; *p* = 0.06), on warfarin therapy (22 vs. 13%; *p* = 0.07) and had worse hemodynamics: higher right atrial pressure (11 vs. 8 mmHg; *p* < 0.001), higher mean pulmonary artery pressure (51 vs. 44 mmHg; *p* = 0.001), higher pulmonary vascular resistance (11.1 vs. 7.7 WU; *p* < 0.001) and lower cardiac index (2.0 vs. 2.6 L/min/m^2^). During a median follow‐up time of 4.5 years, there were a total of 95 deaths (27 in digoxin group vs. 68 in no digoxin group). Digoxin use was associated with higher mortality both with and without adjusting for the baseline characteristics [crude HR 1.83 (95% confidence interval: 1.17–2.86); Adjusted HR 2.26 (95% confidence interval: 1.36–3.76)]. Digoxin is used more often in patients with severe idiopathic PAH and is associated with increased mortality even after adjusting for the severity of PAH and right heart failure. Future prospective studies should assess the safety/efficacy of chronic digoxin use in PAH.

## A109 REMOTE 6‐MIN WALK TESTING IN PULMONARY HYPERTENSION PATIENTS

Tess LaPatra, Grayson L Baird, Diane Pinder, Maeve Gaffney, James R Klinger, Harold I Palevsky, Jason Fritz, Christopher J Mullin, Steven Pugliese, Kerri Akaya Smith, Jeremy A Mazurek, Mary E Whittenhall, Nadine Al‐Naamani, Steven M Kawut, Corey E Ventetuolo

*Department of Medicine, Perelman School of Medicine at the University of Pennsylvania, Philadelphia, PA, USA; Lifespan Biostatistics Core, Lifespan Hospital System, Providence, PI, USA; Departments of Diagnostic Imaging, Medicine and Health Services, Policy and Practice, Brown University, Providence, RI, USA*

The 6‐minute walk test (6MWT) evaluates therapeutic response and prognosis in pulmonary arterial hypertension (PAH). We sought to determine the feasibility, safety and accuracy of performing the 6MWT in nonclinical indoor/outdoor settings (‘remote’). We conducted a two‐center study of PAH and chronic thromboembolic pulmonary hypertension (CTEPH) patients. We enrolled World Health Organization class I–III patients on stable PH therapy. Within a 6‐week period, subjects completed a 6MWT in clinic, a ‘practice’ 6MWT in a remote location and a formal 6MWT in the same remote location. Subjects either masked (or did not) for all three 6MWTs; a subset performed an additional walk with the opposite masking status. The 6MWTs were completed on a 30 m, flat, straight course. During remote walks, an adult companion counted laps and a study coordinator timed the 6MWT via telephone. Generalized mixed modeling was used to examine vital signs, Borg scores and 6‐min walk distance (6MWD) between settings. We used Deming regression to model outcome concordance. Twenty‐nine subjects enrolled; the study sample included 25 subjects. The average age was 51 years, and 72% were female. Twelve per cent had CTEPH. Post‐6MWT heart rate, oxygen saturation and Borg scores for breathlessness and fatigue were neither clinically nor statistically significantly different between in‐clinic and remote settings (all *p* > 0.20). There was no evidence of systematic differences on average 6MWD between in‐clinic and remote settings (480 m, 95% confidence interval 443–520 vs. 483 m, 95% confidence interval 445–523, *p* = 0.96). There was strong concordance between in‐clinic and remote walk outcomes. There were three nonserious adverse events. Remote 6MWTs were similar to in‐clinic tests in heart rate, oxygen saturation and Borg scores at test end and distance walked. Larger studies in different geographic settings are necessary to assess the safety and validity of remote 6MWTs for clinical or research monitoring.

## A110 GCN2 DEFICIENCY ENHANCES DSRNA‐INDUCED INFLAMMATION IN VASCULAR ENDOTHELIAL CELLS

Helena Turton, Allan Lawrie, Roger Thompson, Max Schwiening, Elaine Soon

*Department of Infection, Immunity and Cardiovascular Disease, University of Sheffield, Sheffield, UK; Cambridge Institute for Medical Research, University of Cambridge, Cambridge, UK*

Pulmonary veno‐occlusive disease (PVOD) is a rare cause of pulmonary hypertension (PH) associated with biallelic mutations in *EIF2AK4*, which encodes GCN2. GCN2 is one of four integrated stress response (ISR) kinases that phosphorylate eIF2α to suppress translation globally. GCN2 also upregulates specific ISR‐associated transcription factors; however, its role in PVOD is unclear. Another ISR kinase, EIF2AK2 (PKR), senses double‐stranded RNA (dsRNA), a known regulator of vascular remodelling. We hypothesized that GCN2 deficiency alters dsRNA‐induced inflammation via altered ISR kinase activity. The aim was to investigate responses to dsRNA in GCN2‐deficient endothelial cells. GCN2 and/or PKR were knocked down in healthy donor blood outgrowth endothelial cells (BOECs) and/or hPAECs using siRNA. Cells were treated with synthetic dsRNA [poly(I:C) 25 μg/mL] for 24 h. The RNA was harvested for qPCR analysis. Supernatant cytokines [tumor necrosis factor‐α (TNF‐α) and interleukin‐6 (IL‐6)] were quantified by Luminex technology. CellTitre‐Glo® assays assessed cell viability. Mouse embryonic fibroblasts were generated from *GCN2* knockout mice and littermate controls. Deficiency of GCN2 in poIy(I:C)‐treated BOECs and hPAECs significantly increased TNF‐α release compared with the control (mean ± SEM: siCTL PAECs 22.8 ± 9.2 pg/mL/1,000 cells vs. siGCN2 PAECs 94.0 ± 10.5 pg/mL/1,000 cells, *p* = 0.03 by ANOVA, *n* = 5 experimental repeats from three donors) but had no significant effect on IL‐6 release. Double knockdown of GCN2 and PKR partly attenuated enhanced TNF‐α release, but induction of PKR by poly(I:C) was not fully suppressed by siRNA. GCN2 deficiency significantly reduced viability of poIy(I:C)‐treated hPAECs compared with nontargeting siRNA control. Constitutive GCN2 deficiency in MEFS was associated with an exaggerated activation of the ISR. GCN2 deficiency enhanced TNF‐α release from endothelial cells in response to dsRNA and reduced PAEC viability. Future work will investigate whether these phenotypes are mediated via ISR activation, as observed in GCN2‐deficient fibroblasts, and assess whether stable knockdown of PKR fully suppresses enhanced TNF‐α release in dsRNA‐stimulated GCN2‐deficient cells.

## A111 DEEP LEARNING OF REGULATORY ELEMENTS IDENTIFIES NONCODING GENOTYPE‐PHENOTYPE ASSOCIATIONS PREDISPOSING TO PAH

Tobias Tilly, Kathryn Auckland, Reshma Nibhani, Jennifer Martin, Nicholas W Morrell, Pietro Liò, Stefan Gräf

*Department of Medicine, University of Cambridge, Addenbrooke's Hospital, Cambridge; Department of Computer Science and Technology, Cambridge; NIHR BioResource for Translational Research, Cambridge Biomedical Campus, Cambridge; Department of Haematology, University of Cambridge, Cambridge Biomedical Campus, Cambridge CB2 0PT, UK*

Pulmonary arterial hypertension (PAH) is a rare and fatal lung disease. In only one‐third of patients the cause can be attributed to rare genetic variation in the protein‐coding space. The sequencing of 15,000 whole genomes by the NIHR BioResource – Rare Diseases (NBR), including >1,200 PAH samples, provides an unprecedented opportunity to estimate the contribution of regulatory genome variation to the development of PAH. Here, we aim to determine whether sequence‐based predictions of epigenetic features can be used to narrow down the possible regions of interest and allow aggregation of variants into functional groups for association testing. Deploying artificial intelligence strategies, a convolutional neural network has been trained using publicly available datasets to predict epigenetic features from DNA sequences. The model was tested against known enhancer regions, and its accurate performance was verified. Two approaches were developed for the evaluation of the epigenetic features: (1) an epigenetic importance score that supplies general information about the availability of epigenetic profiles within a region to explore the noncoding space; and (2) a regulation score that combines the predicted features into activating and repressing subsets for more detailed analyses to gauge the regulatory impact of variants. Based on the regulatory impact and other commonly used annotations, variants were filtered and aggregated for overrepresentation analysis comparing cases with controls. In conclusion, preliminary statistical analysis revealed likely disease‐causing sequence variation in a *BMPR2* enhancer. Here, we present an extended search into enhancer networks associated with PAH, unlocking the regulatory noncoding space for translation into genomic medicine.

## A112 QUANTIFICATION OF SPATIAL HETEROGENEITY OF ARTERIAL SMALL VESSEL VOLUME LOSS IN CHRONIC THROMBOEMBOLIC PULMONARY HYPERTENSION

Farbod N Rahaghi, Pietro Nardelli, Andetta R Hunsaker, Aaron B Waxman, George R Washko, Raúl San José Estépar,

*Division of Pulmonary and Critical Care; Department of Radiology, Brigham and Women's Hospital, Harvard Medical School, Boston, MA, USA*

Computed tomography (CT) imaging plays a crucial role in the diagnosis and treatment of chronic thromboembolic pulmonary hypertension (CTEPH). Prior investigations have demonstrated loss of small vessel volume, proximal arterial dilation and increased arterial tortuosity in both PAH and CTEPH. In this study, we set out to quantify the spatial heterogeneity of small vessel distribution in patients with CTEPH and PAH compared with controls. Subjects with dyspnea but no cardiopulmonary disease by iCPET (controls) and those with CTEPH and PAH with CT angiography were identified from the Brigham and Women's Hospital right heart catheterization registry (IRB#2018P000419). The vasculature was reconstructed and separated into arteries and veins using the Chest Imaging platform (www.chestimagingplatform.org). Vessel segments were labeled in 3 × 3 quadrants (upper, middle, lower, anterior, middle and posterior), leading to nine equal volume segments in each lung (18 per subject). Small vessels were those with a cross‐sectional area of <5 mm^2^. Statistical analysis was performed with R v.3.6. Comparisons were made with the Wilcoxon rank‐sum test. Volumes are vessel volumes (in milliliters) normalized by lung volume (in liters). The coefficient of variation was defined as standard deviation divided by the mean. Twenty‐one CTEPH, 47 PAH and 40 control patients were used for this analysis. There was a reduction in median arterial small vessel volume in both PAH and CTEPH compared with controls [CTEPH, 12.8 (10.0–13.6), *p* < 0.00001; PAH, 12.9 (10.3–14.4), *p* = 0.00001; control, 15.4 (13.9–17.1)], with no difference between PAH and CTEPH (0.29). However, measures of arterial heterogeneity, including range, standard deviation and, particularly, coefficient of variation, were higher in CTEPH in comparison to both controls and PAH [CTEPH, 0.23 (0.19–0.31), *p* < 0.00001 with controls, *p* = 0.002 with PAH; PAH, 0.16 (0.13–0.23), *p* = 0.0007; control, 0.13 (0.11–0.16)]. In this cohort of patients with CTEPH, automated CT‐based regional heterogeneity of loss of small vascular volume was a marker that distinguished CTEPH both from controls and from PAH patients.

## A113 CORONARY ARTERY COMPRESSION IN A PATIENT WITH CHRONIC THROMBOEMBOLIC PULMONARY HYPERTENSION

Hamza Zafar, Abdul Hameed, Jennifer T. Middleton, Sarah K Binmahfooz, Christian Battersby, David Kiely, Andrew J Swift, Oliver Watson, Alexander M K Rothman

*Department of Infection, Immunity & Cardiovascular Disease, Faculty of Medicine, Dentistry & Health, University of Sheffield, Royal Hallamshire Hospital, Beech Hill Road, Sheffield S10 2RX, UK; Pulmonary Vascular Disease Unit, Sheffield University Teaching Hospitals NHS Trust, Royal Hallamshire Hospital, Sheffield S10 2JF, UK*

Chronic thromboembolic pulmonary hypertension (CTEPH) is a condition that results in increased pulmonary vascular resistance and pulmonary artery pressure and causes right heart failure. Exercise limitation in patients with CTEPH can be attributable to pulmonary vascular disease, right heart failure, coronary compression or an alternative diagnosis. A 61‐year‐old female with a history of CTEPH not amenable to pulmonary endarterectomy or balloon angioplasty owing to distal disease, low web‐burden and multiple comorbidities, including systemic hypertension, was admitted from the pulmonary hypertension clinic because of ongoing chest pain and a marked reduction in exercise capacity. ECG demonstrated lateral T wave inversion, and troponin‐I was elevated, at 80 ng/L. The cardiac MRI demonstrated severely impaired left ventricular ejection fraction, and the CT pulmonary angiography showed a significantly dilated pulmonary artery. To determine the cause of chest pain, coronary angiography and intravascular ultrasound (IVUS) were undertaken, revealing severe left main stem (LMS) stenosis in the absence of atherosclerosis. A multiprofessional team discussion concluded that symptoms were likely to be attributable to external compression of the LMS by the dilated main pulmonary artery and, owing to lesion location and co‐morbidities, percutaneous coronary intervention (PCI) would be an appropriate strategy. Successful PCI was undertaken via the right radial artery with a single 4.5 mm × 18 mm drug‐eluting to the LMS, with good apposition demonstrated by IVUS. Aspirin, ticagrelor and apixaban were given for 1 month, followed by ticagrelor and apixaban long term. At 6 months, clinical condition and symptoms were markedly improved, as indicated by an increase in cardiac MRI‐measured left ventricular ejection fraction from 36% to 67%, N‐terminal pro B‐type natriuretic peptide from 788 to 219 pg/mL and World Health Organization functional class from IV to II. Although uncommon, LMS compression in patients with pulmonary hypertension represents a treatable cause of chest pain, exercise limitation and heart failure.

## A114 EXTRACELLULAR NICOTINAMIDE PHOSPHORIBOSYLTRANSFERASE IS A THERAPEUTIC TARGET TO ADDRESS RIGHT VENTRICULAR DYSFUNCTION IN PULMONARY ARTERIAL HYPERTENSION: PRECLINICAL RAT RESCUE STUDIES AND GWAS NAMPT PROMOTER SNP ANALYSES IN IDIOPATHIC PULMONARY ARTERIAL HYPERTENSION

Mohamed Ahmed, Nancy G Casanova, Franz Rischard, Heidi E Erickson, Stan Miele, Michael W Pauciulo, Tae‐Hwi Schwantes‐An, William Nichols, Nathan Ellis, Jason X‐J Yuan, Ankit A Desai, Joe G N Garcia

*Department of Pediatrics; Department of Medicine, University of Arizona Health Sciences, Tucson, AZ; Department of Molecular and Medical Genetics; Department of Medicine, Indiana University School of Medicine, Indianapolis, IN; Aqualung Therapeutics Corp. Tucson AZ; Division of Human Genetics, Cincinnati Children's Hospital Medical Center and Department of Pediatrics, University of Cincinnati College of Medicine, Cincinnati, OH, USA*

Pulmonary arterial hypertension (PAH) is a fatal disease crucially influenced by both dysregulated inflammatory pathways and genetic variation. The pool of genes known to contribute to idiopathic PAH (iPAH) severity, however, is limited, and effective anti‐inflammatory PAH strategies remain an unmet need. Six published/unpublished lines of evidence highlight the involvement of circulating extracellular nicotinamide phosphoribosyltransferase (eNAMPT) in human PH pathobiology. First, we show that eNAMPT is a damage‐associated molecular pattern protein (DAMP) that ligates Toll‐like receptor 4 (TLR4). Second, *NAMPT* RNA and protein expression are significantly increased in peripheral blood mononuclear cells (PBMCs) and in remodeled lung vessels from PAH patients. Third, plasma eNAMPT levels are elevated in PAH subjects and correlated with cardiac catheer‐proven right ventricular (RV) dysfunction. Fourth, a PAH BioBank genome‐wide association study (GWAS; 674 iPAH subjects and 250 controls) identifies three *NAMPT* single‐nucleotide polymorphisms (SNPs; rs61330082, rs9770242 and rs59744560), previously linked to inflammatory injury severity, including acute respiratory distress syndrome mortality, to exhibit highly significant associations with indices of RV dysfunction and PAH severity, including cardiac output, peripheral vascular resistance (PVR) and RV stroke work index. Fifth, our compellingly preclinical PAH rat studies (monocrotaline, hypoxia/Sugen) demonstrate that eNAMPT is a highly druggable target with the humanized eNAMPT‐neutralizing mAb, ALT‐100 (half‐life 12–14 days), profoundly attenuating PAH vascular remodeling and indices of RV dysfunction. Lastly, genomic studies validate ALT‐100 targeting of the eNAMPT/TLR4 pathway in rat lung tissues and human PAH PBMCs as the mechanism by which ALT‐100 mAb rescues PAH severity and halts PAH progression. Together, these results strongly validate *NAMPT* as a novel PAH candidate gene and support ALT‐100 mAb as a potential PAH therapy to address the unmet need for strategies to improve RV dysfunction/failure and PAH survival. Future PAH clinical trials designed to assess eNAMPT as a novel PAH therapeutic target might use plasma eNAMPT levels and/or *NAMPT* SNP stratification to enhance the likelihood of therapeutic success.

## A115 ^68^GA‐DOTATATE PET‐CT IMAGING IN PULMONARY ARTERIAL HYPERTENSION

Taylor Covington, Hyeon Ju Song, Aaron B Waxman, Mark Southwood, Nicholas W Morrell, F Nick Rahaghi, Marcelo DiCarli, Jean‐Marie Bruey, Larry Zisman, Heather Jacene, Paul B Yu

*Cardiovascular Research Center, Massachusetts General Hospital and Harvard Medical School, Boston, MA, USA; Pulmonary and Critical Care Medicine; Cardiology, Nuclear Medicine and Molecular Imaging, Brigham and Woman's Hospital and Harvard Medical School, Boston, MA, USA; Pulmonary Vascular Diseases Unit, Papworth Hospital and Cambridge University, Cambridge, UK; Addenbrookes Hospital, Cambridge University, Cambridge, UK; Clinical Development, Gossamer Bio, San Diego, CA, USA*

Inflammation is thought to be a contributor to pulmonary arterial hypertension (PAH). We hypothesized that macrophage‐mediated inflammation might be detected in pulmonary tissues of PAH using a PET‐CT imaging modality with the radionuclide‐labeled somatostatin analog probe ^68^Ga‐DOTAtate. Approved for the diagnosis of neuroendocrine tumors, ^68^Ga‐DOTAtate PET‐CT has also been used experimentally for detecting macrophage‐mediated inflammation in myocardial and lung tissues. We enrolled seven patients with WSPH group 1 PAH and 18 age‐ and gender‐matched non‐diseased controls for ^68^Ga‐DOTAtate PET‐CT imaging. Lung parenchymal standardized uptake values (SUVs) were determined in volumes of interest (VOIs) sampled from the left and right upper and lower lobe, averaged, and normalized to blood pool VOIs in the descending thoracic aorta. Normalized ^68^Ga‐DOTAtate SUV ratios were compared with invasive hemodynamic parameters, echocardiographic right ventricular systolic pressure (eRVSP), functional class, etiology of PAH and number of PAH medications. Normalized ^68^Ga‐DOTAtate SUV ratios were dichotomous in PAH patients and healthy subjects, whereby nearly all control subjects and three of seven PAH patients had lung SUVs ≤ 1, consistent with no net enrichment of uptake in lung parenchyma versus the blood pool. The remaining four patients had markedly elevated SUV ratios of 5.3‐ to 6.6‐fold the mean of control SUV ratios. Linear regression revealed trends correlating SUV ratios with pulmonary vascular resistance and eRVSP. The SUV ratios were significantly higher in patients on two or three PAH medications but were not different between idiopathic PAH and APAH‐CTD subjects. Uptake of ^68^Ga‐DOTAtate was markedly increased in the lungs of subjects with PAH receiving multiple versus single PAH drug therapy, and low in subjects without disease. As a noninvasive imaging biomarker that might correspond to disease severity and, potentially, inflammation, ^68^Ga‐DOTAtate PET‐CT could be useful for predicting or assessing responses to anti‐inflammatory therapies in PAH.

## A116 COMPARISON OF THE FOUR‐STRATA COMPERA 2.0 WITH REVEAL LITE 2.0 IN PATIENTS WITH PULMONARY ARTERIAL HYPERTENSION: A SINGLE CENTER RETROSPECTIVE ANALYSIS

Sandeep Sahay, Frederick Wang, Duc T Nguyen, Edward A Graviss

*Houston Methodist Lung Center, Houston, TX; Texas A&M University, College Station, TX; Department of Pathology and Genomic Medicine, Houston Methodist Hospital, Houston, TX, USA*

The 2016 ESC/ERS guidelines recommended risk assessments in the management of pulmonary arterial hypertension (PAH). COMPERA 2.0 further refined risk assessment with a four‐strata approach. In this analysis, we compared the four‐strata COMPERA 2.0 with REVEAL Lite 2.0. We carried out retrospective analysis of data from our referral pulmonary hypertension center at the Houston Methodist Lung Center, Houston, TX, USA. Only idiopathic PAH (IPAH), heritable PAH (HPAH) and drug‐toxin‐related PAH patients were included. Patients were stratified in four‐strata risk groups as per COMPERA 2.0, and three strata (low, intermediate and high) using REVEAL lite 2.0 at baseline. One hundred and seven patients were included (83% females), with a median age of 48 years. The three‐strata COMPERA classified 43.0% as low risk, 54.2% as intermediate risk and 2.8% as high risk. The four‐strata COMPERA 2.0 classified 32.7% as low risk, 36.5% as low‐intermediate risk, 28.0% as high‐intermediate risk and 2.8% as high risk. REVEAL Lite 2.0 classified 53.2% as low risk, 23.4% as intermediate risk and 23.4% as high risk. Using the four‐strata approach, the 1‐, 3‐ and 5‐year event‐free survival in the low‐risk group was 100% and in the low‐intermediate risk group it was 94.7, 94.7% and 91.1%, respectively. The 1‐, 3‐ and 5‐year event‐free survival in the high‐intermediate risk group was 100, 95.0% and 89.7%, respectively. Using the three‐strata, the 5‐year survival in the low‐risk group was 100%. The 1‐, 3‐ and 5‐year survival in the intermediate‐risk group was 96.4, 93.9% and 88.7%, respectively. The 1‐ and 3‐year survival in the high‐risk group was 66.7% and 33.3%, respectively. Using the REVEAL Lite 2.0, 1‐, 3‐ and ‐5‐year event‐free survival in each group was: low‐risk, 98.3, 98.3% and 96.2%; intermediate‐risk: 95.7, 95.7% and 90.0%; and high‐risk: 96.0, 84.7% and 84.7%, respectively. Seventy per cent of the intermediate‐high risk group were high risk according to REVEAL Lite 2.0. COMPERA 2.0 was better able to identify an intermediate‐high‐risk group compared with the three‐strata approach. The intermediate‐high‐risk group of four‐strata performed in a similar manner to the high‐risk group of REVEAL Lite 2.0.

## A117 HEART RATE VARIABILITY IN PULMONARY ARTERIAL HYPERTENSION

J Minhas, T Lapatra, J Hseih, M Pipke, N Al‐Naamani, A Smith, H Palevsky, J Mazurek, S Pugliese, J Fritz, S Kawut

*Division of Pulmonary, Allergy and Critical Care, Division of Cardiology, University of Pennsylvania, Philadelphia, PA, USA; PhysIQ, Chicago, IL, USA*

Patients with PAH cannot increase their stroke volume during exertion and are dependent on heart rate (HR) to increase cardiac output. These patients have increased sympathetic and reduced vagal tone, resulting in higher baseline HR. We sought to study the association of HR variability (HRV) with right ventricular systolic function and activity in PAH. We studied data from 15 patients at the University of Pennsylvania who wore the VitalPatch wireless biosensor, which continuously captures activity and HR in 60‐s epochs for 5 days consecutively. We collected tricuspid annular plane systolic excursion (TAPSE) data from clinical echocardiograms performed within 6 months of device wear. We used linear mixed effect models with a random intercept to assess the relationship between TAPSE and HRV and between HRV with daily step counts. We defined HR variability based on the variation of the R–R interval (time domain method) whereby subjects with shorter R–R intervals had higher HRV and those with longer R–R intervals had lower HRV. The median age was 56 years; 73% were women, and idiopathic PAH was the most common etiology. A 1 cm increase in TAPSE was associated with reduction in the R–R interval (β = 15 ms, *p* = 0.03; i.?e. higher HRV). A 100 ms reduction in the R–R interval (higher HRV) was associated with increasing step counts (β = 6 steps/min, *p* = 0.001). Both models were adjusted for the mean daily HR of the patiens. Using a functional principal components analysis, we found that two distinct temporal HR patterns explained >80% of the variability in our data: (1) those with sustained increase/decrease in HR; and (2) a single mid‐day peak in HR and return to population mean later in the day. Higher HRV was associated with better TAPSE and higher daily step counts. The HRV could serve as a biomarker of disease severity and as a mechanistic surrogate endpoint in PAH.

## A118 SEX DIFFERENCES IN RIGHT ATRIAL REMODELING IN PATIENTS WITH PULMONARY ARTERIAL HYPERTENSION

Danny Mohama, Pitchaya Worapongsatitaya, Felipe Kazmirczak, Kurt Prins, Marc R Pritzker, Thenappan Thenappan

*Cardiovascular Division, Department of Medicine, University of Minnesota, Minneapolis, MN, USA*

Females with pulmonary arterial hypertension (PAH) have better right ventricular (RV) function and survival compared with males. However, it is unknown whether there are sex differences in right atrial (RA) remodeling and function in patients with PAH. We studied 180 patients with PAH from the Minnesota Pulmonary Hypertension Repository, a prospective registry that enrolls all consecutive patients treated at the University of Minnesota. Baseline characteristics, hemodynamics and echocardiographic parameters were compared between females and males. Multiple linear regression was used to determine the difference in RA remodeling and function between males and females after adjusting for the severity of pulmonary vascular disease and RV function. There were 137 (76.1%) females in our cohort. At baseline, compared with males, females were significantly older (median age 59 vs. 51 years, *p* = 0.04), more often had idiopathic PAH (32 vs. 5%, *p* < 0.01), had a lower 6‐min walk distance (311 vs. 430 m, *p* < 0.01), higher serum N‐terminal pro‐brain natriuretic peptide (929 vs. 303 pg/dL, *p* < 0.01), lower cardiac index (2.1 vs. 2.4 L/min/m^2^, *p* < 0.01) and higher pulmonary vascular resistance (PVR; 9 vs. 5.9 WU, *p* < 0.01). Male patients with PAH had a significantly higher RAEDVI after adjusting for age, body mass index, PVR, RVEDP and RVFAC (β = 7.3, 95% confidence interval 0.7–14.0, *p* = 0.030). There was no difference in RA area index (11.6 vs. 10.7 cm^2^/m^2^, *p* = 0.40) and RA ejection fraction (40 vs. 46%, *p* = 0.37) between female and male patients with PAH. Female patients with PAH have superior RA remodeling with reduced adverse structural changes in comparison to male patients with PAH.

## A119 APELIN SIGNALING PROTECTS AGAINST PULMONARY ARTERY BANDING‐INDUCED RIGHT VENTRICULAR FAILURE

Bakhtiyor Yakubov, Marjorie Albrecht, Amanda Fisher, Todd Cook, Valerie Nadeau, Steeve Provencher, Sebastien Bonnet, Tim Lahm, Andrea L Frump

*Division of Pulmonary, Critical Care, Sleep and Occupational Medicine, Department of Medicine, Indiana University, Indianapolis, IN, USA; Pulmonary Hypertension Research Group, University Institute of Pneumology and Cardiology of Quebec, Laval University, Quebec, QC, Canada; Division of Pulmonary, Critical Care and Sleep Medicine, National Jewish Health, Denver, CO, USA*

Right ventricular (RV) function is the major determinant of survival in pulmonary arterial hypertension (PAH); however, no RV‐directed therapies exist. Apelin is a secreted peptide that plays a crucial role in cellular homeostasis. However, the role of apelin signaling during the progression of RV failure remains unclear. Apelin‐mediated signaling protects against RV failure. Apelin and apelin receptor (APLNR) were quantified (western blot) and immunolocalized in RVs from PAH patients. *In vivo*, RV failure was induced in male and female rats by pulmonary artery banding (PAB) for 10 days or 10 weeks. A subset of PAB rats were treated with Pyr‐Apelin‐13 (200 μg/kg/day) or an APLNR antagonist, ML221 (2 mg/kg/day). Pressure–volume loops, RV function and molecular changes were assessed. *In vitro* characterization of apelin signaling was performed in RV endothelial cells (RVECs) and cardiomyocytes (RVCMs) isolated from rats. A value of *p* < 0.05 was considered statistically significant. In PAH RVs, apelin and APLNR localized to RVCMs and RVECs and were decreased versus controls (*p* < 0.05). *In vivo*, Pyr‐Apelin‐13 treatment prevented PAB‐induced decreases in cardiac output and cardiac index at both 10 days and 10 weeks and preserved RV–pulmonary artery coupling (Ees/Ea). *In vitro*, siRNA apelin‐conditioned media decreased RVEC migration and Matrigel tube formation and decreased RVCM activation of the prosurvival mediator ERK1/2 and the procontractile signaling mediator PKC. Pyr‐Apelin treatment was able to restore, in part, ring formation in SuHx RVECs, and this was blocked by cotreatment with the apelin receptor antagonist, ML221. Apelin and APLNR are decreased in PAH‐RVs, and loss of apelin signaling results in more severe RV failure. *In vitro*, apelin restores angiogenic capacity in RVECs and contractile signaling in RVCMs. Identification of pathways and targets engaged by apelin during RV failure will allow for the development of novel, long‐acting and targeted treatment strategies for RV failure.

## A120 A CRITICAL CONTRIBUTION OF CARDIAC MYOFIBROBLASTS IN RIGHT VENTRICULAR FAILURE AND THE ROLE OF UCP2 SNPS IN THE PREDISPOSITION TO RIGHT VENTRICULAR DECOMPENSATION IN PULMONARY ARTERIAL HYPERTENSION

Yongneng Zhang, Maria Areli Lorenzana‐Carrillo, Saymon Tejay, Yongsheng Liu, Alois Haromy, Gopinath Sutendra, Evangelos D Michelakis

*Department of Medicine (Cardiology), Faculty of Medicine and Dentistry, University of Alberta, Edmonton, Alberta, Canada*

The compensatory stage in right ventricular hypertrophy (RVH) in pulmonary arterial hypertension (PAH) is much shorter than in left ventricular hypertrophy (LVH) in systemic hypertension. Between two patients with the same degree of PAH, one might have a much shorter compensated RVH (cRVH) stage than the other. We hypothesized that right ventricular (RV) cardiac myofibroblasts (myoCF) might contribute to these differences. Their ability to contract (stiffening), produce collagen and promote inflammation contributes to heart failure. Decreased mitochondrial calcium (mCa^2+^) promotes myoCF differentiation (from fibroblasts). Methylation of mCa^2+^ uptake 1 (MICU1) and lack of UCP2 (a component of the mCa^2+^ uniporter complex) activate myoCF in the left ventricle (LV). In a rat PAH model (monocrotaline‐PAH), we measured the RV pressure from isolated perfused hearts and sarcomere shortening from isolated RV cardiomyocytes. We found that the RV pressure was lower in decompensated RVH (dRVH) compared with normal RV, but there was no difference in cardiomyocyte sarcomere shortening between the two groups, pointing to a potential detrimental role of RV myoCF rather than contractile failure of RV cardiomyocytes. Decompensated RVH has much more myoCF compared with normal RV. Cardiac myofibroblasts from dRVH have increased MICU1 methylation and decreased UCP2 levels compared with normal RV fibroblasts. In a cohort (*n* = 25) of patients with pulmonary hypertension that had been treated with the same protocol for years and controlled for age/sex/duration of disease, we found that homozygotes for UCP2 loss‐of‐function single nucleotide polymorphisms (SNPs) had worse RV function compared with heterozygote or wild‐type, despite similar PA pressures. Our preliminary work suggests that RV myoCF rather than RV cardiomyocytes might drive RV decompensation independent of RV afterload in PAH patients, and this might be predicted by UCP2 SNPs that are relatively frequent in the population and have been shown to be associated with PAH outcomes (10‐year survival, time to transplantation).

## A121 INCREASED BONE MORPHOGENETIC PROTEIN 10 ACTIVITY IS ASSOCIATED WITH INCREASED RIGHT ATRIAL WALL STRESS AND DISEASE SEVERITY IN PRECAPILLARY PULMONARY HYPERTENSION

A Llucià‐Valldeperas, J van Wezenbeek, J A Groeneveldt, G Sanchez‐Duffhues, R Smal, X Pan, A Vonk Noordegraaf, H J Bogaard, M J Goumans, F S de Man

*Department of Pulmonary Medicine, Amsterdam Cardiovascular Sciences, Amsterdam UMC, Vrije Universiteit Amsterdam, Amsterdam; Cell and Chemical Biology, Leiden UMC, Leiden, Netherlands*

Pulmonary hypertension (PH) is characterized by increased right atrial (RA) stretch and pressure. The major genetic predisposing risk factor for PH involves mutations in bone morphogenetic protein (BMP) receptor 2 (BMPR2), for which BMP9 and BMP10 are ligands. Although BMP9 is mostly produced by hepatocytes, BMP10 is predominantly produced by adult RA cardiomyocytes. Therefore, its role is of interest in PH. However, the mechanism leading to BMP10 release remains elusive. We analysed BMP10 and BMP9 plasma levels in PH patients using enzyme‐linked immunosorbent assay [*n* = 22 idiopathic pulmonary arterial hypertension (iPAH), *n* = 14 hereditary PAH (hPAH) and *n* = 11 chronic thromboembolic PH (CTEPH)] and *n* = 16 controls. To determine BMP10 and BMP9 activity, we exposed endothelial cells to 10% serum. BMP activity was analyzed as the read out of a BRE‐luciferase reporter assay. Serum was incubated with trap antibodies: anti‐BMP9 to inhibit BMP9 ligand and ALK1‐Fc to inhibit both BMP9 and BMP10 ligands. Consequently, we deducted BMP9 and BMP10 transcriptional activity from the difference versus baseline and BMP9 activity. Plasma BMP10 levels were increased in PH‐patients: 16.5 (9.5–69.7) pg/mL in iPAH, 17.1 (1.4–113.4) pg/mL in hPAH and 37.8 (10.7–87.1) pg/mL in CTEPH, versus 6.1 (0–8.7) pg/mL in controls. We did not detect differences in BMP9 levels. In addition, BMP10 and BMP9 transcriptional activity was unaltered. Next, we divided the PH‐cohort on median BMP10 activity (0.33 a.u.). Higher transcriptional BMP10 activity in PH patients was associated with increased RA volume and pressure and reduced RA compliance (D–F). Furthermore, higher BMP10 transcriptional activity was associated with worse disease status, shown by worse right ventricular function, reduced stroke volume and elevated N‐terminal pro‐brain natriuretic peptide levels (G–I). Although BMP10 circulating levels were increased, transcriptional BMP10 activity was preserved in PH patients. No differences were observed in BMP9 levels or activity. High BMP10 activity was associated with increased RA pressure and worse disease severity in PH patients.

## A122 SPECKLE TRACKING–BASED DEFORMATION ANALYSIS IN PATIENTS WITH PULMONARY HYPERTENSION FROM AN EASTERN EUROPEAN PULMONARY HYPERTENSION CENTRE

Dragos‐Gabriel Iancu, Andreea Varga, Ioan Tilea

*‘G. E. Palade’, University of Medicine, Pharmacy, Sciences and Technology, Targu Mures, Romania*

Owing to the sophisticated structure and geometry of the right ventricle (RV), the assessment of its function is an indicator for tailored therapies and outcomes in pulmonary hypertension (PH) patients. Advanced echocardiographic techniques (two‐dimensional speckle tracking) allow quantification of RV systolic function in PH patients. The prognostic significance of impaired global longitudinal strain of the right ventricle (GLSRV) is unclear in this disease. A cohort of 24 pulmonary arterial hypertension (PAH) and chronic thromboembolic pulmonary hypertension (CTEPH) adults (15 female) was studied. Echocardiography was performed using a Vivid™ E9 XDclear system (General Electric, Healthcare, Boston, MA, USA) with a phased array 3.5 MHz transducer. All patients had been already treated with PH‐specific regimens. We hypothesized that changes in GLSRV values are related to the risk of possible cardiovascular events (CVEs) in a 12‐month follow‐up period. The evaluation of GLSRV compared with the guideline‐related risk of CVEs showed a statistically significant difference, proving that as the value of GLSRV increases, the risk of CVEs decreases (*p* = 0.01). On analysis of the correlation between World Health Organization (WHO) functional class assessed by 6MWT and the GLSRV, no statistically significant difference was found (*p* = 0.83). In our cohort, the GLSRV did not show a statistically significant difference when comparing the basic characteristics with the WHO functional class (*p* = 0.49). Evaluation of GLSRV related to the risk of CVEs demonstrates that an increase in GLSRV value is correlated with a decrease in possible risk of CVEs. A higher GLSRV value is related to a greater walk distance. Recorded GLSRV values did not show a statistically significant difference from the WHO functional class in the basic characteristics of the enrolled patients. Further studies and comparisons with the results of cardiac magnetic resonance imaging are needed for explore the capabilities of this echocardiographic method.

## A123 THE PROGNOSTIC VALUE OF GENETICS ADDED TO THE FRENCH PULMONARY HYPERTENSION REGISTRY RISK SCORE

Alejandro Cruz‐Utrilla, Natalia Gallego, Mauro Lago‐Docampo, Williams Hinojosa, Carmen Pérez Olivares‐Delgado, Jair Tenorio Castaño, Julián Palomino Doza, Fernando Arribas Ynsaurriaga, María Pilar Escribano Subias

*Pulmonary Hypertension Unit, Department of Cardiology, Hospital Universitario 12 de Octubre, Madrid; Instituto de Genética Médica y Molecular (INGEMM), Hospital Universitario La Paz, Madrid; CIBERER, Centro de Investigación en Red de Enfermedades Raras, Instituto de Salud Carlos III, Madrid; ITHACA, European Reference Network on Rare Congenital Malformations and Rare Intellectual Disability Pulmonary Hypertension Unit, Department of Cardiology, Hospital Universitario 12 de Octubre, Madrid; Department of Cardiology, Hospital Universitario 12 de Octubre, Madrid; Centro de Investigación Biomédica en Red de Enfermedades Cardiovasculares (CIBERCV), Madrid, Spain*

Pulmonary arterial hypertension (PAH) is a heterogeneous entity with important genetic background. Risk assessment is essential to evaluate the probability of death or lung transplantation. Nevertheless, current risk scores neglect genetic predisposition, which can reclassify PAH group and risk. Our aim was to create a simplified risk score including genetics. Patients with idiopathic, heritable and toxin‐induced PAH and patients with heritable or sporadic pulmonary veno‐oclusive disease (PVOD) were included from the REHAP registry. Cox regression analysis including baseline variables of the validated French Pulmonary Hypertension Registry (FPHR) score was compared with the same model with addition of the presence of a pathogenic/likely pathogenic variant. Harrel's C statistic compared scores. Three hundred and four adult patients were included from a cohort of 493 patients belonging to the REHAP registry between 2011 and 2021. Median age was 42.5 (34.0–57.0) years, and 70.7% were women. At least one pathogenic/likely pathogenic variant was discovered in each patient in group 1, composed of 20 PVOD patients and 43 heritable PAH cases. Group 2 included 28 sporadic PVOD cases (representing 11.6%), 193 idiopathic or familiar without genetic variants PAH and 20 cases of toxin‐induced PAH. Despite the higher prevalence of PVOD in group 1, diffusing capacity of the lung for carbon monoxide was similar (61.0 vs. 62.0% in the latter; *p* = 0.568). Variant carriers were significantly younger (36.0 vs. 46.0 years in non‐carriers; *p* < 0.001) and had a more severe haemodynamic disease (mean pulmonary artery pressure 55.0 vs 50.0 mmHg in non‐carriers; *p* = 0.029). The presence of a genetic variant was associated with a higher risk of death or lung transplantation (hazard ratio 1.57; 1.01–2.46; *p* = 0.047). Harrel's C statistic was 0.634 after applying the FPHR score. The addition of the genetic testing results increased the goodness of fit of the multivariable model up to 0.666. in conclusion, a simple risk score including genetics strengthens the prognostic value of current multivariable models.

## A124 A PRELIMINARY STUDY TO DEVELOP A MACHINE LEARNING‐BASED PREDICTIVE MODEL FOR PULMONARY EMBOLISM

Linfeng Xi, Min Liu, Rongguo Zhang, Chen Wang

*China–Japan Friendship Hospital, Beijing, China; National Center for Respiratory Medicine, Beijing; Institute of Respiratory Medicine, Chinese Academy of Medical Sciences, Beijing; National Clinical Research Center for Respiratory Diseases, Beijing; Capital Medical University, Beijing; Department of Radiology, China–Japan Friendship Hospital, Beijing; Artificial Intelligence Scholar Center, Infervision, Beijing; Department of Pulmonary and Critical Care Medicine, Center of Respiratory Medicine, China–Japan Friendship Hospital, Beijing; Department of Respiratory Medicine, Capital Medical University, Beijing, China*

Pulmonary embolism (PE) is a life‐threatening disease and is easily misdiagnosed or missed owing to nonspecific clinical symptoms. A simple, accurate and real‐time assessment tool for PE diagnosis is necessary. We aimed to develop a model to predict PE based on machine learning (ML) and validated the efficacy of the ML model established. This was a retrospective study at the China–Japan Friendship Hospital from January 2019 to December 2019. Patients undergoing computed tomography pulmonary angiography (CTPA) for suspected PE were included, and clinical probability for PE was determined according to Wells score, revised Geneva score and Year's algorithm. Additionally, we combined clinical probability with laboratory data to verify whether the fusion model could improve the diagnostic efficacy. We also developed ML models based on different algorithms, including naïve Bayes, logistic regression, *k*‐ nearest neighbors, random forest (RF), decision tree, gradient‐boosting decision tree, support vector machine and multi‐layer perceptron, and the most suitable algorithm was selected. The diagnostic efficacy of the ML model was validated through internal data set. The study included 661 patients with suspected PE, of whom 141 (21.3%) were diagnosed as PE by CTPA. We found that d‐dimer improved the accuracy and negative predictive value (NPV) of the clinical probability, especially the Years algorithm. In addition, the RF algorithm was the most appropriate to build the model. We selected the top eight important features for modeling, including d‐dimer, cTNT, arterial oxygen saturation, heart rate, chest pain, lower limb pain, hemoptysis and chronic heart failure. Finally, the RF model we developed had an area under the curve of 0.813 and NPV of 0.938. In contrast to the traditional clinical probability with d‐dimer or not, the RF model to diagnose PE is superior and has the best overall efficacy, enabling earlier identification of PE.

## A125 LIVER TESTS IN A CLUSTER OF PULMONARY HYPERTENSION PATIENTS: 1‐YEAR FOLLOW‐UP SINGLE‐CENTER OBSERVATIONAL STUDY

Liviu Cristescu, Dragos‐Gabriel Iancu, Andreea Varga, Ioan Tilea

*‘G. E. Palade’, University of Medicine, Pharmacy, Sciences and Technology, Targu Mures, Romania*

Impaired liver function is frequently seen in pulmonary hypertension (PH) patients, and it is related to the progression of the disease itself or to the side‐effects of PH‐targeted therapy. Herein, we investigated the changes of transaminases, bilirubin and lipids in a cohort of PH patients followed up in a single center from Romania. Values of aminotransferases (ALAT and ASAT), total bilirubin (TB), total cholesterol (TC) and triglycerides (TG) from 36 adult PH patients were extracted and analysed from the electronic database of a single PH center from Romania. Patients were divided into two groups: group I, 26 pulmonary arterial hypertension (PAH) patients (nine male, 17 female, mean age 42.03 ± 18.4 years); and group II, 10 chronic thromboembolic pulmonary hypertension (CTEPH) patients (four male, six female, mean age 58.5 ± 9.6 years). Data were analyzed at two different time points: at starting PH‐targeted therapies and after 1‐year follow‐up. The evaluation of aminotransferases at first admission and at 12 months showed there was no statistically significant difference for group I (*P*_ALAT_ = 0.31, *P*_ASAT_ = 0.08), with respective to group II (*P*_ALAT_ = 0.93, *P*_ASAT_ = 0.58). Analysis of the comparison for total bilirubin was nonsignificant for group I (*p* = 0.46) and group II (*p* = 0.61). In our cohort, no significant changes were identified regarding the lipid profile in both groups: group I (*P*_TC_ = 0.72, *P*_TG_ = 0.64) and group II (*P*_TC_ = 0.19, *P*_TG_ = 0.80). No significant differences were identified for the studied parameters between group I and group II when we performed inter‐sample analyses. Although patients with PH are prone to hepatotoxicity owing to a mixture of liver metabolization of the treatment and stasis liver cell injury, we concluded that in a short‐term follow‐up (12 months) there were no significant differences regarding the liver tests in a small sample of PH patients, irrespective of PH‐targeted drug regimen. A multicenter study focused on an increased number of patients stratified on treatment deriving data should be performed in the future.

## A126 MECHANOSENSING IN PULMONARY HYPERTENSION: ROLE OF PIEZO2

Siyu Tian, Esther van de Kamp, Karin Boomars, Daphne Merkus

*Department of Cardiology; Department of Pulmonary Medicine, Erasmus MC, Rotterdam, The Netherlands*

Mechanical forces are translated into biochemical stimuli by mechanotransduction channels, such as the mechanically activated cation channel Piezo2. Lung Piezo2 expression has recently been shown to be restricted to endothelial cells. Hence, we aimed to investigate the role of Piezo2 in regulation of pulmonary vascular function and structure, in addition to its contribution to development of pulmonary vascular disease. Functional studies in human pulmonary microvascular endothelial cells (MVECs) exposed to shear stress (72 h exposure to normal and high shear stress, 21 and 67 dyn/cm^2^, respectively, in an Ibidi system) illustrated that siRNA‐mediated Piezo2 knockdown impaired endothelial alignment, calcium influx (40% lower), phosphorylation of AKT (70% lower) and nitric oxide production (15% lower). In addition, siPiezo2 reduced the expression of the endothelial marker PECAM‐1 (48%) and increased expression of vascular smooth muscle markers ACTA2 and SM22a at both mRNA (1.7‐ and 1.4‐fold) and protein (2.3‐ and 1.7‐fold) levels. In MVECs, Piezo2 expression was reduced in response to abnormal shear stress, hypoxia (30%) and transforming growth factor‐β stimulation (50%). Furthermore, in lung tissue from mice with a *Bmpr2* ^*+/R899X*^ knock‐in mutation commonly found in pulmonary hypertension patients and from a swine model with pulmonary vein banding, the expression of Piezo2 was significantly reduced (26% and 63%, respectively). Thus, Piezo2 acts as a mechanotransduction channel in pulmonary MVECs, stimulating shear‐induced production of nitric oxide, and is essentially involved in preventing endothelial‐to‐mesenchymal transition. Its blunted expression in pulmonary hypertension could impair the vasodilator capacity and stimulate vascular remodelling, indicating that Piezo2 might be an interesting therapeutic target to attenuate progression of the disease.

## A127 TARGETING PTBP1/PKM2‐DRIVEN GLYCOLYSIS IN ENDOTHELIAL CELLS: A NOVEL APPROACH TO TREAT PULMONARY ARTERIAL HYPERTENSION

I Cuthbertson, R Sutcliffe, N W Morrell, P Caruso

*Division of Respiratory Medicine, Department of Medicine, University of Cambridge School of Clinical Medicine, Addenbrooke's Hospital, Cambridge, UK*

Pulmonary arterial hypertension (PAH) is an often‐fatal disease, characterized by the development of apoptosis‐resistant, hyperproliferative and hyperglycolytic endothelial cells (ECs). Loss‐of‐function *BMPR2* mutations and metabolic dysfunction are pivotal to PAH EC dysfunction. Enhanced expression of the glycolysis enzyme pyruvate kinase M2 isoform (PKM2) and upstream splicing factor poly‐pyrimidine tract binding protein (PTBP1) have been observed regardless of genetic background, suggesting that targeting the PTBP1/PKM2 axis could be a potent therapeutic strategy in PAH. Apigenin has been recognized for its low toxicity and ability to suppress PTBP1‐ and PKM2‐driven Warburg glycolysis and tumorigenesis. This work investigates the effects of apigenin on the PKM2/PTBP1 axis in ECs; whether treatment could correct hallmark characteristics of PAH EC dysfunction; and the influence of *BMPR2* mutations. Here, we examined the proteomic, metabolic and functional profiles of blood outgrowth endothelial cells (BOECs) obtained from healthy control volunteers and PAH patients with *BMPR2* mutations, following apigenin treatment or *PTBP1/PKM2* knockdown. We hypothesized that apigenin treatment would reduce glycolysis by inhibiting PTBP1‐driven splicing of *PKM* into *PKM2* and PKM2‐dependent expression of glycolytic pathway components, such as lactate‐dehydrogenase A (LDHA) and lactate production. In control and *BMPR2* mutant BOECs, apigenin inhibited expression of PTBP1 and tetrameric PKM2. In control and *BMPR2* mutant BOECs, apigenin also suppressed LDHA protein expression and lactate production. Apigenin inhibited cell cycle progression and caspase 3/7 activity in control and *BMPR2* mutant BOECs, suggesting that treatment might reduce EC susceptibility to proliferation and apoptosis, hallmarks of PAH ECs. Apigenin treatment might suppress EC glycolysis, possibly through suppression of PKM2‐driven glycolytic gene expression and tetrameric activity. These encouraging data suggest that apigenin might correct the endothelial cell dysfunction observed in PAH, including susceptibility to apoptosis and proliferation.

## A128 HISTONE DEACETYLASE‐DEPENDENT EPIGENETIC MODULATION OF REGULATORY T CELL FUNCTION AND IMMUNE HOMEOSTASIS IN PULMONARY ARTERIAL HYPERTENSION

Chien‐Nien Chen, Fu‐Chiang Yeh, Chong‐Yang Xie, Martin R. Wilkins, Lan Zhao

*National Heart and Lung Institute, Imperial College London, Hammersmith Hospital, Du Cane Road, London, UK*

Immune dysregulation is a common feature of pulmonary arterial hypertension (PAH); however, how altered immune responses are involved in PAH development remains unclear. We report a histone deacetylase (HDAC)‐dependent epigenetic modulation of T‐cell immunity in PAH, which can be targeted pharmacologically. By analysing peripheral blood mononuclear cells (PBMCs) from 41 idiopathic PAH (IPAH) patients, we demonstrate that increased HDAC activity is associated with impairment of CD25^+^Foxp3^+^ regulatory T‐cell (Treg) function and programmed cell death‐1 (PD‐1)‐mediated immune checkpoint signalling in IPAH patients. T cells from patients exhibit a cluster of epigenetic‐sensitive genes involved in the immune vascular regulatory network. An HDAC inhibitor, suberoylanilide hydroxamic acid (SAHA, Vorinostat), differentially modifies these genes and induces Foxp3‐dependent Treg conversion in patient T cells *ex vivo*. Rodent models recapitulate these epigenetic aberrations and T‐cell dysfunction. In three rodent models [monocrotaline (MCT) rats, Sugen5416–hypoxia (SuHx) rats and mice], SAHA treatment restores the Treg population and attenuates elevated pulmonary arterial pressure, right ventricular hypertrophy and pulmonary vascular remodelling. SAHA also reduces extracellular matrix enzymatic activities, upregulates BMPR2 and downregulates PD‐L1 in PAH lungs. Chromatin immunoprecipitation analysis reveals enriched acetylated histone H3 and H4 bound to Foxp3 promoter in PMBCs from SAHA‐treated rats. Noticeably, selective depletion of Tregs with anti‐CD25 monoclonal antibody significantly limits the therapeutic effects of SAHA in SuHx mice. Furthermore, SAHA inhibits tumor necrosis factor‐α induced cytokine/chemokine release from human pulmonary artery endothelial cells and subsequent immune cell chemotaxis in vitro, simulating the scenario of impeded recruitment of immune cells to pulmonary vasculature in vivo. This study reveals a pivotal role of HDAC‐dependent epigenetic regulation in circulating immune cells, providing fresh impetus for the clinical evaluation of HDAC inhibition as a therapeutic strategy in PAH and other immune–pulmonary diseases.

## A129 CHARACTERIZATION OF THE PULMONARY VASCULAR EFFECTS OF THE NOVEL K_V_7 CHANNEL ACTIVATOR URO‐K10

Marta Villegas Esguevillas, Suhan Cho, Alba Vera‐Zambrano, Jae Won Kwon, Bianca Barreira, Laura Moreno, Francisco Pérez Vizcaino, Sung Joon Kim, Belén Climent, Ángel Cogolludo

*Department of Pharmacology and Toxicology, School of Medicine, Complutense University of Madrid, Madrid, Spain; Department of Physiology, Department of Biomedical Sciences, Seoul National University College of Medicine, Seoul, Korea; Institute of Health Research Gregorio Marañón (IiSGM), Madrid; Ciber Respiratory Diseases (CIBERES), Spain; Department of Physiology, School of Pharmacy, University Complutense of Madrid, Madrid, Spain*

Pulmonary arterial hypertension (PAH) is a rare disease characterized by exaggerated pulmonary vasoconstriction and proliferation of pulmonary artery smooth muscle cells (PASMCs). Voltage‐dependent K^+^ (K_V_) channels are responsible for regulating PASMC membrane potential, which, in turn, controls the opening of Ca^2+^ channels, whose cytosolic concentration regulates contraction, proliferation and hypertrophy of these cells. Pulmonary arterial hypertension is associated with ionic remodelling characterized by, among other things, downregulation of K_V_1.5 and TASK‐1 channels. Recent studies have shown that K_V_7 channels are preserved in animal models of this disease. Hence, K_V_7 channel agonists might represent an attractive strategy for PAH treatment. In this study, we have analysed, for the first time, the effects of URO‐K10, a novel K_V_7 channel modulator, on the pulmonary vasculature. Our data show that URO‐K10 exerts a more potent vasodilatory effect on pulmonary arteries (PAs), via K_V_7 channel activation, than the classical K_V_7 activator retigabine, and increases K_V_ current in PASMCs from control rats. In addition, URO‐K10 causes a concentration‐ dependent antiproliferative effect on human PASMCs. It is noteworthy that the pulmonary vasodilator efficacy of this compound was increased considerably in conditions mimicking ionic remodelling (as an in vitro model of PAH) and in arteries from monocrotaline‐induced PAH rats. Likewise, URO‐K10 increases the K_V_ current density in isolated PASMCs from the in vitro model and the in vivo model of PAH. Interestingly, the K_V_7 channel‐sensitive current is activated when the cell is depolarized, and importantly, this compound is able to repolarize the cell membrane potential of isolated PASMCs from the in vitro model of PAH. Taken together, our results confirm the interest of K_V_7 channels as a potential target in PAH and highlight that URO‐K10, owing to its higher potency and efficacy, might represent a better alternative than classical K_V_7 activators.

## A130 CASE REPORT: FOLLOW‐UP CHRONIC THROMBOEMBOLIC PULMONARY HYPERTENSION COMPUTED TOMOGRAPHY THREE‐DIMENSIONAL‐DIMENSIONAL RECONSTRUCTION—REMOTE METHODOLOGY

Juaneda Ernesto, Massano Gabriel

*3D Biomodel Unit, Children's Hospital, Córdoba, Argentina*

The patient was 67‐year‐old man with metabolic syndrome and recent dyspnea funtional class III, who had a previous episode of thromboembolic pulmonary hypertension and discontinuation of antigoagulation treatment after clinical improvement. On screening, two‐dimensional color Doppler echocardiography depicted pulmonary hypertension, and laboratory tests demonstrated increased d‐dimer and homocysteine. High‐resolution contrast computed tomography (CT) showed chronic thromboembolic pulmonary hypertension (CTEPH). Medical treatment with a new oral anticoagulant and folic acid vitamin B were indicated. The CT DICOM file on digital report allowed three‐dimensional (3D) remote reconstruction and display through a link sent to the user's smartphone: external, internal (navigation), 360° rotation and magnification of the pulmonary artery (PA). Three‐dimensional reconstruction before and after 12 months of conservative treatment demonstrated qualitative pulmonary artery thrombus reduction to functional class I. 3D CT in CTEPH remote methodology through adquisition, post‐processing and display might be a useful tool in follow‐up of CTEPH.

## A131 PRELIMINARY STUDY ON ESTABLISHMENT OF RAT MODEL OF CHRONIC THROMBOEMBOLIC PULMONARY HYPERTENSION

Wenyi Pang, Lianhua Liu, Jixiang Liu, Risheng Hao, Zhenghao Zhang, Zhenguo Zhai

*Department of Pulmonary and Critical Care Medicine, Center of Respiratory Medicine, China–Japan Friendship Hospital, Beijing; National Clinical Research Center for Respiratory Diseases, Beijing; Department of Pulmonary and Critical Care Medicine, Beijing Jishuitan Hospital, Beijing, P.R. China*

The pathophysiological mechanism of chronic thromboembolic pulmonary arterial hypertension (CTEPH) has not been elucidated fully, and the treatment of CTEPH still faces many challenges. However, owing to the lack of mature animal models, translational medicine research progresses slowly. The purpose of this study was to explore a combination of the inhibitor of vascular endothelial growth factor (VEGF) receptor tyrosine kinase (Sugen5416) and multiple autologous thrombus injections to establish a rat animal model, and to provide ideas for establishing small animal models of CTEPH. Male Sprague–Dawley rats were randomly divided into five groups of three rats each: control group (Ctrl; injected with an equal volume of normal saline) and 6, 12, 24 and 48 h injection groups. Blood was collected from the orbital vein to prepare autologous thrombi, which were injected into the jugular vein. All rats were killed at corresponding time points. Lung tissue was harvested for Hematoxylin and Eosin staining to observe the dissolution of autologous thrombi. Male Sprague–Dawley rats were randomly divided into four groups of 8–12 rats each: control (Ctrl; injected with an equal volume of saline); autologous thrombi injection (PE); Sugen5416 administration (SU); and Sugen5416 combined with autologous thrombi injection (PE + SU). The SU and PE + SU groups each received a single dose of Sugen5416 (20 mg/kg) intraperitoneally 1 day before the first injection of thrombi. The PE and PE + SU groups each received three autologous thrombi injections through the left jugular vein on experimental days 1, 3 and 5. At the same time, tranexamic acid (315 mg/kg/day) was injected intraperitoneally every day during the first 2 weeks of modeling. At the end of the fifth week, pulmonary artery pressure, percentage media thickness (MT%) and right ventricular hypertrophy index (RVHI) were measured in each group to evaluate the animal model. Thrombi were dissolving in the rat main pulmonary artery and lobe pulmonary artery at 12 h after the first injection of thrombi; and thrombi gradually dissolved to 50% of the initial injected thrombi volume after 24 h; and autologous thrombi were almost completely dissolved after 48 h. Mean pulmonary arterial pressure was elevated in the PE + SU group compared with the control, PE and SU groups (27.96 ± 4.53 vs. 14.59 ± 2.51, 17.67 ± 2.99 and 19.78 ± 1.68 mmHg, *p* < 0.0001 or *p* < 0.001). The MT% was increased in the PE + SU group compared with the control, PE and SU groups (49.04 ± 10.37 vs. 27.12 ± 5.13, 33.52 ± 6.16 and 37.17 ± 6.90%, all *p* < 0.0001). The smooth muscle layer was markedly thickened in the PE + SU group. The RVHI in the PE + SU group was higher than that in the control, PE and SU groups (0.42 ± 0.06 vs. 0.26 ± 0.03, 0.29 ± 0.03 and 0.34 ± 0.02, *p* < 0.0001 or *p* < 0.001 or *p* < 0.01). We found that the interaction of Sugen5416 and pulmonary arterial mechanical obstruction by multiple autologous thrombi could establish a mild to moderate CTEPH rat model. This new model also suggests the potential connection between the occurrence and development of CTEPH and endothelial injury and thromboembolism.

## A132 BLOOD DNA METHYLATION PROFILING IDENTIFIES CATHEPSIN Z DYSREGULATION IN PULMONARY ARTERIAL HYPERTENSION

Yukyee Wu, Anna Ulrich, Harmen Draisma, John Wharton, Emilia M Swietlik, Zhanna Balkhiyarova, Marjo‐Riitta Jarvelin, Juha Auvinen, Karl‐Heinz Herzig, J Gerry Coghlan, James Lordan, Colin Church, Luke S Howard, Joanna Pepke‐Zaba, Mark Toshner, Stephen J Wort, David G Kiely, Robin Condliffe, Allan Lawrie, Stefan Gräf, Nicholas W Morrell, Martin R Wilkins, Inga Prokopenko, Christopher J Rhodes

*National Heart and Lung Institute, Imperial College London, London; Department of Clinical and Experimental Medicine, University of Surrey, Guildford; Department of Metabolism, Digestion and Reproduction, Imperial College London, London; Department of Medicine, University of Cambridge, Cambridge, UK; Institute of Biochemistry and Genetics, Ufa Federal Research Centre Russian Academy of Sciences, Ufa, Russia; School of Public Health, Imperial College London, London, UK; Center for Life Course Health Research, University of Oulu, Oulu; Institute of Biomedicine, Medical Research Center Oulu, Oulu University, and Oulu University Hospital, Finland; Department of Pediatric Gastroenterology and Metabolic Diseases, University of Medical Sciences, Poznan, Poland; University College London, London; University of Newcastle, Newcastle upon Tyne; University of Glasgow, Glasgow, UK; Royal Papworth Hospital, Cambridge; National PH Service, Royal Brompton Hospital, London; Department of Infection, Immunity & Cardiovascular Disease, University of Sheffield, Sheffield; Royal Hallamshire Hospital, Sheffield; NIHR BioResource for Translational Research, Cambridge Biomedical Campus, Cambridge, UK; UMR 8199 – EGID, Institut Pasteur de Lille, CNRS, University of Lille, F‐59000 Lille, France; Institute of Biochemistry and Genetics, Ufa Federal Research Centre Russian Academy of Sciences, Ufa, Russia*

Pulmonary arterial hypertension (PAH) is characterized by pulmonary vasculature remodelling, leading to premature death from right heart failure. Rare and common PAH DNA variants have been established. We aimed to explore the role of epigenetic variation in PAH. We measured DNA‐methylation (DNAme) levels at 865,848 CpG sites in peripheral blood samples from 429 individuals with PAH and 1,226 controls using the Illumina EPIC array and tested for association with PAH in multivariable regression models. Three DNAme markers, cg04917472, cg27396197 and cg03144189, that are in close proximity to cathepsin Z (*CTSZ*), conserved oligomeric Golgi complex 6 (*COG6*) and zinc finger protein 678 (*ZNF678*), respectively, showed elevated methylation levels, reaching epigenome‐wide significance (*P* < 10^−7^) in PAH samples versus controls. We observed stable hypermethylation in individuals with PAH at 1‐year follow‐up. Likewise, among established PAH risk‐related genes, a DNAme marker, cg10976975 in *BMP10*, also showed hypermethylation in PAH. Hypermethylation at *CTSZ* was associated with decreased blood cathepsin Z mRNA levels, and CTSZ protein levels in PAH plasma samples were elevated compared with disease‐free controls in matched samples from the same PAH cohort. Knockdown of CTSZ expression in cultured human pulmonary artery endothelial cells using siRNA was associated with a marked increase (*P* < 10^−4^) in caspase‐3/7 activity, including response to two inflammatory triggers, lipopolysaccharide and tumour necrosis factor alpha. PAH is associated with altered blood DNA methylation profiles, exemplified by the protease cathepsin, *CTSZ*, which modifies pulmonary endothelial function. Dysregulation in circulating cathepsin Z could represent a novel druggable pathway.

## A133 INVESTIGATION OF TRANSLOCATOR PROTEIN (TSPO) AS A THERAPEUTIC TARGET IN PULMONARY HYPERTENSION

Farah Sabrin, Ali Ashek, Chien Nien Chen, Lin Zhao, Olivier Dubois, David Owen, Martin Wilkins, Lan Zhao

*National Heart and Lung Institute, Imperial College London, UK*

The mitochondrial translocator protein (TSPO), a conserved mitochondrial outer membrane protein, is highly expressed in both inflammatory and endothelial cells. We investigated the role of TSPO using in vivo rodent models of pulmonary hypertension (PH). Positron emission tomography (PET) imaging with an established TSPO radioligand ([^11^C]PBR28) was performed in monocrotaline (MCT, 60 mg/kg) rats. Significantly increased PBR28 uptake was demonstrated in the MCT PH rat lungs in comparison to healthy controls, with a twofold increase of the rate constant *k*_3_, which represented increased receptor binding. Pretreatment with TSPO ligand XBD‐173 blocked PBR28 signals, confirming the radioligand specificity. This result is consistent with the hyperproliferative pulmonary vascular pathology and inflammatory cell infiltration in the MCT rat lungs. Pharmacological efficacy of TSPO ligand XBD‐173 (2 mg/kg, twice daily) was assessed using both MCT and Sugen–hypoxia (SuHx) rat models. In both models, XBD‐173 treatment attenuated the increased pulmonary arterial pressure (MCT, 27.23 ± 5.2 vs. 45.37 ± 7.6 mmHg, *P* ˂ 0.001; and SuHx, 50.96 ± 8.0 vs. 63.03 ± 4.02 mmHg, *P* ˂ 0.01), right ventricular hypertrophy and pulmonary vascular remodelling (percentage of muscularized peripheral vessels; MCT, 53.57 ± 3.5 vs. 75.24 ± 6.3%, *P* ˂ 0.05; and SuHx, 56.09 ± 8.14 vs. 80.02 ± 11.25%, *P* ˂ 0.05). XBD‐173 treatment also decreased perivascular CD68^+^ macrophage infiltration in MCT rats, and it alleviated peripheral small pulmonary vessel occlusion in SuHx rats. PET imaging with [^18^F]‐FDG showed that XBD‐173 treatment significantly attenuated lung glucose uptake. The results of the present study provide the basis for further clinical investigation of TSPO as a potential biomarker and therapeutic target in PH patients.

## A134 [^11^C]‐HYDROXYEPHEDRINE PET EVALUATION OF SYMPATHETIC FUNCTION IN EXPERIMENTAL AND HUMAN PULMONARY HYPERTENSION

Jason Zelt, Virgilio Cadete, Yupu Deng, Rosemary Dunne, Robert deKemp, Rob Beanlands, George Chandy, Vladimir Contreras‐Dominguez, Duncan Stewart, Lisa Mielniczuk

*Department of Cellular and Molecular Medicine and Department of Medicine, Faculty of Medicine, University of Ottawa, Ottawa; Sinclair Centre for Regenerative Medicine, Ottawa Hospital Research Institute, Ottawa; Division of Cardiology, University of Ottawa Heart Institute and University of Ottawa, Ottawa, ON; Molecular Function and Imaging Program, The National Cardiac PET Centre, and Advanced Heart Disease Program, Division of Cardiology, Department of Medicine, University of Ottawa Heart Institute, Ottawa, ON; Division of Respirology and Internal Medicine, University of Ottawa Heart Institute and University of Ottawa, Ottawa, ON, Canada*

Little is known about the sequelae of sympathetic nervous system (SNS) activation in patients with pulmonary hypertension (PH). This study assessed the feasibility of using cardiac posiron emission tomography (PET) to evaluate the SNS in animal models and humans with PH. Experimental PH was induced in Sprague–Dawley rats (*n* = 9) using the Sugen + chronic hypoxia (SUHx) model. Patients with chronic right heart failure (RHF) secondary to PH (group I *n* = 2, group II *n* = 8 and group III *n* = 1) were recruited. Healthy cohorts of rats (*n* = 7) and humans (*n* = 7) were also included. Myocardial SNS function was assessed by [^11^C]‐*meta‐*hydroxyephedrine (HED) retention index (RI) using PET. In rats, SUHx induced severe PH with elevated right ventricular (RV) pressures compared with controls (103.9 ± 6.5 vs. 28.2 ± 0.1 mmHg, *p* < 0.0001). Regional sympathetic dysfunction was observed in SUHx rats compared with controls in the RV [RV/left ventricle (LV) ratio: 0.46 ± 0.12 versus 0.71 ± 0.08, respectively; *p* < 0.001] and septum (septum/lateral wall (LW) ratio: 0.60 ± 0.19 vs. 1.03 ± 0.05, respectively; *p* < 0.0001). The degree of sympathetic dysfunction was correlated positively with RV stroke volume (RV: *r* = 0.67, *p* = 0.05; septum: *r* = 0.82, *p* = 0.007). Humans with PH did not display any obvious sympathetic dysfunction or any significant differences in the RV/LV ratio (*p* = 0.21) or septum/LW ratio (*p* = 0.10). However, there was marked heterogeneity in HED RI in PH compared with controls. In humans, the extent of sympathetic dysfunction was correlated negatively with N‐terminal pro‐brain natriuretic peptide levels (RV/LV ratio: *r* = −0.79, *p* = 0.004; septum/LW ratio: *r* = −0.58, *p* = 0.05). These data provide mechanistic insight into the state of SNS dysfunction within the failing RV in experimental and clinical PH. In both rats and patients with PH, the extent of sympathetic dysfunction in the RV and septum was directly correlated with markers of RV dysfunction. This suggests that HED can quantify the burden of SNS abnormalities in both animals and humans, with the potential to monitor the impact of neurohormonal therapies non‐invasively for patients with RHF.

## A135 CONTRAST‐FREE PULMONARY ANGIOGRAPHY IN PULMONARY ARTERIAL HYPERTENSION

Samar Farha, Jason Lempel, Trishul Siddharthan, Jacqueline Sharp, Brittany Beck, Kewal Asosingh, Suzy A Comhair, Jason Kirkness, Andreas Fouras, Serpil C Erzurum

*Cleveland Clinic, Cleveland, OH; University of Miami, Miami, FL; 4DMedical, Los Angeles, CA, USA*

Pulmonary arterial hypertension (PAH) is characterized by pulmonary vascular remodeling. Existing segmentation software programs require use of contrast scan imaging. Contrast‐free pulmonary angiography (CFPA) is a novel image analysis technology that quantifies the pulmonary vasculature using data from non‐contrast scans. We performed a study using CFPA to describe the pulmonary vasculature in PAH compared with controls and correlate measurements with clinical biomarkers. The CFPA image processing uses a novel iterative methodology layered upon established techniques for multi‐scale shape filtering from chest CT, to create a quantitative three‐dimensional (3D) map of the lung vascular tree. Contrast‐free pulmonary angiography provides quantitative data on vessels with diameter ranging from 2 to 15 mm. Diameter measures are sampled every 0.1 mm along each vessel. Segments, vessel diameters, length, area, blood volume and tortuosity are obtained. Four patients with group 1 PAH (newly diagnosed within 4 months) and five controls were enrolled. Hemodynamics, right ventricular systolic pressure (RVSP), 6‐min walk distance and N‐terminal pro‐brain natriuretic peptide were obtained. There was no difference in age and gender between PAH and controls. When comparing 3D imaging, there was sparsity of vessels noted in PAH compared with controls. This was in parallel to ~14% reduction in total segments [segments: PAH 2,385 ± 1,009, controls 2,780 ± 1,454; *p* = 0.3]. Mean and maximum vessel diameters were similar; however, minimum diameter tended to be higher in PAH [minimum diameter (in millimeters): PAH 2.62 ± 0.19, controls 2.32 ± 0.33; *p* = 0.07]. Differences in blood volume, total area, tortuosity and vessel length were not significant between PAH and controls. In PAH, RVSP was inversely correlated with total segments (*p* = 0.02), vessel length (*p* = 0.05), total area (*p* = 0.01), blood volume (*p* = 0.003) and tortuosity (*p* = 0.02). Contrast‐free pulmonary angiography provides quantitative measurements of vessels and blood volumes in newly diagnosed PAH that are correlated with RVSP and have some differences in PAH compared with controls, suggesting that the technology might be useful in the assessment of pulmonary vascular diseases.

## A136 ACUTE β‐BLOCKADE IN PULMONARY ARTERIAL HYPERTENSION IMPROVES PULMONARY HAEMODYNAMICS MEDIATED BY INCREASE IN DIASTOLIC FILLING: AN EXPERIMENTAL CARDIAC MAGNETIC RESONANCE IMAGING AND CLINICAL PRESSURE–VOLUME STUDY

Chinthaka B Samaranayake, Marili Niglas, Aleksander Kempny, Nicoleta Baxan, Ali Ashek, Joy A Pinguel, Laura C Price, Michael Gatzoulis, Konstantinos Dimopoulos, Stephen J Wort, Lan Zhao, Colm McCabe

*National Pulmonary Hypertension Service, Royal Brompton Hospital, London; National Heart and Lung Institute, Imperial College, London, UK*

Despite ongoing controversy over the role of β‐blockers in the treatment of pulmonary arterial hypertension (PAH), little is known about their acute haemodynamic effects on right ventricular (RV) function in this population. The aim was to assess the response of the RV to acute β‐blockade in PAH. In an experimental study, the effects of fixed‐dose esmolol infusion were evaluated in a monocrotaline (MCT) PAH rodent model at 2 weeks (*n* = 8) to measure changes in RV volumes by cardiac magnetic resonance imaging (CMR) compared with controls (*n* = 4). In a clinical study, patients with idiopathic PAH (*n* = 8) were prospectively recruited and underwent RV pressure–volume (PV) catheterization. Haemodynamic and PV data were collected at four study stages: supine rest, supine exercise, supine exercise with intravenous esmolol at 50 μg/kg/min and supine exercise with intravenous esmolol at 100 μg/kg/min at matched workloads (25 W for 3 min), with adequate time for recovery between stages. End‐expiratory PV loops were recorded and analysed at each stage. In the MCT model, esmolol reduced heart rate, which increased RV end‐diastolic volume (EDV) and end‐systolic volume in comparison to controls (*p* < 0.01). The RV stroke volume was maintained in both groups, but LVEF reduced in the MCT model (*p* = 0.018). In patients with idiopathic PAH, supine exercise increased heart rate (*p* = 0.003) and cardiac output (*p* = 0.02), but RV stroke volume reduced owing to impaired diastolic filling (*p* = 0.01). At matched workloads (25 W), esmolol attenuated the increase in exercise heart rate, reversed the exercise‐associated reduction in EDV and increase in end‐diastolic pressure and improved RV diastolic function (Tau and d*P*/d*T*_min_) in a dose–response relationship. Furthermore, esmolol improved the RV stroke work‐to‐cardiac output ratio without affecting systemic vascular resistance. In PAH, esmolol increases RV diastolic filling at rest and during low‐grade exercise. The reduction in LV and RV ejection fraction with esmolol is offset by preserved stroke volume responses and favourable effects on RV stroke work.

## A137 UNDERSTANDING THE CONTRIBUTION OF RARE AQP1 VARIANTS IN THE PATHOPHYSIOLOGY OF PULMONARY ARTERIAL HYPERTENSION

Jennifer Wood, Philip Kitchen, Stephen Moore, Wei Li, Nick Morrell

*Department of Medicine, The University of Cambridge, Cambridge; School of Biosciences, Aston University, Birmingham, UK*

Pulmonary arterial hypertension (PAH) is a rare but devastating disease, for which there is no cure. The identification of rare genetic variation in genes encoding components of the bone morphogenetic protein pathway in patients with heritable PAH has yielded novel targets for therapy. However, more recent genetic studies by our group have identified causal mutations in other genes, including aquaporin 1 (*AQP1*), that provide new insights into disease mechanisms and potential disease‐modifying therapeutic avenues. Of the *AQP1* mutations reported, one mutation, R195W, is recurrent, affecting five of the nine PAH patients with *AQP1* rare variants. This study aimed to elucidate whether mutant AQP1 proteins reach the cell surface, the effect of the AQP1 mutations on water channel permeability and the functional effects on pulmonary vascular cells. Owing to the recurrent nature of the R195W mutation, an *Aqp1* R195W knock‐in mouse was generated to investigate the impact on phenotype in vivo and *ex vivo*, including cardiac catheterization, full blood counts, lung histology, protein/RNA expression in lung and heart tissue, erythrocyte osmotic fragility assays and urine osmolarity. Using transiently transfected Hek293T cells, confocal microscopy and biotin– streptavidin enzyme‐linked immunosorbent assays revealed that the R195W AQP1 mutant proteins reach the plasma membrane at comparable efficiency to wild‐type AQP1. Stably transfected MDCK cells that express the mutant AQP1 proteins are currently being used in calcein self‐quenching assays to compare AQP1 water permeability rates. We have established a lentiviral transient overexpression system to compare the effect of each mutant on pulmonary arterial endothelial cells. Finally, a 6‐month study and a 4‐month study including 3 weeks of chronic hypoxia exposure in the *Aqp1* R195W knock‐in mouse model are ongoing. These studies are revealing the R195W mutation to be loss of function in terms of water channel activity, and further phenotypic characterization of mutant mice is being undertaken.

## A138 HYPERPOLARIZED ^129^XENON MRI GAS EXCHANGE RESEARCH IN PULMONARY VASCULAR DISEASE AND INTERSTITIAL LUNG DISEASE: THE XCHANGE CLINICAL STUDY

Laura Sutton, Jeff Kammerman, Stephanie Cahill, Alex Dusek

*Polarean, Inc., Durham, NC, USA*

Assessing small airway functional impairment, therapeutic response and disease progression in patients with pulmonary vascular and/or interstitial lung disease (ILD) continues to be challenging. Hyperpolarized Xenon (Xe) MRI enables noninvasive evaluation and regional quantification of alveolar gas exchange via three‐dimensional mapping of ventilation, interstitial membrane uptake and red blood cell transfer. This global, open‐label, multicenter study will implement harmonized methodology to evaluate safety and tolerability of hyperpolarized Xe gas in patients with pulmonary hypertension and ILD. Adult patients (*n* = 200) with pulmonary arterial hypertension, chronic thromboembolic pulmonary hypertension, idiopathic pulmonary fibrosis or ILD‐associated systemic sclerosis (SSc‐ILD) will be enrolled and undergo Xe MRI for evaluation of treatment response and comparison with standard‐of‐care diagnostic measurements. Eligible patients will be initiating/changing therapy or undergoing a disease‐specific procedure. An exploratory cohort (*n* = 50) will allow Xe MRI in patients with long‐COVID, unexplained dyspnea, possible transplant (lung or stem cell) rejection, World Health Organization group 3 pulmonary hypertension or other ILDs. Healthy volunteers (*n* = 50) will be enrolled to evaluate Xe MRI reproducibility. Changes in Xe MRI (within patient groups) at 1, 3, 6 and 12 months; Cross‐sectional and longitudinal relationships of Xe MRI with spirometry, including diffusing capacity of carbon monoxide (DLCO); Xe MRI measurements compared with right heart catheterization (RHC) and echocardiogram [PH patients] and computed tomography (CT) [ILD patients]. Relationship of Xe MRI quantitative measures across compartments (air/tissue/blood) to treatment response; Changes in Xe MRI parameters to assess temporal relationship to changes in standard diagnostics, healthcare utilization, quality‐of‐life and morbidity/mortality; Relationship of Xe MRI cardiogenic oscillation signals of pulmonary‐vascular hemodynamics with RHC and clinical outcomes. Patients will begin enrollment within the USA in mid‐2022. All patients will participate for at least 1 year (and up to 3 years). Interim analyses will be conducted yearly, with additional snapshots periodically.

## A139 IS PULMONARY ARTERIAL HYPERTENSION ASSOCIATED WITH THE LOSS OF VESSELS OR VESSEL REMODELING? LESSON FROM SUGEN + HYPOXA RAT MODEL

Baktybek Kojonazarov, Sailun Wang, Anna M Rotert, Stefan Hadzic, Regina Mukhametshina, Khodr Tello, Norbert Weissmann, Friedrich Grimminger, Rajkumar Savai, Soni S Pullamsetti, Peter Dorfmueller, Werner Seeger, Ralph T Schermuly

*Institute for Lung Health (ILH), Justus Liebig University, Giessen, Germany, Department of Internal Medicine, Member of the German Center for Lung Research (DZL), Member of the Excellence Cluster Cardio‐Pulmonary Institute (CPI), Justus Liebig University, Giessen, Germany, Max‐Planck Institute for Heart and Lung Research, Bad Nauheim, Germany*

The contribution of microvascular density in the pathobiology of pulmonary arterial hypertension (PAH) remains incompletely understood. In the present study, we have investigated lung and heart perfusion by in vivo and *ex vivo* approaches to assess whether PAH is associated with loss of vessels or vessel remodelling. We injected rats (Wistar–Kyoto strain; Janvier Labs) subcutaneously with SU5416 (20 mg/kg body weight; Tocris), followed by chronic exposure to hypoxia (10% oxygen) for 21 days (SuHx 3 weeks control) or hypoxia for 21 days and normoxia re‐exposure for an additional 14 days. Rats injected with saline were used as healthy controls. Echocardiography, contrast‐enhanced in vivo and *ex vivo* microscopic computed tomography (µCT), right heart catheterization, three‐dimensional lung stereological assessment of the alveolar density, lung and heart histology were performed. Heart segmentation with quantitative analysis was performed by using a U‐net convolutional neural network. The combination of SU5416 treatment and chronic hypoxia exposure resulted in an increase in right ventricular (RV) systolic pressure, RV mass and lung emphysema. Our data suggested that lung emphysema is progressing in SuHx rats from week 3 to 5 after SU5416 injection and hypoxic exposure, confirmed by µCT and alveolar stereology. *In vivo* contrast µCT imaging revealed that the total RV myocardial blood volume was significantly higher, but RV blood volume normalized to RV myocardial volume decreased in SuHx rats in comparison to the normoxic control. *In vivo* and *ex vivo* µCT imaging of the lung revealed defective lung perfusion. However, histological assessment of the RV and lung vasculature showed no difference in the number of vessels between normoxic and SuHx animals. Our data revealed that PAH SuHx rats had impaired RV function and lung vessel remodeling, but not loss of vessels.

## A140 SOX17 AND RUNX1 MODULATE THE DEVELOPMENT OF PULMONARY ARTERIAL HYPERTENSION

O Liang, E Jeong, M Pereira, E So, M Del Tatto, S Wen, M Dooner, C E Ventetuolo, P J Quesenberry, L Zhao, M R Wilkins, J R Klinger

*Hematology/Oncology Division; Pulmonary, Sleep and Critical Care Medicine Division; Hematology/Oncology Division, Rhode Island Hospital/Brown University, Providence, RI, USA; Imperial College London, London, UK*

Previously, we showed that Runx1 expression is increased in circulating CD34^+^CD133^+^ progenitor cells in patients with pulmonary hypertension (PH) and that inhibition of RUNX1 blocks the egress of bone marrow (BM)‐derived c‐kit^+^ progenitor cells in mice and prevents and reverses established Sugen–hypoxia (Su/Hx)‐induced PH in rats. Recently, loss‐of‐function mutations in the *Sox17* gene were found to be associated with pulmonary arterial hypertension (PAH). RUNX1 is an important downstream target of SOX17. In the present study, we examined the role of Sox17 and Runx1 expression in the pathogenesis of PH. We generated conditional Cdh5‐CreERT2; Runx1(flox/flox) and LysM‐Cre; Runx1 (flox/flox) mice to delete *Runx1* in adult endothelium and myeloid lineage cells, respectively, and exposed them to SuHx. We also measured PH and expression of Sox17 and Runx1 in whole bone marrow (WBM) from wild‐type (WT) and Sox17 enhancer knockout (eKO) mice, constructed to resemble the mutations in PAH patients (Dr Lan Zhao, Imperial College London, UK). Tissue‐specific deletion of *Runx1* in adult endothelium or in cells of myeloid lineage prevented the development SuHx‐PH, suggesting that RUNX1 is required for the development of PH. Sox17 expression in BM was decreased in Sox17 eKO mice compared with WT mice and decreased in both WT and Sox17 eKO mice after exposure to SuHx‐PH. Both exposure to SuHx and disruption of the Sox17 enhancer tended to increase Runx1 expression in WBMs, but the difference was not statistically significant. Although the severity of PH did not differ between Sox17 eKO and WT mice, Sox17 eKO mice developed severe PH at a lower dose of Sugen and hypoxia than WT mice. These results suggest that RUNX1 plays an important role in the pathogenesis of PH and that impaired SOX17 expression might enhance susceptibility to PH by failing to suppress RUNX1 expression.

## A141 ASSOCIATION OF RIGHT VENTRICULAR DIASTOLIC STIFFNESS AND CARDIAC MAGNETIC RESONANCE VARIABLES WITH MORTALITY IN PATIENTS WITH PULMONARY HYPERTENSION

Alexandra Janowski, Charles Fauvel, Scott Visovatti, Raymond L Benza, Rebecca R Vanderpool

*Division of Cardiovascular Medicine, The Ohio State University, Columbus, OH, USA*

Right ventricular (RV) failure is the major cause of mortality in pulmonary hypertension (PH). Diastolic stiffness is significantly associated with outcomes in patients with PH. Cardiac magnetic resonance (CMR) imaging provides an opportunity to image the right ventricle and quantify right heart remodeling. The Ohio State University (OSU) CMR registry includes images, imaging data (ventricular volumes) and vital status. The aim of this study was to investigate the association of right ventricular diastolic stiffness and function with mortality in patients with PH. Patients were identified from the OSU CMR registry by searching the CMR summary statements for keyword close variants of ‘pulmonary hypertension’. Right heart catheterization (RHC) data were collected from retrospective chart review. The RV diastolic stiffness (β) was calculated using the relationship, *P* = α(e^β*V*^ − 1), a fitting constant (α), right atrial pressure, end‐systolic (ESV) and end‐diastolic (EDV) volumes. Arterial afterload (Ea) was the ratio of end‐systolic pressure to stroke volume. Pulmonary hypertension was defined as a mean pulmonary artery pressure ≥ 20 mmHg. The putcome was defined as death, with the index date as the CMR date. Data were presented as the mean ± SD, median [interquartile range] or number (percentage) when appropriate. Univariable Cox models were used to test the association of RV function with outcomes. In participants with complete CMR and RHC data (*n* = 111; age 55 ± 15 years; body surface area 1.96 ± 0.28 m^2^; 62% female), there was a median follow‐up time of 3.23 [1.03–6.06] years (41 events). Within patients with PH (86.5%), 60 had a wedge pressure ≤ 15 mmHg. End‐diastolic elastance (Eed) was increased in patients with PH and highest in patients with an elevated wedge compared with no PH. In the PH group with a wedge pressure ≤ 15 mmHg, Eed, RVEF and SV/ESV were associated with morality. The RV diastolic stiffness is increased in PH and is significantly associated with mortality in patients with pulmonary hypertension.

## A142 AUTOMATED CARDIAC SEGMENTATION AND MOTION ANALYSIS IN RAT MODELS OF PULMONARY HYPERTENSION

Marili Niglas, Nicoleta Baxan, Ali Ashek, Lin Zhao, Jinming Duan, Declan O'Regan, Timothy J W Dawes, Wenjia Bai, Lan Zhao

*National Heart and Lung Institute, Imperial College London, London; Biological Imaging Centre, Imperial College London, London; School of Computer Science, University of Birmingham, Birmingham; MRC London Institute of Medical Sciences, Imperial College London, London; Department of Brain Sciences, Imperial College London, London, UK*

Application of artificial intelligence to cardiac magnetic resonance (CMR) images improved ventricular phenotyping and survival projection of pulmonary arterial hypertension (PAH) patients by providing three‐dimensional (3D) right ventricular (RV) wall motion patterns. We present a rat‐specific pipeline that enables automated bi‐ventricular segmentations and motion analysis. We acquired cine (*n* = 118) and 3D (*n* = 8) CMR datasets longitudinally from rats treated with monocrotaline (MCT; *n* = 24) or Sugen with 4‐weeks hypoxia (SuHx; *n* = 43). The data set was randomly split into 76 training and 42 test sets. A fully convolutional network applied to 42 MCT and SuHx cine datasets generated bi‐ventricular automated segmentations in addition to epi‐ and endocardial meshes. Global function was derived from automated segmentations and compared with manual segmentations as ground truth. The RV regional contractile patterns were mapped from end‐diastolic and end‐systolic meshes and corrected to end‐diastolic volume. Our pipeline produced qualitatively and quantitatively equivalent two‐dimensional segmentations in <5 s. The average difference for end‐diastolic volume and end‐systolic volume was −2.2% (±3.3%; 13.1 ± 23.0 μL) and 2.7% (±4.8%; 11.8 ± 26.5 μL), respectively. The RV contractile pattern in a healthy animal was heterogeneous, where highest motion was observed in the base. When compared with healthy rats, the basal motion progressively declined by 26% (95% confidence interval [−21.1%, −33.6%]) and 46% [−29.6%, −73.6%] at 2 and 4 weeks MCT, respectively. The basal motion in SuHx rats declined to nearly half‐fold of healthy values. Although the contraction at the base at 4 weeks was maintained at the 6‐week time point (−47.0% [−27.7%, −80.5%] and 47.3% [−40.8%, −50,7%], respectively), the values started to deteriorate to a greater extent at 8 weeks (−60.4% [−56.0%, −68.1%]). The rat‐specific pipeline produced fast and accurate cardiac segmentations. Contraction analysis revealed a declining RV wall motion in PH rats. Furthermore, the basal RV wall motion remodelling bias in the rats is consistent with reported findings from PAH patients.

## A143 THERAPEUTIC EFFECTS OF RENIN‐ANGIOTENSIN SYSTEM‐INHIBITING DRUGS LOSARTAN AND ENALAPRIL ON SARS‐COV‐2 INFECTION IN EX VIVO CULTURED HUMAN PRECISION‐CUT LUNG SLICES

Martina Korfei, Christin Müller, Clemens Ruppert, Poornima Mahavadi, Ruth Charlotte Dartsch, Peter Dorfmüller, Stefan Gattenloehner, Stefanie Dimmeler, Elie El Agha, Saverio Bellusci, Werner Seeger, Susanne Herold, Biruta Witte, John Ziebuhr, Andreas Guenther

*Department of Internal Medicine II and Biomedical Research Center Seltersberg (BFS), Justus Liebig University Giessen, Giessen; Universities of Giessen and Marburg Lung Center (UGMLC), Member of the German Center for Lung Research (DZL) Giessen; Institute of Medical Virology, Institute of Pathology, Justus Liebig University Giessen, Giessen; German Center for Infection Research (DZIF), Department of Internal Medicine II and Biomedical Research Center Seltersberg (BFS), Justus Liebig University Giessen, Giessen; Institute for Cardiovascular Regeneration, Goethe University Frankfurt, Frankfurt; Institute for Lung Health (ILH), Giessen; Max Planck Institute for Heart and Lung Research, Department of Lung Development and Remodeling, Bad Nauheim; Division of Infectious Diseases, Pulmonary and Critical Care Medicine, Department of Internal Medicine II, University Hospital of Giessen, D‐35392 Giessen; Department of General and Thoracic Surgery, University Hospital Giessen, D‐35392 Giessen; Lung Clinic, Evangelisches Krankenhaus Mittelhessen, D‐35398 Giessen, Germany*

The role of the renin–angiotensin system (RAS) in coronavirus disease 2019 (COVID‐19) has received much attention, because the angiotensin‐converting enzyme 2 (ACE2) has been identified as the main receptor for the severe acute respiratory syndrome coronavirus 2 (SARS‐CoV‐2). It has been speculated that, in COVID‐19, RAS dysregulation in favor of angiotensin II (Ang‐II)‐mediated signaling might result in severe tissue inflammation and lung injury. Likewise, the role of a pre‐existing therapy with angiotensin‐converting enzyme 1 inhibitors (ACEi) or Ang‐II type 1 receptor blockers (ARBs) in COVID‐19 is largely unclear. We evaluated the effects of the ACEi enalapril (ENA) and the ARB losartan (LOS) on SARS‐CoV‐2 infection in human *ex vivo*‐cultured, precision‐cut lung slices (PCLS) obtained from normal human lung tissue. The PCLS were pretreated for 5 days with vehicle, LOS or ENA (300 µM), followed by mock infection or infection with SARS‐CoV‐2 and (continued) incubation with vehicle, LOS or ENA for 1 or 2 days. Thereafter, PCLS were harvested for analysis of viral replication, inflammatory responses, endoplasmic reticulum (ER) stress and apoptosis pathways. Both LOS and ENA significantly reduced viral replication in PCLS, with ENA being more potent. LOS was more efficient than ENA in reducing the expression of *IL1B*, *CCL2*, *CXCL2* and *TNFA*, but not of *IL6*, whereas ENA preferentially caused a reduction of *IL6* and *CCL2* in SARS‐CoV‐2‐infected PCLS. Furthermore, ENA, but not LOS, significantly decreased the expression of viral entry factors, ACE2 and transmembrane serine protease 2 (TMPRSS2), in infected PCLS, both of which were found to be robustly induced upon SARS‐CoV‐2 infection. Importantly, LOS or ENA did not exert apoptosis or other cytotoxic effects. Renin–angiotensin system‐antagonizing drugs do not seem to exert detrimental effects during SARS‐CoV‐2 infection. On the contrary, in an *ex vivo* model of human PCLS, such treatment was found to dampen SARS‐CoV‐2 infection and consecutive inflammation.

## A144 DEFINING INFLAMMATORY PHENOTYPES IN PATIENTS WITH PULMONARY HYPERTENSION

Anže Žgank, Matevž Harlander, Matjaž Turel, Polona Mlakar, David Lestan, Janez Toplišek, Vojka Gorjup, Barbara Salobir

*University Clinical Centre Ljubljana, Faculty of Medicine, University of Ljubljana, Ljubljana, Slovenia*

Standard classification of pulmonary hypertension (PH), dividing patients into five classes based on etiology, is useful for diagnosis and treatment. However, it does not explain why some patients do not respond to therapy and progress more rapidly. It seems that chronic inflammation might explain, at least in part, the above‐mentioned difference. Thus, the aim of our study was to explore inflammatory markers further and try to find different inflammatory phenotypes. In 135 patients (83 female) referred to our PH center (one of two in Slovenia, with only 2 million inhabitants) for further evaluation of PH between 2004 and 2017, we also measured markers of inflammation. Besides classical C‐reactive protein (CRP), the first marker was serum uric acid (SUA), a marker of metabolic syndrome connected with chronic inflammation. The second one was serum amyloid alpha (SAA), which has not been investigated in depth in PH. We correlated them between each other and assessed the influence of each on survival by using Kaplan–Meier analysis. The CRP, SUA and SAA medians were in normal ranges, but distribution was uneven, with wide interquartile ranges. Patients with SUA (Q4 = 463.5; log rank χ^2^ = 22.654, *p* < 0.001) and SAA (Q4 = 11.95; log rank χ^2^ = 7.782, *p* = 0.005) in the upper quartile (Q4) have worse prognosis than others. There was no similar influence on survival for CRP (Q4 = 6.75; log rank χ^2^ = 1.699, *p* = 0.192, n.s.). Both SUA and SAA were significantly correlated with CRP (*r* = 0.24, *p* = 0.01 and *r* = 0.45, *p* = 0.000) but not with each other (*r* = 0.07, *p* = 0.47). Based on our results, we propose two distinct inflammatory phenotypes associated with worse prognosis. The first inflammatory phenotype is associated with SUA, which is a marker of inflammation attributable to metabolic syndrome, and the second inflammatory phenotype is associated with increased SAA, which needs to be explored further. Both SUA and SAA were better predictors of worse prognosis than CRP in the whole group of PH patients.

## A145 SEXUAL DIMORPHISM IN THE IMMUNE SYSTEM IN GROUP 1 PULMONARY HYPERTENSION: DOES IT PLAY A ROLE IN RIGHT VENTRICULAR FAILURE?

Patricia D A Lima, Ruaa Al‐Qazazi, Francois Potus, Ashley Martins, Oliver Jones, Stephen L Archer

*Department of Medicine, Queen's University, Kingston, ON; Queen's Cardiopulmonary Unit, Queen's University, Kingston, ON; Pulmonary Hypertension Research Group, Institut Universitaire de Cardiologie et de Pneumologie de Québec Research Center, Laval University, Quebec City, QC, Canada*

Although there is female preponderance in pulmonary arterial hypertension (PAH), males have a worse prognosis owing to right ventricular failure (RVF). Given that sexual dimorphism in the immune response also exists, we asked whether monocytes and macrophages are differentially regulated in males versus females and whether such a difference underlies the male predisposition to RVF. The immune response differs between males and females in PAH. A higher number of circulating monocytes and right ventricular (RV) macrophages in males might contribute to greater RV inflammation and RVF predisposition. A PAH‐like phenotype was induced in male and female Sprague–Dawley rats (*n* = 6/group/sex) using monocrotaline (MCT; 60 mg/kg). Right ventricular function was assessed using echocardiography and hemodynamics. Circulating classical (CD3^−^B220^−^SSA^lo^His48^lo^CD43^lo^; inflammatory) and nonclassical (CD3^−^B220^−^SSA^lo^His48^lo^CD43^hi^; anti‐inflammatory) monocytes, and RV and lung macrophages (CD45^+^CD68^+^) were quantified via flow cytometry. Right ventricular fibrosis was assessed with Picrosirius Red staining. Both male and female MCT rats, showed significantly increased right ventricular systolic pressure (*p* = 0.0007 and *p* = 0.003) and reduced pulmonary artery acceleration time (*p* = 0.0001 and *p* = 0.001) compared with controls. Although right ventricular end‐diastolic pressure (*p* = 0.0009 and *p* = 0.03) was high and cardiac output (*p* = 0.0007 and *p* = 0.007) low in both sexes, the ejection fraction and tricuspid annular plane systolic excursion were significantly reduced only in MCT males (MCT, 50.1 ± 8.9 vs. PBS, 76.8 ± 10; *p* = 0.01) and (MCT, 1.56 ± 0.55 vs. PBS, 2.68 ± 0.31; *p* = 0.002), respectively. Inflammatory (*p* = 0.007) and anti‐inflammatory (*p* = 0.001) monocytes were increased in MCT males, but not in females. Macrophages were also increased in the RV (MCT, 45.4 ± 3.1 vs. PBS, 35.2 ± 2.9; *p* = 0.007) and the lungs of MCT males (MCT, 37.8 ± 11.2 vs. PBS, 10.9 ± 3.5; *p* = 0.04). The number of RV macrophages was predictive of the severity of fibrosis in males (*p* = 0.0009). Right ventricular function is worse in MCT males than females, and it is associated with a higher incidence of monocytes, RV macrophages and fibrosis. Understanding the sex‐specific immune response in PAH is crucial in developing new therapeutics addressing inflammation and RVF.

## A146 THE PROGNOSTIC IMPLICATIONS OF PULMONARY HYPERTENSION IN SYSTOLIC HEART FAILURE IN A RESOURCE‐CONSTRAINED SINGLE CENTER: THE SOUTHERN AFRICA HEART FAILURE REGISTRY AND EPIDEMIOLOGICAL STUDY (SAHFRES)

Mamotabo Matshela, Nonhle Lushozi

*University of KwaZulu‐Natal, Durban; Mediclinic Heart Hospital, Arcardia; Durban University of Technology, Durban, South Africa*

Heart failure (HF) affects millions of people worldwide and is associated with recurrent hospitalization and prolonged hospital stay, leading to high hospital expenditure. The aim of this study was to assess the prognostic implications of pulmonary hypertension (PH) in HF with reduced ejection fraction (HFrEF) in a single‐centre setting to improve the HF registry. We also wanted to assess the association between HFrEF and primary contributing factors to PH in HFrEF. A total of 125 patients (44% males, mean age 56 years) with HFrEF, left ventricular ejection fraction (LVEF) < 50%, were included in the study. Data were accessible and re‐evaluated prospectively, between 1 January 2016 and 30 June 2018. Patients were divided based on ethnicity, severity of left ventricular (LV) dysfunction, severity of PH and HF symptoms. Survival was evaluated and reported using the Kaplan–Meier curve. This was an age‐ and gender‐matched study. The mean LVEF was 37 (9%). The study population came predominantly from impoverished communities: 73% African, 13% Indian, 12% white and 2% were of color. A strong relationship between the severity of LVEF impairment, severity of PH, black ethnicity, poor socio‐ economic status and symptoms was demonstrated. Severely impaired LV systolic function was associated with severe symptoms, low socioeconomic class, black ethnicity and presence of severe PH (*p* < 0.001). The major risk factors for the development of LV dysfunction and concomitant PH were hypertension, diabetes mellitus, mitral valve disease and older age (*p* < 0.0001). The following factors were associated with poor survival: severely impaired LV function, severe symptoms (New York Heart Association ≥ II), low socioeconomic status, black ethnicity and severe PH. Low socioeconomic class, black ethnicity, severely impaired LV function and severe symptoms are associated with poor survival, warranting further and larger studies for further screening and optimal management of PH in patients with HFrEF.

## A147 THE PREVALENCE AND PROGNOSTIC IMPLICATIONS OF PULMONARY HYPERTENSION IN CONSTRICTIVE PERICARDITIS

Mamotabo Matshela

*University Of KwaZulu‐Natal, Durban; Mediclinic Heart Hospital, Arcardia, South Africa*

Constrictive pericarditis (CP) is a pathological process leading to scarring and thickening of the pericardium, compression of cardiac chambers and diastolic dysfunction. Pericardiectomy remains an acceptable and curative surgical procedure in most patients. Pulmonary hypertension (PH) is defined as a mean pulmonary arterial systolic pressure (mPASP) >25 mmHg at rest and >30 mmHg during exertion. The prevalence of PH in CP is certainly considered rare; as a result, the main aim of this study was to evaluate the prevalence and prognostic factors of PH in CP. A total of 264 patients (54% males, mean age 46 years) with CP were included for this age‐ and gender‐matched study. Data were evaluated retrospectively, between 1 January 2016 and 31 December 2018. Patients were divided based on the severity of PH and HF symptoms. Survival was evaluated and reported using the Kaplan–Meier curve. The presence of PH was demonstrated in 27% of the study population. The following factors were demonstrated to be associated with the presence or severity of PH in CP, including older age, prior cardiac surgery, pre‐existing PH, primary etiology of CP particularly prior radiotherapy, connective tissue diseases, chronic respiratory diseases and myocardial diseases. The following three factors were associated with severe PH: prior cardiovascular surgery, chronic respiratory diseases and prior radiotherapy; then to a lesser extent followed by valvular heart diseases, significant left ventricular systolic dysfunction and tricuspid regurgitation. The following factors were associated with poor overall survival: prior radiotherapy, older age and ventricular dysfunction.

One‐third of the study population presented with PH and multiple factors demonstrated an associated with outcomes, particularly poor survival, warranting further and larger studies for further screening and optimal management of PH in CP.

## A148 RIGHT VENTRICULAR STRAIN IN PULMONARY HYPERTENSION

Mamotabo Matshela

*University Of KwaZulu‐Natal, Durban; Mediclinic Heart Hospital, Arcardia, South Africa*

Abnormal right ventricular (RV) filling patterns have been reported in pulmonary hypertension (PH), which improve over time. Despite that, data on the use of strain to evaluate RV function in PH is still rather limited. As a result, this study was conducted to assess variations in RV strain parameters in PH and the association of RV strain parameters/variations with survival. A total of 102 patients (47% males, mean age 58 years) with left ventricular ejection fraction ≥50% where included in the study. Data were accessible and re‐evaluated prospectively, between 1 January 2016 and 30 June 2017. Patients were divided based on the severity of PH and heart failure symptoms. Survival was evaluated and reported using the Kaplan–Meier curve. This was an age‐ and gender‐matched study. Segmental and wall mean (±SD) values for RV longitudinal stan parameters were evaluated. The baseline global RV free wall (RVFWS) systolic strain values were low in all PH groups compared with standard normal cohorts (mean of −20.4). Normal mean pulmonary arterial systolic pressure (mPASP) or mild PH was associated with a significantly increased or higher RVFWS strain, predominantly in young patients only, compared with severe PH. The presence or severity of tricuspid regurgitation (TR) was predictive of changes in RVFWS strain, because patients with at least moderate TR demonstrated lower RVFWS compared with no TR. The strain amplitudes remained low in those with severe PH or TR during follow‐up. Patients with presence of at least moderate severity of TR/PH before medical therapy demonstrated less improvement in RVFWS values compared with patients who were assumed to have mild or no TR (PH) based on two‐dimensional transthoracic echocardiographic assessment during the short‐term follow‐up. Low RVFWS strain values based on median distribution were associated with poor overall survival. In addition, less improvement in RVFWS on optimal medical therapy was also associated with poor survival. The following factors were independent predictors of survival while on medical therapy: older age, prior cardiac surgery, low baseline Tricuspid annular plane systolic excursion (TAPSE) and at least moderate TR. After multivariate adjustment using different models, low baseline and follow‐up based on median change of RVFWS, presence of pulmonary diseases, high mPASP and low TAPSE were associated with poor survival. The presence of PH is associated with lower RVFWS, more so in those with at least moderate PH in severity. At least moderate TR/PH demonstrated low RVFWS strain values predictive of poor survival, which would guide other treatment options, particularly in patients with concomitant independent predictors of survival.

## A149 COMPERA 2.0 RISK STRATIFICATION IN MEDICALLY MANAGED CHRONIC THROMBOEMBOLIC PULMONARY HYPERTENSION

Harrison Stubbs, Stephanie Luam, Melanie Brewis, Martin Johnson, Colin Church

*Scottish Pulmonary Vascular Unit, Golden Jubilee National Hospital, University of Glasgow*

The COMPERA 2.0 model has been validated in pulmonary arterial hypertension yet awaits validation in other causes of pulmonary hypertension, including chronic thromboembolic pulmonary hypertension (CTEPH). Compared to the current 3‐strata risk model of low‐, intermediate‐ and high‐risk, COMPERA 2.0 adds a fourth stratum and uses non‐invasive clinical variables. A retrospective analysis of all patients diagnosed with medically‐managed CTEPH between 2010 and 2020 was performed at the Scottish Pulmonary Vascular Unit. Patients were risk‐stratified using the 3‐strata and novel 4‐strata risk models at diagnosis and first follow‐up using functional class, 6‐minute walk distance and N‐terminal prohormone brain natriuretic peptide (NT‐proBNP). The association between risk and survival was analysed using Kaplan‐Meier analysis with log‐rank and Cox Proportional Hazard regression modelling.

At baseline (*n* = 128) the 4‐strata model delineated 4 distinct prognostic groups; survival at 1‐ and 5‐ years respectively was 100%, 100% for low risk, 93%, 57% for intermediate‐low risk, 91.5%, 42.8% for intermediate‐high risk and 83.3%, 22.9% for high risk (*p* < 0.0001). The 3‐strata model had the majority of patients in intermediate risk (60.1%) whilst the 4‐strata delineated the groups more evenly; 6.3% low, 25% intermediate‐low, 49% intermediate‐high and 20% high risk. Changes in risk from baseline to first follow‐up was seen in 33% of patients with the 4‐strata model and 44.3% for the 3‐strata model. Survival data at follow up demonstrated 1‐ and 5‐year survival calculated at 100%, 100% for low risk, 96.6%, 70.4% for intermediate‐low risk, 97.9%, 40.8% for intermediate‐high risk and 85%, 40.1% for high risk (*p* < 0.0001). The 4‐strata model was a better discriminator of mortality than the 3‐strata model at follow up.

Despite relatively small numbers in this study, the COMPERA 2.0 model is a valid tool for risk prediction in medically‐managed CTEPH with survival data more discriminatory at baseline and follow up.

